# The coronavirus proofreading exoribonuclease mediates extensive viral recombination

**DOI:** 10.1371/journal.ppat.1009226

**Published:** 2021-01-19

**Authors:** Jennifer Gribble, Laura J. Stevens, Maria L. Agostini, Jordan Anderson-Daniels, James D. Chappell, Xiaotao Lu, Andrea J. Pruijssers, Andrew L. Routh, Mark R. Denison

**Affiliations:** 1 Department of Pathology, Microbiology, and Immunology, Vanderbilt University Medical Center, Nashville, Tennessee, United States of America; 2 Vanderbilt Institute for Infection, Immunology, and Inflammation (VI4), Vanderbilt University Medical Center, Nashville, Tennessee, United States of America; 3 Department of Pediatrics, Vanderbilt University Medical Center, Nashville, Tennessee, United States of America; 4 Department of Biochemistry and Molecular Biology, University of Texas–Medical Branch, Galveston, Texas, United States of America; 5 Sealy Center for Structural Biology and Molecular Biophysics, University of Texas–Medical Branch, Galveston, Texas, United States of America; Johns Hopkins University Bloomberg School of Public Health, UNITED STATES

## Abstract

Recombination is proposed to be critical for coronavirus (CoV) diversity and emergence of SARS-CoV-2 and other zoonotic CoVs. While RNA recombination is required during normal CoV replication, the mechanisms and determinants of CoV recombination are not known. CoVs encode an RNA proofreading exoribonuclease (nsp14-ExoN) that is distinct from the CoV polymerase and is responsible for high-fidelity RNA synthesis, resistance to nucleoside analogues, immune evasion, and virulence. Here, we demonstrate that CoVs, including SARS-CoV-2, MERS-CoV, and the model CoV murine hepatitis virus (MHV), generate extensive and diverse recombination products during replication in culture. We show that the MHV nsp14-ExoN is required for native recombination, and that inactivation of ExoN results in decreased recombination frequency and altered recombination products. These results add yet another critical function to nsp14-ExoN, highlight the uniqueness of the evolved coronavirus replicase, and further emphasize nsp14-ExoN as a central, completely conserved, and vulnerable target for inhibitors and attenuation of SARS-CoV-2 and future emerging zoonotic CoVs.

## Author summary

Recombination is an essential part of normal coronavirus replication, required for the generation of the sub-genomic mRNAs as well as defective viral genome (DVGs) and is also implicated in novel strain emergence. However, the molecular mechanisms and determinants of RNA recombination in CoVs are unknown. Here, we compare recombination in 3 divergent beta-coronaviruses; murine hepatitis virus (MHV), MERS-CoV, and SARS-CoV-2. We show that they have striking similarities in the populations of RNA produced and in the sequences surrounding recombination junctions. Further, we demonstrate that the coronavirus proofreading exoribonuclease (nsp14-ExoN) is required to maintain the rates and loci of recombination generated during infection. These data suggest that recombination and the coronavirus exoribonuclease are conserved and important determinants of replication that may be targeted for inhibition and attenuation to control the ongoing pandemic of SARS-CoV-2 and prevent future outbreaks of novel coronaviruses.

## Introduction

The ongoing severe global pandemic of SARS-CoV-2, the etiological agent of coronavirus disease 2019 (COVID-19) underlines the importance of defining the determinants of coronavirus (CoV) evolution and emergence into human populations [1]. Studies comparing CoV strains that are closely related to SARS-CoV-2 have proposed that SARS-CoV-2 acquired the ability to infect human cells through recombination within the spike protein sequence [2–4]. Further, a study of genetic variation in patient SARS-CoV-2 samples has suggested that recombination may be occurring during infections in humans [5]. Recombination is also implicated in the emergence of severe acute respiratory syndrome coronavirus (SARS-CoV) and Middle East respiratory syndrome coronavirus (MERS-CoV) [6–10]. Together, these data support the hypothesis that generation of novel CoVs, cross-species movement, and adaptation may be driven by recombination events in nature. CoV recombination has been reported to be associated with increased spread and severe disease, and has resulted in vaccine failure of multiple livestock CoVs [11,12]. Thus, targeting the ability of the virus to recombine is a critical consideration for vaccine development in the ongoing SARS-CoV-2 pandemic as well as future animal and zoonotic CoVs.

Coronaviruses are a family of positive-sense, single-stranded RNA viruses with genomes ranging in size between 26 and 32 kb (S1A Fig). During normal replication, the putative CoV replication-transcription complex (RTC), formed by multiple nonstructural proteins (nsp) encoded in ORF1ab, drives RNA synthesis and encompasses many enzymatic functions [13–16]. Previous reports indicate that CoVs readily perform both inter-molecular recombination between 2 distinct molecules and intra-molecular recombination within the same molecule (S1B Fig). Co-infection with related strains of the model β-CoV murine hepatitis virus (MHV) results in chimeric viral genomes that are generated by inter-molecular recombination [17,18]. The CoV RTC performs intra-molecular recombination at virus-specific transcription regulatory sequences (TRSs) to generate a set of subgenomic mRNAs (sgmRNAs) with common 5’ and 3’ ends (S1A and S1B Fig) [19,20]. sgmRNAs are subsequently translated into structural and accessory proteins [19]. CoVs also generate defective viral genomes (DVGs) that contain multiple deletions of genomic sequence while retaining intact 5’ and 3’ genomic untranslated regions (5’ and 3’ UTRs). DVGs are amplified by RTC machinery supplied by co-infecting full-length helper CoVs [21–24]. DVGs in respiratory viruses can act as pathogen-associated molecular patterns (PAMPs) and stimulate the innate immune system [25,26]. The role of DVGs in CoV biology is largely unknown, although some DVGs interfere with viral replication [27,28]. Therefore, CoVs perform recombination as a normal part of their replication, producing complex populations of recombined RNA molecules. Prior to the advent of Next Generation Sequencing (NGS), direct analysis of recombined CoV RNAs was not possible and the determinants of recombination could not be identified.

In other RNA virus families including picornaviruses and alphaviruses, regulation of recombination has been mapped to replication fidelity determinants in the viral RNA-dependent RNA polymerase (RdRp) [29–32]. In contrast to these viruses, CoV replication fidelity is primarily determined by the 3’-to-5’ exoribonuclease encoded in nonstructural protein 14 (nsp14-ExoN) that proofreads RNA during replication through excision of mismatched incorporated nucleotides [33–38]. Viral exonucleases are essential for recombination in DNA viruses, including vaccinia virus and herpes simplex virus 1 [39,40]. In contrast, a role of the nsp14-ExoN in CoV RNA recombination had not previously been defined. In our lab, viral mutants of MHV with engineered inactivation of nsp14-ExoN (ExoN(-)) resulted in reduced abundance of sgmRNA2. In another program, rescue of viable ExoN(-) human CoV 229E (HCoV-229E) was unsuccessful, but limited replication was associated with decreased detection of sgmRNAs [34,41]. Although these reports did not study recombination or molecular mechanisms, they support the hypothesis that CoV nsp14-ExoN activity RNA synthesis and possibly recombination, in addition to the known functions of nsp14-ExoN in CoV replication fidelity, viral fitness, *in vivo* virulence, resistance to nucleoside analogues, and immune antagonism [36,42,43].

In this study, we sought to define the frequency and patterns of recombination of divergent β-CoVs SARS-CoV-2, MERS-CoV, and MHV, and to test the role of nsp14-ExoN in recombination. We used both short-read Illumina RNA-sequencing (RNA-seq) and long-read direct RNA Nanopore sequencing for all three viruses to show that they perform extensive recombination during replication *in vitro* with broadly similar patterns of recombination, and generate diverse yet similar populations of recombined molecules. We further demonstrate that genetic inactivation of MHV nsp14-ExoN results in a significant decrease in recombination frequency, altered recombination junction patterns across the genome, and altered junction site selection. These defects and alterations result in a marked change in MHV-ExoN(-) recombined RNA populations, including defective viral genomes (DVGs). These results support future studies aimed at illuminating the role of SARS-CoV-2 nsp14-ExoN activity in RNA recombination, the regulation of sgmRNA expression, and its contribution to novel CoV zoonotic emergence. Combined with the multiple critical integrated functions of nsp14-ExoN, the role in recombination further defines nsp14-ExoN as a conserved, vulnerable, and highly specific target for inhibition by antiviral treatments and viral attenuation.

## Results

### SARS-CoV-2 and MERS-CoV generated extensive populations of recombination junctions

We first sought to quantify recombination frequency and identify recombination patterns in zoonotic CoVs by sequencing both MERS-CoV and SARS-CoV-2 RNA. In three independent experiments for each virus, Vero cell cultures were infected with either MERS-CoV or SARS-CoV-2 until the monolayer displayed >70% virus-induced cytopathic effect (CPE). Total RNA from infected cells was isolated and poly(A)-selected to capture all viral RNA containing poly-A tails, including genomic, subgenomic, and defective viral genome (DVG) RNA molecules. Equal amounts of total cell RNA from each of the three independent experiments for each virus was sequenced by short-read Illumina RNA-sequencing (RNA-seq), and by long-read direct RNA Nanopore sequencing. The depth and low error rate of RNA-seq facilitated the detection and quantification of both high- and low-abundance unique junctions. Long-read direct RNA sequencing on the Oxford Nanopore Technologies MinION platform was used to sequence complete RNA molecules, to define the organization of junctions in the context of intact RNA molecules. By comparing short- and long-read RNA sequencing, we accomplished high-confidence detection and quantification of recombination junctions as well as description of the genetic architectures of molecules formed by the junctions.

For RNA-seq, reads were aligned to the respective viral genomes (S1A Fig) using a recombination-aware mapper, *ViReMa* (*Vi*rus *Re*combination *Ma*pper) [44]. *ViReMa* detected recombination events that generated deletions greater than 5 base-pairs and that were flanked by a 25 base-pair alignment both upstream and downstream of the junction site. *ViReMa*-detected junctions may be formed from either inter-molecular or intra-molecular recombination during replication. *ViReMa* aligned both recombined and non-recombined reads in the library and reported the total number of nucleotides aligned to the genome and all detected recombination junctions.

**Fig 1:**
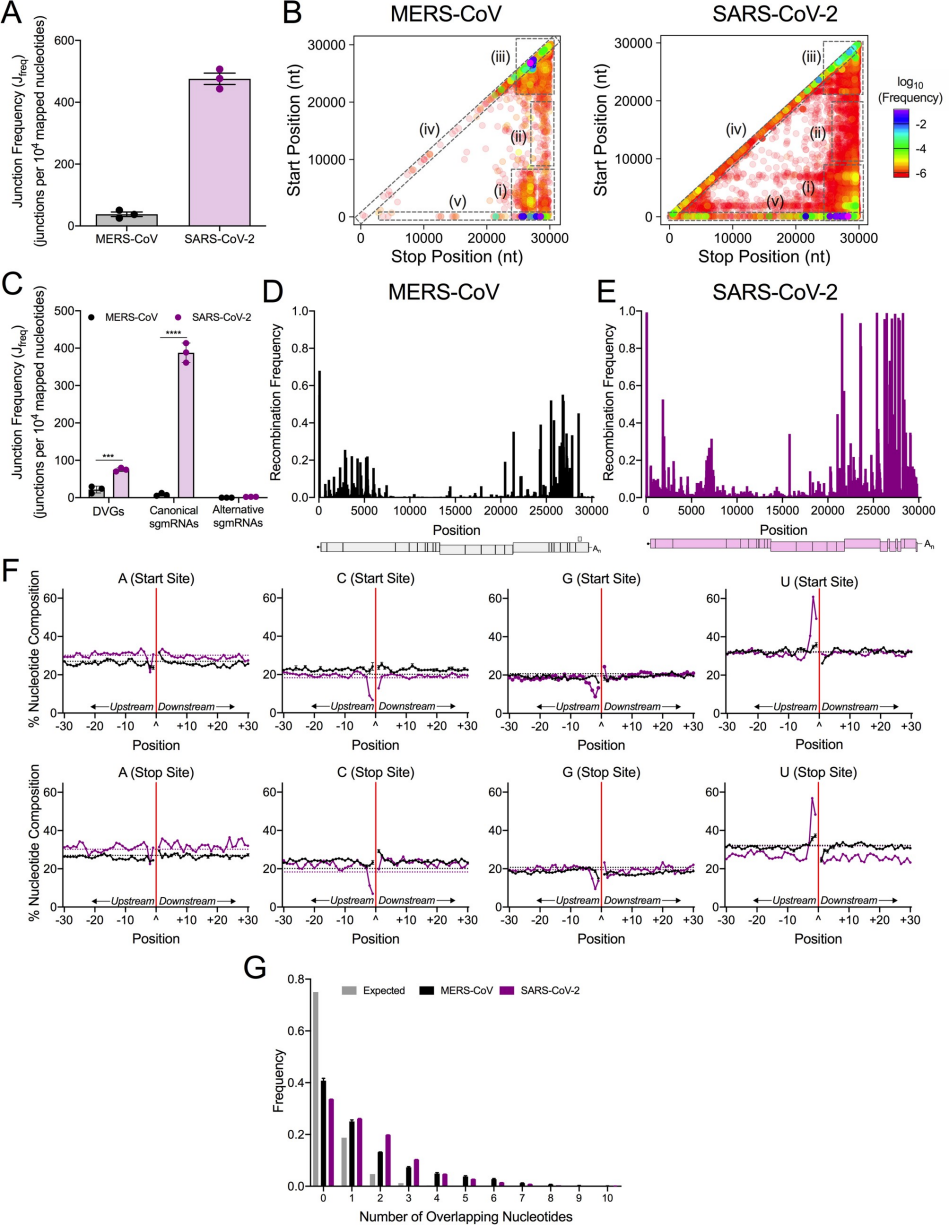
Genome-wide recombination generates populations of diverse RNA molecules in MERS-CoV and SARS-CoV-2. MERS-CoV total cell lysates (black) and SARS-CoV-2 infected cell monolayers (violet) were sequenced by RNA-seq. (A) Junction frequency (J_freq_) was calculated by normalizing number of nucleotides in *ViReMa*-detected junctions to viral RNA (total mapped nucleotides) and multiplying by 10,000 to express J_freq_ as the number of junctions per 10^4^ mapped nucleotides. Error bars represent standard errors of the mean (SEM) for three independent sequencing libraries (N = 3). (B) Recombination junctions are mapped according to their genomic position (5’ junction site, Start Position; 3’ junction site, Stop Position) and colored according to their frequency in the population of all junctions in MERS-CoV and SARS-CoV-2. The highest frequency junctions are magenta and completely opaque. The lowest frequency junctions are red and the most transparent. Dashed boxes represent clusters of junctions: (i) 5’ ➔ 3’; (ii) mid-genome ➔ 3’ UTR; (iii) 3’ ➔ 3’; (iv) local deletions; (v) 5’ UTR ➔ rest of genome. (C) The J_freq_ of DVGs, canonical sgmRNAs, and alternative sgmRNAs was calculated and compared in MERS-CoV (black) and SARS-CoV-2 (violet). Error bars represent SEM for 3 independent sequencing libraries (N = 3) of each virus. 2-way ANOVA with multiple comparisons corrected by statistical hypothesis testing (Sidak test). *** p < 0.001, **** p < 0.0001. Mean recombination frequency is quantified at each position across the MERS-CoV (D) and SARS-CoV-2 (E) genomes (N = 3). Recombination frequency was calculated by dividing the number of nucleotides in detected junctions at that position (start and stop sites) by the total number of mapped nucleotides at the position. See also S2 Fig and S1 Table. (F) The percent adenosine (A), cytosine (C), guanine (G), and uracil (U) at each position in a 30-base pair region flanking DVG junction start and stop sites in MERS-CoV (black) and SARS-CoV-2 (violet). Each point represents a mean (N = 3) and error bars represent SEM. The junction site is denoted as a carat (^) and with a solid red line. Positions upstream from the junction are labelled -30 to -1 and positions downstream are labelled +1 to +30. The expected nucleotide percentage based on the composition of the viral genome is marked as a dashed line (black = MERS-CoV, violet = SARS-CoV-2). (G) Distribution of sequence microhomology in MERS-CoV (black) and SARS-CoV-2 (violet) compared to an expected probability distribution (gray). The frequency of each nucleotide overlap length is displayed as a mean (N = 3) and error bars represent SEM.

Alignment of MERS-CoV and SARS-CoV-2 with *ViReMa* demonstrated nearly identical read coverages for MERS-CoV (1118) and SARS-CoV-2 (1122) (S2A and S2B Fig). Further, 82.95% of MERS-CoV RNA-seq reads and 77.48% of SARS-CoV-2 reads mapped to the viral genome, demonstrating RNA-seq libraries in both viruses had a similar proportion of viral RNA (S1 Table). To quantify recombination, recombination junction frequency (J_freq_) was calculated for MERS-CoV and SARS-CoV-2 (Fig 1A). J_freq_ refers to the number of nucleotides in all detected junctions normalized to viral RNA amount in a sample (total mapped nucleotides). Thus, J_freq_ was not biased by the number of virus-mapping reads. J_freq_ was multiplied by 10^4^ to scale for library size and was reported as the number of junctions per 10^4^ mapped nucleotides. MERS-CoV had a mean J_freq_ of 37.80 junctions detected per 10^4^ mapped nucleotides. SARS-CoV-2 had a mean J_freq_ of 475.7 junctions per 10^4^ mapped nucleotides (Fig 1A). This was a surprising difference in J_freq_ between the two viruses that were infected at similar multiplicity of infections (MOIs), were collected when the cells displayed similar levels of CPE, and had similar viral abundance in sequenced RNA. We considered the possibility that the observed >10-fold difference between J_freq_ of each virus could be due to the replication capacity of the parental virus. We compared the number of unique junctions generated by each virus to remove any potential viral replication bias. SARS-CoV-2 generated an average of 56,082 unique junctions per experiment, while MERS-CoV generated an average of 19,367 unique junctions per experiment (S2C Fig). Thus, both the number of recombination junctions and J_freq_ were similarly higher in SARS-CoV-2 compared to MERS-CoV, suggesting that these differences are not solely due to an increased replication capacity or viral amplification of recombined species. This will be an important area for future study to determine if SARS-CoV-2 is associated with increased recombination in other cell types, *in vivo* models, or clinical samples. In any case, quantification of both recombination junction frequency and the number of unique recombination junctions in MERS-CoV and SARS-CoV-2 showed that both viruses produce abundant recombination junctions during replication in culture.

To define the patterns of the detected recombination junctions, we mapped forward (5’ ➔ 3’) recombination junctions according to their genomic position (Figs 1B and S2C and S2D). Both MERS-CoV and SARS-CoV-2 displayed clusters of junctions in multiple conserved patterns: 1) between the 5’ and 3’ ends of the genome; 2) between intermediate genomic positions and the 3’ end of the genome; 3) within the 3’ end of the genome; 4) representing local deletions across the genome; and 5) between the 5’ untranslated region (UTR) and the rest of the genome. (Fig 1B). SARS-CoV-2 also had many low-frequency junctions distributed across the genome and horizontal clusters of low-frequency junctions between common start sites at position ~2000 and ~8000 and the rest of the genome (Fig 1B). Overall, these data demonstrate that extensive RNA recombination during replication of both MERS-CoV and SARS-CoV-2 generates diverse populations of junctions with similar high-abundance clusters.

### MERS-CoV and SARS-CoV-2 recombination generated defective viral genomes and subgenomic mRNAs

We next sought to define and quantify the populations of recombined RNA molecules produced in both MERS-CoV and SARS-CoV-2. SARS-CoV-2 sgmRNAs were identified by the location of recombination junctions within previously defined 65 base-pair regions containing the transcription regulatory sequence (TRSs) of each sgmRNA [45]. Similarly, 65 base-pair windows were defined encompassing the MERS-CoV TRS core sequences for each sgmRNA. Junctions between the 5’ TRS-L and sgmRNA-specific TRS were filtered. The most abundant sgmRNAs were designated as “canonical”, and other sgmRNA species were designated “alternative sgmRNAs”. Recombination junctions outside of the TRS-L and the sgmRNA-specific TRSs were designated as DVG junctions.

For each virus, the frequencies of DVGs, canonical sgmRNAs, and alternative sgmRNAs were normalized to total virus RNA. For both MERS-CoV and SARS-CoV-2, canonical and alternative junctions were detected for all sgmRNAs (Figs 1C and S2E and S2F). MERS-CoV and SARS-CoV-2 alternative sgmRNA was detected at similar frequencies (Fig 1C). In contrast, SARS-CoV-2 generated significantly higher frequencies of DVGs and canonical sgmRNAs than MERS-CoV (Fig 1C).

We next calculated the mean recombination frequency at each genomic position by comparing the number of nucleotides in detected junctions (both start and stop sites) at that position, and normalized to nucleotide depth at that position. Further, we determined genomic positions with a mean recombination frequency greater than 50% (Fig 1D and 1E). In MERS-CoV, there were 5 positions >50%; 4 of these mapped to TRS positions and 1 position was located in ORF5 (Fig 1D). In SARS-CoV-2, there were 26 positions with >50% recombination frequency, with13 mapping to TRS positions. SARS-CoV-2 also had high recombination frequency at positions in the nsp2 coding sequence, the S gene, M gene, and N gene (Fig 1E). In summary, the genomic positions with the highest frequency for both MERS-CoV and SARS-CoV-2 mapped to TRSs that form sgmRNA leader-body junctions. However, positions with high recombination frequency were identified at other locations across the genomes and relatively more in SARS-CoV-2 than MERS-CoV.

### MERS-CoV and SARS-CoV-2 defective viral genomes demonstrated distinct nucleotide compositions in the sequences flanking junctions

For both SARS-CoV-2 and MERS-CoV, the nucleotide composition of the start and stop sequences resulting in junctions forming DVGs in MERS-CoV and SARS-CoV-2 was determined and compared to the expected nucleotide percentage based on the parental viral genomes (Fig 1F). Sequences upstream (-30 to -1) and downstream (+1 to +30) of both the genomic start and stop sites of DVG junctions were analyzed. DVGs formed by junctions would contain sequences upstream of the start site (-30 to -1) and downstream of the stop site (+1 to +30) (S1C Fig). For both MERS-CoV and SARS-CoV-2, start and stop sequences upstream of the junction were enriched for uracil (U) and depleted for adenosine (A) and guanine (G). Downstream of the junction in both start and stop sites, both viruses were enriched for guanine (G) and adenosine (A) and depleted for uracil (U). MERS-CoV demonstrated a preference for U(U/C)^(G/A/C)(A/C)C in DVG start sites and UU^(G/C/A)C(G/C) in DVG stop sites. SARS-CoV-2 DVG sequences favored AUUU^(G/A)AAA in the start site sequences and ACUU^G(C/A)(C/A) in the stop site sequences. The nucleotide composition of MERS-CoV and SARS-CoV-2 differ from TRS-like sequences of MERS-CoV (AACGAA) [46] and SARS-CoV-2 (ACGAAC) [47], and therefore represent a selection of separate sequences for DVG formation.

### MERS-CoV and SARS-CoV-2 exhibited sequence microhomology at recombination junctions

We next tested whether MERS-CoV and SARS-CoV-2 junction sites favored regions of sequence microhomology at recombination junctions, defined as 2–20 nt regions of identical overlap [48]. The distribution of frequencies of 0–10 overlapping nucleotides at the start and stop sites of detected recombination junctions in both MERS-CoV and SARS-CoV-2 were compared to an expected probability distribution. Both MERS-CoV and SARS-CoV-2 junction sites demonstrated increased frequencies of overlaps of 2–7 nt (Fig 1G). Thus, MERS-CoV and SARS-CoV-2 favor the formation of recombined RNAs at junction sites exhibiting sequence microhomology.

### Direct RNA Nanopore sequencing of MERS-CoV and SARS-CoV-2 defined the architecture of full-length genome, sgmRNAs, and DVGs

**Fig 2:**
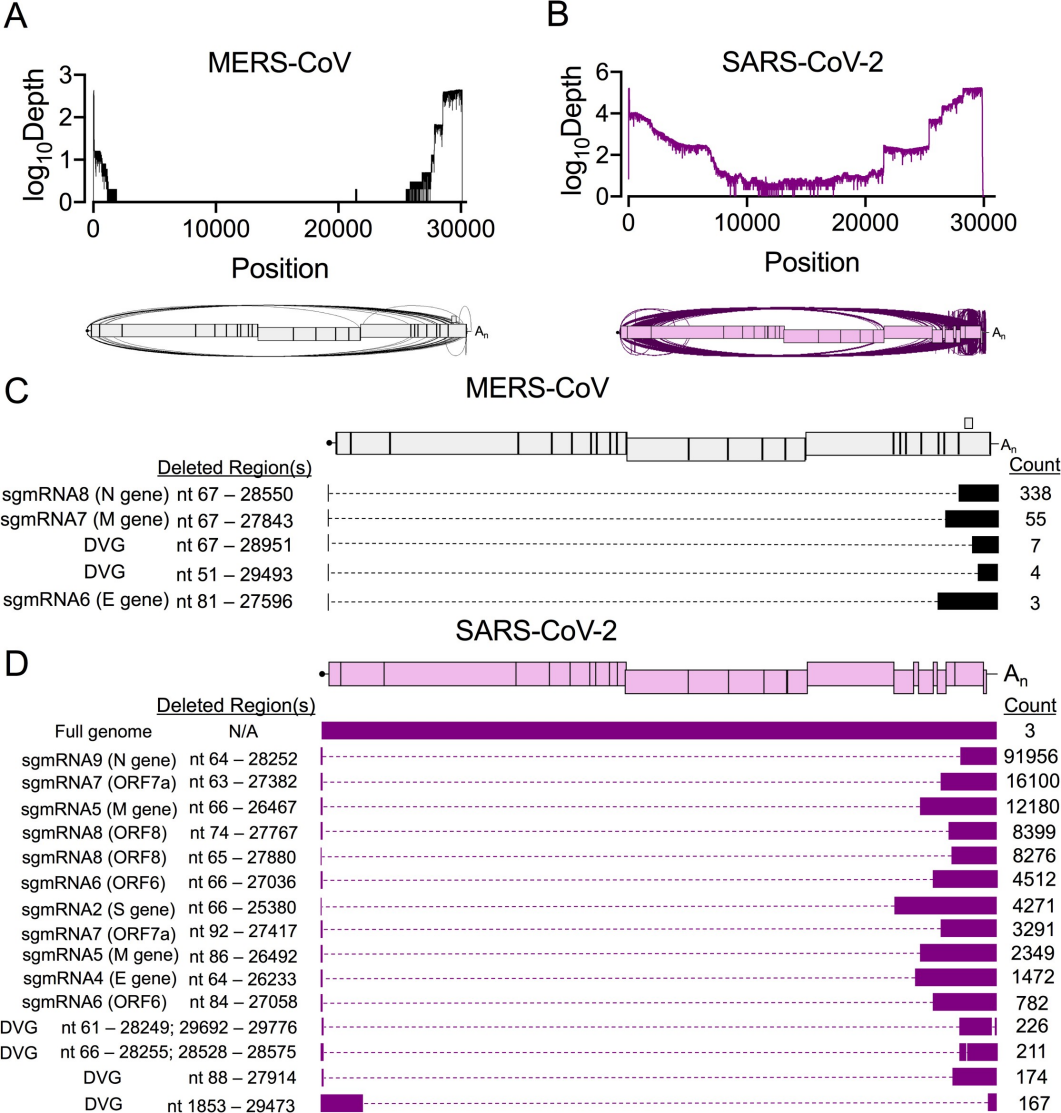
Direct RNA Nanopore sequencing of MERS-CoV and SARS-CoV-2 reveals accumulation of distinct recombined RNA populations. Direct RNA Nanopore sequencing of poly-adenylated MERS-CoV and SARS-CoV-2 RNA. Three sequencing experiments were performed for each virus. Nanopore reads passing quality control were combined and mapped to the viral genome using *minimap2* [70]. Genome coverage maps and Sashimi plots visualizing junctions (arcs) in full-length (A) MERS-CoV (black) and (B) SARS-CoV-2 (violet) RNA reads. (C) Distinct RNA molecules identified in MERS-CoV (black) with at least 3 supporting reads are visualized. The number of sequenced reads containing the junction is listed (Count). Genetic sequences of each RNA molecule are represented by filled boxes and deleted regions are noted (Deleted Region(s)) and represented by dashed lines. (D) The 15 most abundant SARS-CoV-2 (violet) recombined RNA molecules and 3 full-genome reads are visualized. See also S2 Table, S3 Table, S4 Table.

We performed direct RNA Nanopore sequencing on the same RNA used for short-read RNA-seq. We analyzed three independent experiments for each virus and sequenced 178,658 MERS-CoV RNA molecules and 1,725,862 SARS-CoV-2 RNA molecules that had 85.6% and 82.2% identity to the parental genome, respectively (S2 Table). To remove prematurely truncated sequences, we computationally selected only Nanopore reads containing both genomic termini. We obtained 3 full-length direct RNA sequences of the SARS-CoV-2 genome containing over 29,850 consecutive nucleotides that aligned to the SARS-CoV-2 genome (S3 Table). In MERS-CoV RNA, we detected 451 full-length molecules containing genomic termini and 473 unique junctions (Fig 2A and S2 and S4 Tables). SARS-CoV-2 RNA generated 172,191 complete molecules and 181,770 unique junctions (Fig 2B and S2 and S4 Tables). To confirm junctions in detected by direct RNA sequencing, we compared unique junctions detected in filtered complete RNA molecules with 20 bp windows at both the start and stop sites to unique junctions detected in short-read Illumina RNA-seq datasets reported in Figs 1 and S2. 89.29% of MERS-CoV and 97.97% of SARS-CoV-2 Nanopore junctions were also detected in RNA-seq datasets S2 Table).

To define the architectures of detected molecules, we filtered for junctions with at least 3 supporting Nanopore reads. For both viruses, junctions were categorized as either a DVG or sgmRNA junction using the same criteria as with the RNA-seq data. In MERS-CoV, we defined 5 distinct species, including 3 sgmRNAs (6, 7, and 8) and 2 DVGs (Fig 2C). In SARS-CoV-2, there were 1166 species with a single junction and 227 containing 2 junctions. The 15 most abundant species in SARS-CoV-2 included 11 predicted sgmRNA transcripts and 4 DVGs (Fig 2D). We also identified potential alternative transcripts corresponding to the ORF6, ORF7a, ORF8, and the M genes (Fig 2D). In summary, direct RNA Nanopore sequencing defined a diverse set of recombined RNAs generated by both MERS-CoV and SARS-CoV-2 with most DVGs containing only a singular recombination event rather than extensive genomic rearrangement. Thus, both MERS-CoV and SARS-CoV-2 engaged in extensive RNA recombination during replication, producing diverse junctions across the viral genomes and many recombined RNA species.

### Genetic inactivation of the MHV nsp14-exoribonuclease (ExoN) resulted in significantly decreased and altered RNA recombination

We previously have reported that the nsp14 exoribonuclease (nsp14-ExoN) activity is required for high-fidelity replication and proofreading for the β-CoVs murine hepatitis virus (MHV) and SARS-CoV [33–36]. We sought to determine whether nsp14-ExoN activity also contributed to the extensive recombination observed in coronaviruses. Since no proofreading-deficient nsp14-ExoN catalytic mutant is available for MERS-CoV or SARS-CoV-2, we used the MHV nsp14-ExoN inactivation mutant (MHV-ExoN(-)) and wild-type virus (MHV-WT) to compare recombination [49]. Murine DBT cells were infected with MHV-WT or MHV-ExoN(-) in three independent experiments, and RNA was isolated from infected cell monolayers and viral supernatant when the cell monolayer was intact and 90% cytopathic effect (CPE) was observed. Poly(A)-selected RNA-seq libraries were aligned to the MHV genome using *ViReMa* (AY910861.1). In both infected cell monolayers and viral supernatants, MHV-WT and MHV-ExoN(-) had similar mean coverages ranging between 1100 and 1700 reads (S4A and S4B Fig).

**Fig 3:**
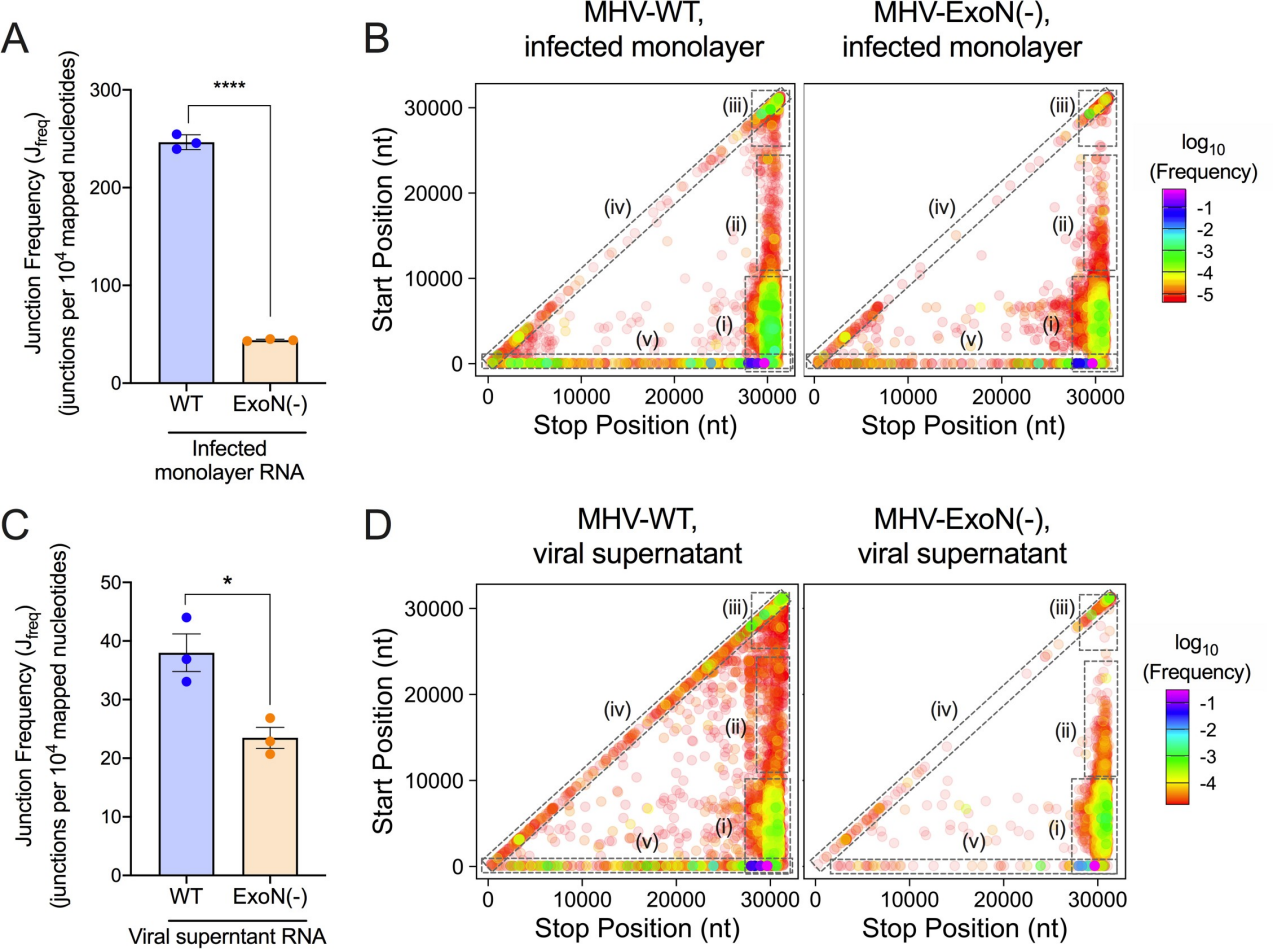
Loss of nsp14-ExoN activity decreases recombination frequency and alters recombination junction patterns across the genome. Infected monolayer and viral supernatant RNA poly(A) selected, sequenced by RNA-seq, and aligned to the MHV genome using *ViReMa*. Junction frequency (J_freq_) in infected monolayer RNA (A) and viral supernatant RNA (C) was calculated by normalizing the number of nucleotides in *ViReMa-*detected junctions to total viral RNA (total mapped nucleotides) and multiplying by 10,000, expressing J_freq_ as number of junctions per 10^4^ mapped nucleotides. Error bars represent standard error of the means (SEM) (N = 3). Statistical significance was determined by the unpaired student’s t-test. * p < 0.05, **** p < 0.0001. Unique forward (5’ ➔ 3’) recombination junctions detected in infected monolayers (C) and viral supernatant (E) were mapped in MHV-WT and MHV-ExoN(-) according to their genomic position. Junctions are colored according to their frequency in the population (high frequency = magenta; low frequency = red). Clusters are marked by dashed boxes: (i) 5’ ➔ 3’; (ii) mid-genome ➔ 3’; (iii) 3’ ➔ 3’; (iv) local deletions; (v) 5’ UTR ➔ rest of genome. See also S3 and S4 Figs.

Previous studies have shown that MHV-ExoN(-) has decreased genome replication compared to WT [34]. We accounted for decreased MHV-ExoN(-) viral RNA by normalizing the number of nucleotides participating in detected junctions to the amount of viral RNA (total mapped nucleotides), and J_freq_ was calculated as described for Fig 1A. MHV-ExoN(-) had significantly decreased J_freq_ relative to MHV-WT in both infected cells and viral supernatant (Fig 3A and 3C). To address any potential viral replication bias resulting from the differences between MHV-WT and MHV-ExoN(-) replication that have been previously reported, we quantified and compared the unique detected recombination junctions. In both infected cell monolayers and in viral supernatant, MHV-ExoN(-) had significantly decreased unique recombination junctions compared to MHV-WT (S3C and S4C Figs). Thus, MHV-ExoN(-) had decreased recombination junction frequency and number of unique junctions compared to MHV-WT, showing that loss of nsp14-ExoN activity resulted in significantly less recombination during infection.

Recombination junctions were plotted according to their start (5’) and stop (3’) sites in infected cells and viral supernatant (Figs 3B, 3D, S3C, S3D, S4C and S4D). MHV-WT displayed clusters of junctions that were similar to those demonstrated in MERS-CoV and SARS-CoV-2, specifically: 1) between the 5’ and 3’ ends of the genome; 2) between intermediate genomic positions and the 3’ end of the genome; 3) between the 5’ UTR and the rest of the genome; 4) in local deletions across the genome; and 5) within the 3’ end of the genome (Fig 3B and 3D). While both WT and MHV-ExoN(-) accumulated junction clusters between the 5’ and 3’ ends of the genome and within the 3’ end of the genome, MHV-ExoN(-) had fewer junctions between the 5’ UTR and the rest of the genome and fewer junctions forming local deletions (Fig 3B and 3D). Thus, loss of MHV nsp14-ExoN activity resulted in decreased recombination frequency and altered junction patterns across the genome.

### MHV-ExoN(-) had altered recombination at distinct positions across the genome

**Fig 4:**
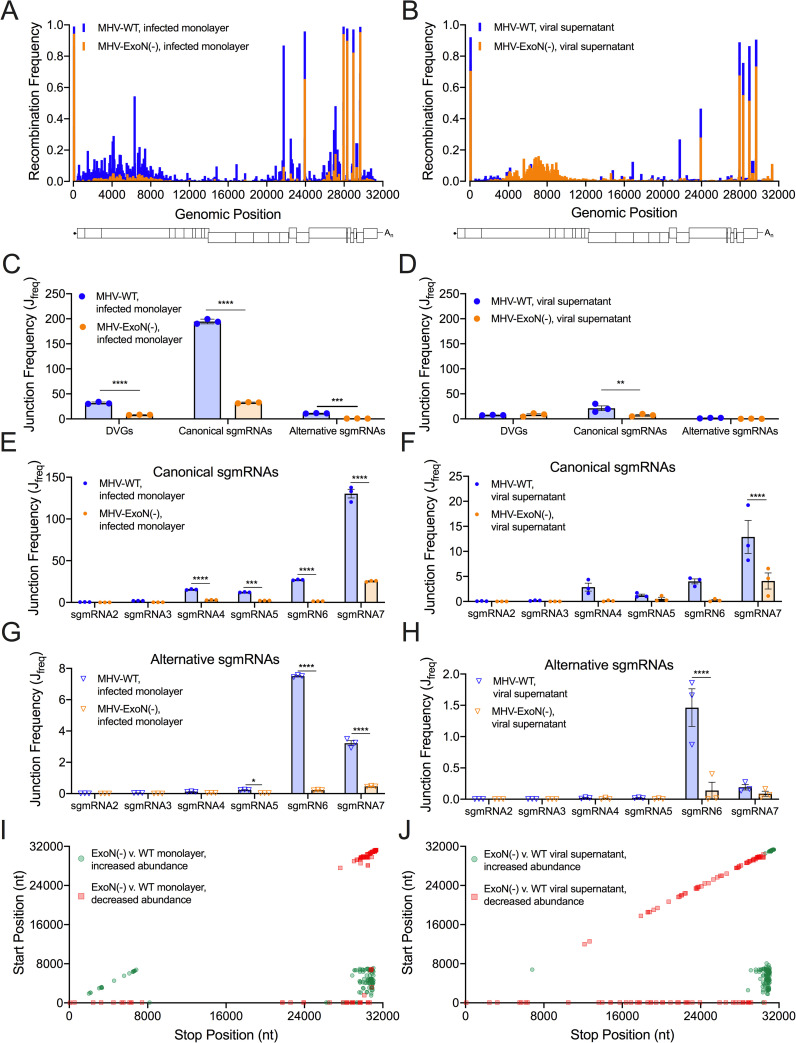
Loss of nsp14-ExoN alters recombination at multiple genomic loci and skews recombined RNA populations. Mean recombination frequency at each position across the MHV genome was compared in MHV-WT (blue) and MHV-ExoN(-) (orange) infected monolayer (A) and viral supernatant RNA (B). 2-way ANOVA with multiple comparisons (N = 3). The junction frequencies (J_freq_) of DVGs, canonical sgmRNAs, and alternative sgmRNAs were compared in MHV-WT (blue) and MHV-ExoN(-) (orange) infected monolayers (C) and viral supernatant (D). Error bars represent standard errors of the mean (SEM) (N = 3) and statistical significance was determined by a 2-way ANOVA with multiple comparisons correct by statistical hypothesis testing (Sidak test), ** p <0.01, *** p < 0.001, **** p < 0.0001. The J_freq_ of canonical sgmRNA junctions was compared in MHV-WT (blue) and MHV-ExoN(-) (orange) infected monolayers (E) and viral supernatant (F). Error bars represent SEM (N = 3). Statistical significance was determined by a 2-way ANOVA with multiple comparisons corrected by statistical hypothesis testing (Sidak test), *** p < 0.001, **** p < 0.0001. The J_freq_ of alternative sgmRNA molecules was quantified for MHV-WT (blue) and MHV-ExoN(-) (orange) infected cell monolayers (G) and viral supernatant (H). Error bars represent SEM (N = 3). Statistical significance was determined by a 2-way ANOVA with multiple comparisons corrected by statistical hypothesis testing (Sidak test), * p < 0.05, **** p < 0.0001. The abundance of junctions in MHV-ExoN(-) was compared to MHV-WT in infected monolayers (I) and viral supernatant (J) by *DESeq2*. Junctions with statistically significant altered abundance (p < 0.05, N = 3) in MHV-ExoN(-) are mapped across the genome and colored according to their fold-change (red squares = decreased abundance, green circles = increased abundance). See also S3–S5 Figs and S5 and S6 Tables.

We next calculated and compared mean recombination frequency at each genomic position in MHV-WT and MHV-ExoN(-) (Fig 4A–4B). Both MHV-WT and MHV-ExoN(-) had high recombination frequency at the 5’ and 3’ ends of the genome as well as at distinct sites across the genome. Positions with >50% recombination frequency were localized to the TRS regions (Fig 4A and 4B). MHV-ExoN(-) had significantly altered recombination frequency at 765 positions in infected cell RNA and 499 positions in viral supernatant RNA (Figs 4A and 4B and S5). These positions were distributed across the genome, including the 5’ TRS-Leader, non-structural protein coding sequences, TRSs, structural and accessory ORFs, and 3’ UTR (S5A–S5E Fig). Thus, genetic inactivation of nsp14-ExoN altered recombination frequency at multiple positions across the genome.

### MHV-ExoN(-) had decreased abundance and altered ratios of DVGs and sgmRNAs

Compared with WT, MHV-ExoN(-) had significantly decreased frequencies of DVGs and both canonical and alternative sgmRNAs (Fig 4C). MHV-ExoN(-) viral supernatant also demonstrated a significant decrease in canonical sgmRNAs (Fig 4D). In addition to frequencies of DVGs and sgmRNAs in MHV-ExoN(-), the ratios of DVGs and both canonical and alternative sgmRNAs were skewed. Compared to WT, MHV-ExoN(-) had a significantly increased proportion of DVGs and significantly decreased proportions of both canonical and alternative sgmRNAs (S3E and S4E Figs). MHV-ExoN(-) also displayed significantly skewed proportions of individual canonical and alternative sgmRNA species (S3F and S3G Fig and S4F and S4G Fig). Decreased frequencies and aberrant proportions of DVGs and both canonical and alternative sgmRNAs show that nsp14-ExoN activity is a key determinant in recombination producing distinct RNA populations.

### MHV-ExoN(-) had altered junction site selection

We next identified junctions with altered abundances in MHV-ExoN(-) compared to MHV-WT using *DESeq2* [50]. MHV-ExoN(-) generated recombination junctions with significantly increased or decreased abundance relative to MHV-WT (S5F and S5G Fig and S6 Table). Clusters of junctions with either increased or decreased abundance in MHV-ExoN(-) compared to WT were localized to distinct genomic regions. Recombination junctions significantly enriched in MHV-ExoN(-) were mainly found between the 5’ and 3’ ends of the genome (Fig 4I and 4J). Junctions with significantly decreased abundance in MHV-ExoN(-) clustered between the 5’ UTR and the rest of the genome and local deletions of 10–50 bp in length across the genome (Fig 4I and 4J). Thus, the populations of recombination junctions that were differentially abundant in MHV-ExoN(-) were not randomly distributed across the genome, suggesting specific changes to junction site selection.

### MHV-ExoN(-) DVG junction-flanking sequences demonstrated altered nucleotide composition while retaining microhomology at junction sites

**Fig 5:**
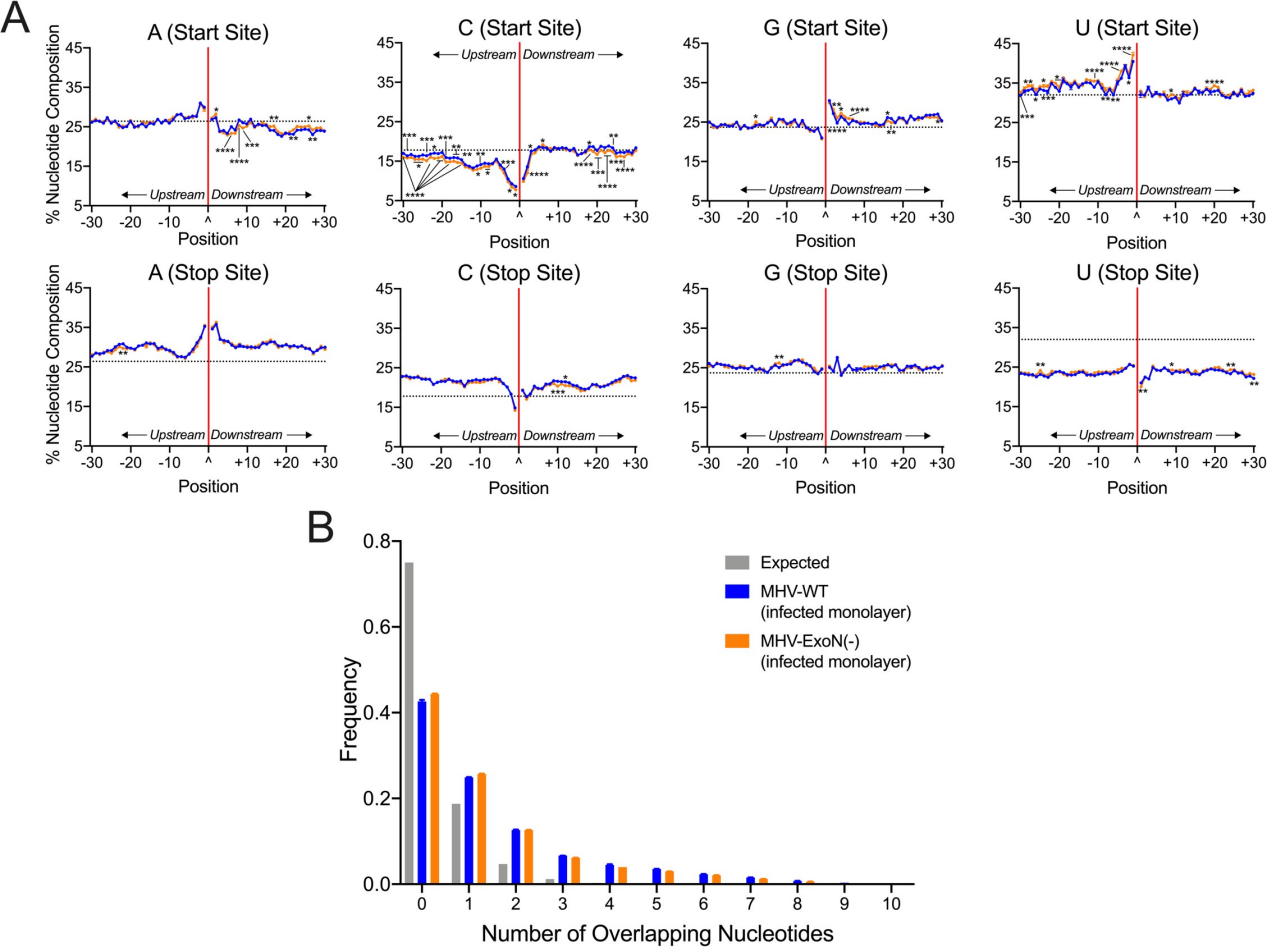
MHV-ExoN(-) DVG junction sites display both WT-like patterns of sequence composition and multiple alterations in nucleotide frequency, revealing microhomology at junctions. (A) Nucleotide composition was calculated as the percent adenosine (A), cytosine (C), guanine (G), and uracil (U) at each position in a 30-base pair region flanking DVG junction start and stop sites in MHV-WT (blue) and MHV-ExoN(-) (orange) infected monolayer RNA. The junction is labelled as a carat (^) and a solid red line with upstream positions numbered -30 to -1 and downstream positions +1 to +30. The expected nucleotide percentage was calculated based on the overall MHV genome and represented as a dashed black line. Each point represents a mean (N = 3) and error bars represent SEM. 2-way ANOVA with multiple comparisons corrected for false discovery rate (FDR) by the Benjamini-Hochberg method. * q < 0.05, ** q < 0.01, *** q < 0.001, **** q < 0.0001. (B) Distribution of microhomology overlaps in MHV-WT (blue) and MHV-ExoN(-) (orange) compared to an expected probability distribution (gray). The frequency of each overlap length is displayed as a mean (N = 3) and error bars represent SEM. See also S5 Fig.

To test whether MHV-ExoN(-) has altered sequence composition at its recombination junctions, we filtered DVG junctions and quantified nucleotide composition of adenosine (A), cytosine (C), guanine (G), and uracil (U) in the start and stop sequences flanking junction sites. Both MHV-WT and MHV-ExoN(-) demonstrated similar patterns of depletion and enrichment of nucleotides in infected cell monolayers and viral supernatant (Figs 5A and S6A). Start site sequences favored sequences of UUU(U/A)(U/A)^GG and were depleted for C upstream of the junction. Stop site sequences were relatively enriched for the sequence AAA(U/A)(U/A)^AA(G/A). These patterns and sequence preferences were similar to the sequence composition of both MERS-CoV and SARS-CoV-2 DVG recombination junctions (Fig 1F). In all three viruses, a preference for UUG spanning junction start sites was defined. Further, the DVG junction sequence preference differed from sequence composition of TRS-like sequences for MHV (AAUCUAUAC) [51] and represented a different selection of sequences for DVG formation. Loss of nsp14-ExoN(-) activity resulted in significantly altered nucleotide composition at multiple positions for all nucleotides in both the start and stop sites (Figs 5A and S6A). For both MHV-WT and MHV-ExoN(-), junction sites encoded more and longer microhomology overlaps of up to 8bp than would be expected by chance (Figs 5B and S6B).Thus, while loss of nsp14-ExoN activity altered nucleotide composition at multiple positions surrounding DVG junction sites, the overall patterns of enrichment and depletion were maintained and microhomology at the junction sites remained unchanged.

### Direct RNA Nanopore sequencing identified changes in MHV-ExoN(-) full-length recombined RNA populations

**Fig 6:**
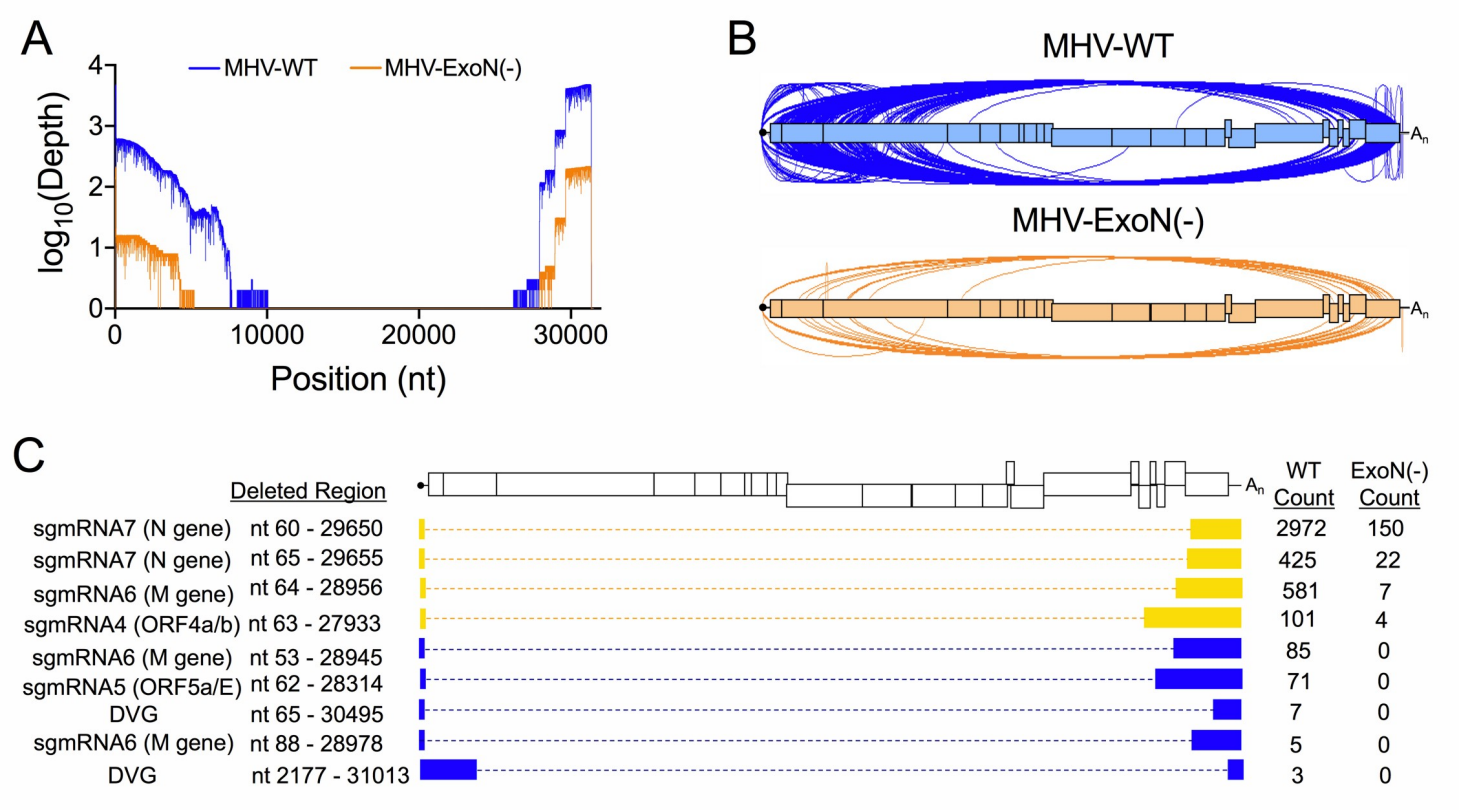
Direct RNA Nanopore sequencing of MHV full-length recombined RNA molecules. Direct RNA Nanopore sequencing of MHV viral supernatant RNA. (A) Genome coverage maps of full-length MHV-WT (blue) and MHV-ExoN(-) (orange) Nanopore reads aligned to the MHV-A59 genome using *minimap2*. (B) Sashimi plot visualizing junctions (arcs) in MHV-WT (blue) and MHV-ExoN(-) (orange). (C) RNA molecule genetic architectures with at least 3 supporting reads identified in both MHV-WT and MHV-ExoN(-) (yellow) and unique to MHV-WT (blue). Genetic sequences of the RNA molecule are represented by filled boxes. Deleted regions are reported (Deleted Region) and represented by dashed lined. The number of reads supporting each species are noted (Count). See also S2 Table, S3 Table, and S4 Table.

To test the alterations of recombined RNAs due to loss of nsp14-ExoN proofreading activity, we sequenced MHV-WT and MHV-ExoN(-) viral supernatant RNA by direct RNA Nanopore sequencing. When reads were mapped to the MHV genome using *minimap2*, MHV-WT datasets contained 102,367 viral molecules and MHV-ExoN(-) contained 19,445 (Fig 6A and S2 Table). We validated MHV-WT and MHV-ExoN(-) Nanopore junctions by comparing to RNA-seq datasets. 96.00% of MHV-WT and 97.50% of MHV-ExoN(-) Nanopore junctions were also detected in RNA-seq datasets (S2 Table).

MHV-ExoN(-) had a global decrease in the number of junctions across the genome (Fig 6B and S2 and S4 Tables). We filtered MHV-WT and MHV-ExoN(-) datasets for RNA molecules containing both 5’ and 3’ genomic ends that were supported by at least three reads. Nine such architectures were identified in MHV-WT (Fig 6C). These populations contained both DVGs and sgmRNAs. The four most abundant species were also detected in MHV-ExoN(-) viral supernatant RNA, which corresponded to a DVG and sgmRNAs 4,6 and 7 (Fig 6C). We did not detect unique MHV-ExoN(-) variants with at least 3 supporting reads, potentially due to their low frequency in the population. These data demonstrate that loss of nsp14-ExoN activity drives the accumulation altered recombined RNA populations and skewed DVG species diversity.

## Discussion

While CoV recombination has long been proposed as a driver of novel strain emergence and is known to be a constitutive aspect of CoV replication, the diversity of recombination products and sequence and protein determinants had not previously been defined. In this study, we show the diversity of the CoV recombination landscape in the β-coronaviruses SARS-CoV-2, MERS-CoV, and murine hepatitis virus (MHV), and we demonstrate that loss of the nsp14 exoribonuclease activity in MHV results in decreased recombination and altered site selection of recombination junctions. Our results support a model in which nsp14-ExoN activity is required for normal recombination. Thus, nsp14-ExoN is a key component of CoV recombination, adding another essential function to the repertoire of those already reported for nsp14-ExoN, specifically CoV high-fidelity replication, RNA synthesis, resistance to antiviral nucleoside analogues, fitness, immune antagonism, and virulence.

### Divergent β-CoVs generate extensive and similar recombination networks yielding diverse populations of RNA species

We show that MHV, MERS-CoV, and SARS-CoV-2 perform extensive recombination and generate diverse populations of RNA molecules, demonstrated by independent short-read Illumina RNA-seq and long-read, direct RNA Nanopore sequencing. These divergent group 2a (MHV), 2b (SARS-CoV-2), and 2c (MERS-CoV) β-CoVs demonstrated many strong similarities in their patterns of recombination junctions across the genomes and in the types of recombined RNAs produced. Specifically, the similarities across all three viruses in the nucleotide composition of sequences flanking DVG junctions and the common increased junction sequence microhomology support the conclusion that recombination mechanisms have been conserved across different evolutionary trajectories and host species specificity.

There also were distinct recombination patterns for each virus that were confirmed across independent experiments and by agreement between RNA-seq and Nanopore datasets for all viruses. These differences most likely represent evolutionary divergence of recombination in distinct viruses or sub-genera represented by MHV, SARS-CoV-2 and MERS-CoV. However, it remains possible that observed differences could be impacted by the diversity of the original sample or replication in different cell types. SARS-CoV-2 stock virus was a low passage (P5) population from a clinical isolate that had been passaged in Vero cells, while MERS-CoV and MHV were low passage stocks generated from isogenic cDNA clones. It will be important for future studies to determine the role of the diversity of the viral population, cell environment, virus-specific RNA synthesis kinetics, and virus adaptation/evolution in viral recombination. The extent of the pandemic and availability of genetically diverse viruses will allow investigators to test whether patterns of SARS-CoV-2 recombination show alterations between early and later pandemic isolates, and if any identified differences correlate with or predict changes in other replication or pathogenesis.

### Sequences containing microhomology are likely determinants of recombination resulting in CoV defective viral genome formation

High-resolution analysis of DVG junctions produced during replication by MERS-CoV, SARS-CoV-2, and MHV reveals that a significant preference for a UUG motif, suggesting a possible conserved core sequence for DVG synthesis that differs from sgmRNA transcriptional regulatory sequences. These results support a model across multiple divergent β-CoVs in which DVGs result from recombination junction selection by the RTC based on both broadly similar sequence identity and specific sequence microhomology of 2–10 bp (S1D Fig). This model would be most similar to microhomology-mediated end-joining, a mechanism of genomic repair in eukaryotic DNA systems that results in large sequence deletions [52,53]. The presence of sequence homology-driven recombination and DVG formation suggests an selection for specific DVG biogenesis, supporting the hypothesis that DVGs play specific roles in coronavirus replication, pathogenesis and evolution. The results of this study will form the basis for direct genetic studies of DVGs as well as ability to target templates for study of the viral replicase functions.

### MHV nsp14-ExoN determines the extent, diversity, and junction site selection of RNA recombination during infection

MHV-ExoN(-) mutants showed decreased recombination junction frequency and altered populations of sgmRNAs and DVGs, demonstrating a previously unknown role for nsp14-ExoN in CoV RNA recombination. There is no precedent in RNA viruses for the regulation of recombination by a virus encoded exoribonuclease. In contrast, in DNA viruses such as poxviruses and herpesviruses, virus-encoded exonuclease activity stimulates recombination by single-strand annealing through both exonuclease degradation of nucleic acids and interactions with other proteins [39,40]. In the single-stranded, positive-sense RNA virus families *picornaviridae* and *alphaviridae* that lack any exonuclease, low-fidelity mutant viruses have altered polymerase speed and processivity [54] and these properties contribute to recombination and the generation of DVGs [32,55,56]. Our results suggest that CoVs have evolved to regulate both proofreading and recombination by the nsp14-ExoN protein. Mutation of the active site of nsp14-ExoN alters both these functions, supporting a complex interaction with other proteins in the CoV RTC, including the nsp12 RNA-dependent RNA polymerase. In the low-fidelity picornavirus and alphavirus mutants, it has been proposed that impaired fidelity alters polymerase processivity and speed, resulting in decreased recombination. It is possible that CoV nsp14-ExoN mutations may similarly impair polymerase speed and processivity, resulting in altered patterns of DVGs and non-canonical sgmRNAs. The direct role of polymerase speed and processivity and the potential mechanisms by which these principles influence recombination remains to be determined, but possibilities include altered RTC stability through the changes to the complex protein-protein interactions or RTC-RNA interactions.

### ExoN is a powerful tool for understanding CoV replication, and a novel and conserved target for inhibition and attenuation

The similarities between the patterns of recombination across divergent WT β-CoVs, along with the differences observed between recombination in MHV WT and ExoN(-) viruses, support the hypothesis that ExoN mutants will inform our understanding of the evolution of the unique CoV multi-protein polymerase complex. Specifically, the model of DVG synthesis defined in MHV, MERS-CoV, and SARS-CoV-2 will allow for the direct testing of the roles of DVGs in CoV replication. Further, the role of ExoN in CoV recombination, along with the previously defined roles of ExoN in RNA proofreading during replication, native resistance to nucleoside analogues, immune evasion, and virulence and pathogenesis, highlight nsp14-ExoN as conserved and vulnerable target for both antiviral inhibitors and virus attenuation. ExoN(-) viruses are profoundly more sensitive to a range of antiviral nucleoside analogues, including remdesivir, ribavirin, 5-fluorouracil, and β-d-N^4^-hydroxycytidine (NHC, EIDD 1931/2801) [33,38,57]. Nucleoside analogues and exonuclease inhibitors that target nsp14-ExoN can be tested for an additional impact on recombination and illuminate antiviral mechanisms of action. Finally, recombination has driven the vaccine escape in multiple CoVs [11,12]. The finding that MHV-ExoN(-) has decreased recombination during viral replication may have important implications for any design of live-attenuated SARS-CoV-2 or other animal or zoonotic CoVs. Our previous studies have shown that the ExoN(-) substitutions in MHV and SARS-CoV are evolutionarily stable over long-term passage in culture and in mice, and that a SARS-CoV ExoN(-) mutant is attenuated in mice while producing a robust and protective immune response against WT SARS-CoV infection [38,42,58,59]. The results in this paper raise the intriguing possibility that any CoV encoding ExoN(-) would have less recombination potential for repair or escape.

## Materials and methods

### Cell lines

DBT-9 (delayed brain tumor, murine astrocytoma clone 9) cells were maintained at 37°C as described previously [60]. DBT-9 cells were originally obtained from Ralph Baric at University of North Carolina-Chapel Hill and were maintained within 50 passages of this progenitor stock. Cells were maintained in Dulbecco’s modified Eagle medium (DMEM) (Gibco) supplemented with 10% fetal clone serum (FCS) (Invitrogen), 100 U/mL penicillin and streptomycin (Gibco), and 0.25 μg/mL amphotericin B (Corning). *Cercopithecus aethiops* Vero CCL-81 cells maintained in Dulbecco’s modified Eagle medium (DMEM) (Gibco) supplemented to final concentrations of 10% fetal calf serum (Gibco), 100 IU/ml penicillin (Mediatech), 100 mg/ml streptomycin (Mediatech), and 0.25 mg/ml amphotericin B (Mediatech) were used for MERS-CoV-2 infection. Vero CCL-81 cells were obtained from ATCC. Vero E6 cells maintained in Dulbecco’s modified Eagle medium (DMEM) (Gibco) supplemented to final concentrations of 10% fetal calf serum (Gibco), 100 IU/ml penicillin (Mediatech), 100 mg/ml streptomycin (Mediatech), and 0.25 mg/ml amphotericin B (Mediatech) were used for SARS-CoV-2 infections. Vero E6 cells were obtained from ATCC.

### Viruses

All MHV work was performed using the recombinant WT strain MHV-A59 (GenBank accession number AY910861.1 [61]) at passage 4 and an engineered ExoN(-) strain of MHV-A59 at passage 2. The recovery of MHV-ExoN(-) were previously described include the four-nucleotide substitution of motif I residues resulting in alanine substitution (DE ➔ AA) [34] Experiments involving MERS-CoV were conducted using the human EMC/2012 strain recovered from an infectious clone (GenBank accession number JX869059.2) [62]. Experiments involving SARS-CoV-2 were conducted with a passage 5 virus inoculum generated from a Seattle, WA, USA COVID-19 patient (GenBank accession number MT020881.1). All virus manipulations were performed under stringent BSL-3 laboratory conditions according to strict protocols designed for safe and controlled handling of MERS-CoV and SARS-CoV-2.

### MHV isolation and viral supernatant purification

Subconfluent 150-cm^2^ flasks were infected with either MHV-A59 or MHV-ExoN(-) at an MOI of 0.01 PFU/cell. Supernatant was harvested at either 16 hours post infection (MHV-A59) or 24 hours post infection (MHV-ExoN(-)) when the monolayer was >95% fused and remained intact. Infection supernatant was clarified by centrifugation at 1500 x *g* for 5 minutes at 4°C. Viral supernatant was purified on a 30% sucrose cushion by ultracentrifugation at 25,000 RPM at 4°C for 16 hours. The viral pellet was resuspended in MSE buffer (10mM MOPS, pH 6.8; 150mM NaCl; 1 mM EDTA). Viral RNA was extracted using the TRIzol-LS reagent according to manufacturer’s protocols. RNA was quantified using the Qubit RNA HS assay. Supernatant data in this paper is the result of three experiments sequenced independently from the infected cell monolayer samples.

### MHV isolation from infected monolayers

In three independent experiments, a subconfluent 150-cm^2^ flask of DBT-9 cells was infected with either MHV-WT or MHV-ExoN(-) at an MOI or 0.01 PFU/cell. Monolayer was harvested at either 16 hpi (MHV-WT) or 24 hpi (MHV-ExoN(-)) when the monolayer was >95% fused and >75% intact. RNA was extracted with TRIzol according to manufacturer’s protocols. Infected monolayer data in this paper is the result of three independent experiments sequenced independently.

### MERS-CoV infection

In three independent experiments, a nearly confluent 25-cm^2^ flask of Vero CCL-81 cells was infected with MERS-CoV at an MOI of 0.3 pfu/cell. Total infected cell lysates were collected at 72 hpi with the monolayer >70% fused. RNA was extracted in TRIzol according to manufacturer’s protocols.

### SARS-CoV-2 infection

In three independent experiments, a total of 5 subconfluent 25-cm^2^ flasks of Vero E6 cells were infected at an MOI = 0.45 pfu/cell and cellular monolayers were harvested 60 hpi when the monolayer was >90% fused. RNA was extracted in TRIzol according to manufacturer’s protocols.

### Short-read Illumina RNA-sequencing of viral RNA

Next generation sequencing (NGS) libraries were generated using 2 μg of RNA of each sample. RNA was submitted to Genewiz for library preparation and sequencing. Briefly, after quality control, polyadenylated RNA was selected during library preparation. Isolated RNA was heat fragmented, RT-PCR amplified with equivalent number of cycles, size-selected, and libraries were prepared for 2 x 150 nucleotide paired-end sequencing performed (Illumina). Genewiz performed basecalling and read demultiplexing.

### Direct RNA Nanopore sequencing

RNA from ultracentrifuge-purified viral supernatant was prepared for direct RNA Nanopore sequencing on the Oxford Nanopore Technologies MinION platform according to the manufacturer’s protocols. Libraries were sequenced on fresh MinION R9.4 flow-cells for 24 hours, or until the pore occupancy was under 20%. Viral supernatant RNA from three independent experiments was sequenced on three separate flow cells for both MHV-WT and MHV-ExoN(-). MERS-CoV RNA from three independent cultures was sequenced on three separate flow cells. SARS-CoV-2 RNA isolated from three independent infections was sequenced on three separate flow cells.

### Illumina RNA-seq processing and alignment

Raw reads were processed by first removing the Illumina TruSeq adapter using *Trimmomatic* [63] default settings (command line parameters java -jar trimmomatic.jar PE sample_R1.fastq.gz sample_R2.fastq.gz output_paired_R1.fastq output_unpaired_R1.fastq output_paired_R2.fastq output_unpaired_R2_unpaired.fastq ILLUMINACLIP:TruSeq3-PE.fa:2:30:10 LEADING:3 TRAILING:3 SLIDINGIWINDOW:4:15 MINLEN:36). Reads shorter than 36 bp were removed and low-quality bases (Q score < 30) were trimmed from read ends. The raw FASTQ files were aligned to the MHV-A59 genome (AY910861.1), the MERS-CoV genome (JX869059.2), and the SARS-CoV-2 genome (MT020881.1) using the Python2 script *ViReMa* (Viral Recombination Mapper, version 0.15) [44] using the command line parameters python2 ViReMa.py reference_index input.fastq output.sam—OuputDir sample_virema/—OutputTag sample_virema -BED—MicroIndelLength 5. The sequence alignment map (SAM) file was processed using the samtools [64] suite to calculate nucleotide depth at each position in a sorted binary alignment map (BAM) file (using command line parameters samtools depth -a -m 0 sample_virema.sorted.bam > sample_virema.coverage).

### Recombination junction analysis

Recombination junction frequency (J_freq_) was calculated by comparing the number of nucleotides in detected recombination junctions to the total number of mapped nucleotides in a library. Nucleotides in detected recombination junctions were quantified as a sum of nucleotide depth reported at each junction in the BED file generated by *ViReMa*. Total nucleotides mapped to the MHV-A59 genome were quantified as a sum of nucleotide depth at each position across the genome in the tab-delineated text file generated by the *samtools*. J_freq_ was reported as junctions per 10^4^ nucleotides sequenced. Mean J_freq_ values were compared between MHV-WT and MHV-ExoN(-) and statistical significance determined by an unpaired student’s t-test. Junctions were mapped across the genome according to their start (5’) and stop (3’) positions. These junctions were first filtered in the forward (5’ ➔ 3’) direction using the *dpylr* package (RStudio). The frequency of each junction was calculated by comparing the depth of the unique junction to the total number of nucleotides in all detected junctions in a library. Junctions were plotted according to the genomic position and colored according to log_10_ of the frequency using *ggplot2* in RStudio.

Recombination frequency was calculated at each genomic position by dividing the number of nucleotides in any junction mapping to the position divided by the total number of nucleotides sequenced at the position. Mean recombination frequencies were compared between MHV-WT and MHV-ExoN(-) for three independent sequencing experiments by a 2-way ANOVA statistical analysis with multiple comparisons corrected through statistical hypothesis testing using the Sidak test.

### Identification of sgmRNA and DVG junctions

Forward recombination junctions were classified as either canonical sgmRNA junctions, alternative sgmRNA junctions or DVG junctions based on the position of their junction sites and filtered in Microsoft Excel. Briefly, junction start sites were filtered to those positioned within 30 nucleotides of the TRS-L for each virus. The stop sites were then filtered for those positioned within 30 nucleotides of each respective sgmRNA TRS. This window is supported by other reports defining the flexibility of the CoV transcriptome [45,65]. Canonical sgmRNAs were identified as the most abundant junction matching these criteria. Other, less abundant sgmRNA junctions were categorized as alternative sgmRNAs. The junction frequency (J_freq_) of each sgmRNA was calculated by dividing the number of nucleotides in a specific sgmRNA population by the total amount of viral RNA (total mapped nucleotides). This ratio is multiplied by 10,000 to scale for the number of nucleotides sequenced and is therefore expressed as the number of junctions per 10^4^ mapped nucleotides. The filtered sgmRNA junctions were compiled and DVG junctions were filtered in RStudio by performing an exclusionary anti_join() using *dplyr* on forward junctions identified in each sample. DVG J_freq_ was calculated by dividing the number of nucleotides in DVG junctions by the total amount of viral RNA in a sample (total mapped nucleotides). The ratio is multiplied by 10,000 to scale for number of nucleotides sequenced and the frequency is expressed as the number of junctions per 10^4^ mapped nucleotides. The percentage of canonical and alternative sgmRNA and DVG junctions was calculated by comparing the depth of all filtered sgmRNA or DVG junctions to the sum of all detected forward junctions. Mean percent canonical and alternative sgmRNAs and DVG was compared between three independent sequencing experiments in viral supernatant RNA. Statistical significance was determined by a 2-way ANOVA test with multiple comparisons and corrected by statistical hypothesis testing using the Sidak test.

### Differential abundance of junctions

To compare the abundance of junctions in MHV-A59 and MHV-ExoN(-), the ViReMa output list of junctions was analyzed by in-house scripts (https://github.com/DenisonLabVU) and the R package *DESeq2* [50]. Junctions significantly up- or down-regulated in MHV-ExoN(-) were visualized using *bioinfokit* [66] and further mapped according to their genomic positions. Statistical significance was determined by the p-value of each junction calculated by the DESeq2 package in RStudio and junctions with a significant alteration of abundance in MHV-ExoN(-) compared to MHV-WT were visualized as either red or green in the graph generated by *bioinfokit*.

### Nucleotide composition analysis

DVG junctions were filtered as described above and the nucleotide composition at each position was determined. To avoid bias of highly replicated DVGs and to more closely reflect the stochastic nature of RNA recombination, each unique detected junction was counted equally rather than weighting by read count [67]. Sequences were extracted from a sorted BED file listing the junctions using Rec_Site_Extraction.py with a 30-base pair window. Start site and stop site sequences were separated in Microsoft Excel and the nucleotide frequency at each position was calculated using the *Biostrings* [68] package in RStudio. The mean percentage of a nucleotide was compared between MHV-WT and MHV-ExoN(-) using a 2-way ANOVA test with multiple comparisons and were corrected for false-discovery rate (FDR) using the Benjamini-Hochberg method. Length of microhomology at junction sites were extracted from *ViReMa* SAM file using the Compiler_Module.py of *ViReMa* and -FuzzEntry—Defuzz 0 flags. The frequency of overlaps ranging from 0–10 bp was calculated and compared to an expected probability distribution using uHomology.py.

### Direct RNA Nanopore alignment and analysis

Live basecalling was performed by *Guppy* in *MinKNOW*. Run statistics were generated from each sequencing experiment by *NanoPlot* [69]. Pass reads from all three experiments were concatenated for each virus and aligned to the genome using minimap2 [70] and *FLAIR* (Full Length Alternative Isoforms of RNA) [71] to generate alignment files and BED files listing deletions detected in each sequenced RNA molecule. Both BAM and BED files were filtered for full length molecules using *samtools* and Microsoft Excel, respectively. Full-length CoV molecules were defined as encoding coverage at in the 5’ UTR and 3’ UTR of the respective viruses. Nanopore junctions output in BED files were compared to junctions in *ViReMa* RNA-seq BED files to confirm its presence in both datasets. To account for noisiness in Nanopore datasets, a Nanopore junction was considered confirmed if at least 1 RNA-seq junction start and stop sites fell within 20 bp of the Nanopore start and stop sites, respectively. Filtering of Nanopore and RNA-seq datasets was performed in Microsoft Excel. BED files generated by the flair align module were parsed based on the number of junctions were identified. Nanopore reads containing only 1 junction were identified using Microsoft Excel and unique junctions were quantified in RStudio using base-R functions. Sequencing coverage maps were generated from *samtools* depth analysis of filtered BAM files. All junctions present in sequenced libraries were mapped in Sashimi plots generated by the Integrated Genome Viewer (IGV) [72]. Junctions present in full-length MHV RNA molecules with a single deletion were mapped according to their genomic positions as previously described. The genetic architectures of full-length RNA molecules sequenced by direct RNA Nanopore sequencing were visualized by filtering RNA molecules for at least 3 supporting reads. Low frequency variants were removed from this analysis.

## Supporting information

S1 Fig CoV genome organization and models of recombination. (A) Genome organization of MERS-CoV (gray), SARS-CoV-2 (violet), and MHV (white). Nonstructural (nsps 1–16) and structural (S, E, M, N) and accessory open reading frames (ORFs) are labelled. The common 5’ leader transcription leader sequence (TRS-L) is denoted with an unfilled red star. Body TRSs are labelled with filled red stars. (B) CoVs perform both *trans* (inter-molecular) recombination and *cis* (intra-molecular) recombination and produce 3 different types of molecules: subgenomic mRNAs (sgmRNAs) that are translated into structural and accessory proteins, defective viral genomes (DVGs) whose role in viral replication, innate immune antagonism, and viral evolution have not yet been defined, and infectious (complete) genome molecules. sgmRNAs are produced by recombination between transcription regulatory sequences (TRSs) across the genome. DVGs are produced by recombination between sites across the genome outside TRSs that result in sequence deletions. Complete genomes are generated by recombination at the same location between 2 co-infecting molecules. The CoV replication transcription complex (RTC) is shown in gray. (C) Internally deleted recombined RNAs (DVGs) are formed by a recombination junction (^, white arrow). In this report, a start site refers to the position where the 5’ segment ends (-1, left cyan dashed box) and a stop site refers to the position where the 3’ segment begins (+1, right cyan dashed box) in the viral genome (blue line). Nucleotides sequences in the genome at both the start and stop sites are numbered according to their position relative to the break formed by the recombination junction (red line). (D) Results in this report support the model in which microhomology (yellow box) between the CoV DVG start and stop sites facilitates formation of the complete RNA molecule through translocation of the CoV RTC (gray).
(TIF)

S2 Fig Short-read RNA-sequencing genome coverage and ViReMa-detected recombination junctions in MERS-CoV and SARS-CoV-2, related to Fig 1. RNA-seq libraries of (A) MERS-CoV and (B) SARS-CoV-2 were aligned to the viral genomes with ViReMa. Nucleotide depth was calculated at each position and represented as mean nucleotide depth (N = 3). (C) The number of unique junctions detected was compared between MERS-CoV and SARS-CoV-2. N = 3, error bars represent standard error of the mean. Unpaired student’s t-test, *** p < 0.001. Individual recombination junction scatter plots of (D) MERS-CoV and (E) SARS-CoV-2. Recombination junctions were detected by ViReMa and forward (5’ ➔ 3’) junctions were identified by bioinformatic filtering. Junctions are plotted according to their 5’ (start) and 3’ (stop) positions and colored according to their frequency in the population of total junctions. Highly abundant junctions are magenta and opaque and low-frequency junctions are red and transparent. (F) Relative proportions of junctions forming DVGs, canonical sgmRNAs, and alternative sgmRNAs as a percentage of the total population of all recombined RNA in MERS-CoV (black) and SARS-CoV-2 (violet). N = 3, error bars represent SEM. 2-way ANOVA, *** p < 0.001, **** p < 0.0001. (G) Junction frequency (J_freq_) per 10^4^ mapped nucleotides of MERS-CoV canonical (left, filled circles) and alternative (right, unfilled triangles) sgmRNA species normalized to total viral RNA. N = 3, error bars represent SEM. (H) Junction frequency (J_freq_) per 10^4^ mapped nucleotides of SARS-CoV-2 canonical (left, filled circles) and alternative (right, unfilled triangles) sgmRNA species normalized to total viral RNA. N = 3, error bars represent SEM.
(TIF)

S3 Fig Short-read RNA-sequencing genome coverage and recombination junctions detected by ViReMa in MHV monolayer RNA, related to Fig 3. RNA-seq libraries of (A) MHV-WT and (B) MHV-ExoN(-) infected cell monolayer RNA were aligned to the viral genomes with ViReMa. Nucleotide depth was calculated at each position and represented as mean nucleotide depth (N = 3). (C) The number of unique junctions detected was compared between MHV-WT and MHV-ExoN(-). N = 3, error bars represent standard error of the mean. Unpaired student’s t-test, ** p < 0.01. Individual recombination junction scatter plots of (D) MHV-WT and (E) MHV-ExoN(-). Recombination junctions were detected by ViReMa and forward (5’ ➔ 3’) junctions were identified by bioinformatic filtering. Junctions are plotted according to their 5’ (start) and 3’ (stop) positions and colored according to their frequency in the population of total junctions. Highly abundant junctions are magenta and opaque and low-frequency junctions are red and transparent. (F) Relative proportions of junctions forming DVGs, canonical sgmRNAs, and alternative sgmRNAs in MHV-WT (blue) and MHV-ExoN(-) (orange) infected monolayer RNA. N = 3, error bars represent SEM. 2-way ANOVA, ** p < 0.01, *** p < 0.001, **** p < 0.0001. (G) Ratios of canonical sgmRNA species in MHV-WT (blue) and MHV-ExoN(-) (orange) infected monolayer RNA. Each sgmRNA species is reported as a percentage of the total sgmRNA population. N = 3, error bars represent SEM. 2-way ANOVA, **** p < 0.0001. (H) Ratios of alternative sgmRNA species in MHV-WT (blue) and MHV-ExoN(-) (orange) infected monolayer RNA. Each sgmRNA population is quantified as a percentage of the total number of minor sgmRNA species detected. N = 3, error bars represent SEM. 2-way ANOVA, * p < 0.05, **** p < 0.0001.
(TIF)

S4 Fig Short-read RNA-sequencing genome coverage and recombination junctions detected by ViReMa in MHV viral supernatant RNA, related to Figs 3 and 4. RNA-seq libraries of (A) MHV-WT and (B) MHV-ExoN(-) viral supernatant RNA were aligned to the viral genomes with ViReMa. (C) The number of unique junctions detected was compared between MHV-WT and MHV-ExoN(-). N = 3, error bars represent standard error of the mean. Unpaired student’s t-test, ** p < 0.05. Nucleotide depth was calculated at each position and represented as mean nucleotide depth (N = 3). Individual recombination junction scatter plots of (D) MHV-WT and (E) MHV-ExoN(-). Recombination junctions were detected by ViReMa and forward (5’ ➔ 3’) junctions were identified by bioinformatic filtering. Junctions are plotted according to their 5’ (start) and 3’ (stop) positions and colored according to their frequency in the population of total junctions. Highly abundant junctions are magenta and opaque and low-frequency junctions are red and transparent. (F) Relative proportions of junctions forming DVGs, canonical sgmRNAs, and alternative sgmRNAs in MHV-WT (blue) and MHV-ExoN(-) (orange) viral supernatant RNA. N = 3, error bars represent SEM. 2-way ANOVA, *** p < 0.001, **** p < 0.0001. (G) Ratios of canonical sgmRNA species in MHV-WT (blue) and MHV-ExoN(-) (orange) viral supernatant RNA. Each sgmRNA species is reported as a percentage of the total sgmRNA population. N = 3, error bars represent SEM. 2-way ANOVA, ** p < 0.01, **** p < 0.0001. (H) Ratios of canonical sgmRNA species in MHV-WT (blue) and MHV-ExoN(-) (orange) viral supernatant RNA. Each sgmRNA population is quantified as a percentage of the total number of minor sgmRNA species detected. N = 3, error bars represent SEM. 2-way ANOVA, *** p < 0.001.
(TIF)

S5 Fig MHV-ExoN(-) has significantly altered recombination frequency at multiple positions across the genome and differentially accumulates junctions compared to MHV-WT, related to Fig 4. Mean recombination frequency at each genomic position is shown for MHV-WT (blue) and MHV-ExoN(-) (orange). (A) 5’ UTR, (B) the non-replicase nonstructural proteins (nsp1–6), (C) the replicase proteins (nsp7–16), (D) the structural and accessory proteins, (E) 3’ UTR. Key RNA elements including the TRS-leader (TRS-L) and body TRSs (TRS1–7) are labelled. Positions with statistically significant differences in MHV-ExoN(-) recombination frequency were identified by a 2-way ANOVA with multiple comparisons. Recombination junction abundance was compared in MHV-ExoN(-) to MHV-WT by DESeq2 in infected cell monolayer RNA (A) and viral supernatant RNA (B). Volcano plots of junctions colored by statistical significance (red or green, p < 0.05) and by the log_2_(Fold Change) of abundance (red = downregulated, green = upregulated).
(TIF)

S6 Fig Sequence composition of MHV DVG junction sites in viral supernatant, related to Fig 5. (A) Nucleotide composition was calculated and reported as the percent adenosine (A), cytosine (C), guanine (G), and uracil (U) at each position in a 30-base pair region flanking DVG junction start and stop sites in MHV-WT (blue) and MHV-ExoN(-) (orange) viral supernatant RNA. Each point represents a mean (N = 3) and error bars represent SEM. 2-way ANOVA with multiple comparisons corrected for false discovery rate (FDR) by the Benjamini-Hochberg method. * q < 0.05, ** q < 0.01, *** q < 0.001, **** q < 0.0001. (B) Distribution of microhomology overlaps in MHV-WT (blue) and MHV-ExoN(-) (orange) compared to an expected probability distribution (gray). The frequency of each overlap length is displayed as a mean (N = 3) and error bars represent SEM.
(TIF)

S1 Table Short-read Illumina RNA-seq alignment statistics, related to Figs 1 and 3. Number of reads in RNA-seq libraries and mapped to viral genome reported for MHV, MERS-CoV, and SARS-CoV-2. The percent mapping to the viral genome is reported as a mean of 3 libraries, ± standard error of the mean (SEM).
(PDF)

S2 Table Alignment statistics of Nanopore direct RNA sequencing of MERS-CoV, SARS-CoV-2, MHV-WT and MHV-ExoN(-), related to Figs 2 and 6. For direct RNA Nanopore sequencing of MHV, MERS-CoV, and SARS-CoV-2, the percent identity of aligned reads, the mean read length, mean read quality, the read length N50 (fiftieth percentile), number of total sequenced reads, number of mapped reads, and number of unique detected junctions are reported. The percentage of junctions detected in Nanopore reads also detected in RNA-seq datasets is also reported.
(PDF)

S3 Table Full genome reads of SARS-CoV-2 detected by direct RNA Nanopore sequencing, related to Fig 2. Direct RNA Nanopore reads spanning the entire SARS-CoV-2 genome are listed. The mapping start site (Read Start), mapping end site (Read End), and unique read identifier (Read Name) are all listed. Each read represents a single detection (Count), and contains most of the SARS-CoV-2 genome (Read Length).
(PDF)

S4 Table Direct RNA Nanopore read species, related to Figs 2 and 6. Direct RNA Nanopore reads aligning to viral genome by minimap2. Individual reads are listed by read name. Genomic positions of read alignment are listed (“Read Start”, “Read Stop”). Read segments aligning to the genome are noted (“# Segments”) and start positions and aligned segment lengths listed (“Segment Start”, “Segment Length”).
(XLSB)

S5 Table Genomic positions with significantly altered positional recombination frequency in MHV-ExoN(-) infected monolayer and viral supernatant RNA compared to MHV-WT, related to Fig 4. Positions with significantly altered recombination frequency in MHV-ExoN(-) infected monolayer RNA compared to MHV-WT and in MHV-ExoN(-) viral supernatant RNA compared to MHV-WT as determined by a 2-way ANOVA with multiple comparisons are listed. Genomic regions are noted. (N = 3 for each infected cell and viral supernatant RNA samples)
(PDF)

S6 Table Differential abundance of recombination junctions in MHV-ExoN(-) infected monolayer compared to MHV-WT, related to Fig 4. Junctions with altered abundance in MHV-ExoN(-) infected monolayer RNA compared to MHV-WT and in MHV-ExoN(-) viral supernatant RNA compared to MHV-WT are listed. P-values calculated by DESeq2. (N = 3 for each infected monolayer and viral supernatant RNA samples)
(XLSB)
