# Supplementary figures and images for: The coronavirus proofreading exoribonuclease mediates extensive viral recombination
Source: PLoS Pathog. 2021 Jan 19;17(1):e1009226. doi: 10.1371/journal.ppat.1009226 (PMC7846108; doi:10.1371/journal.ppat.1009226)

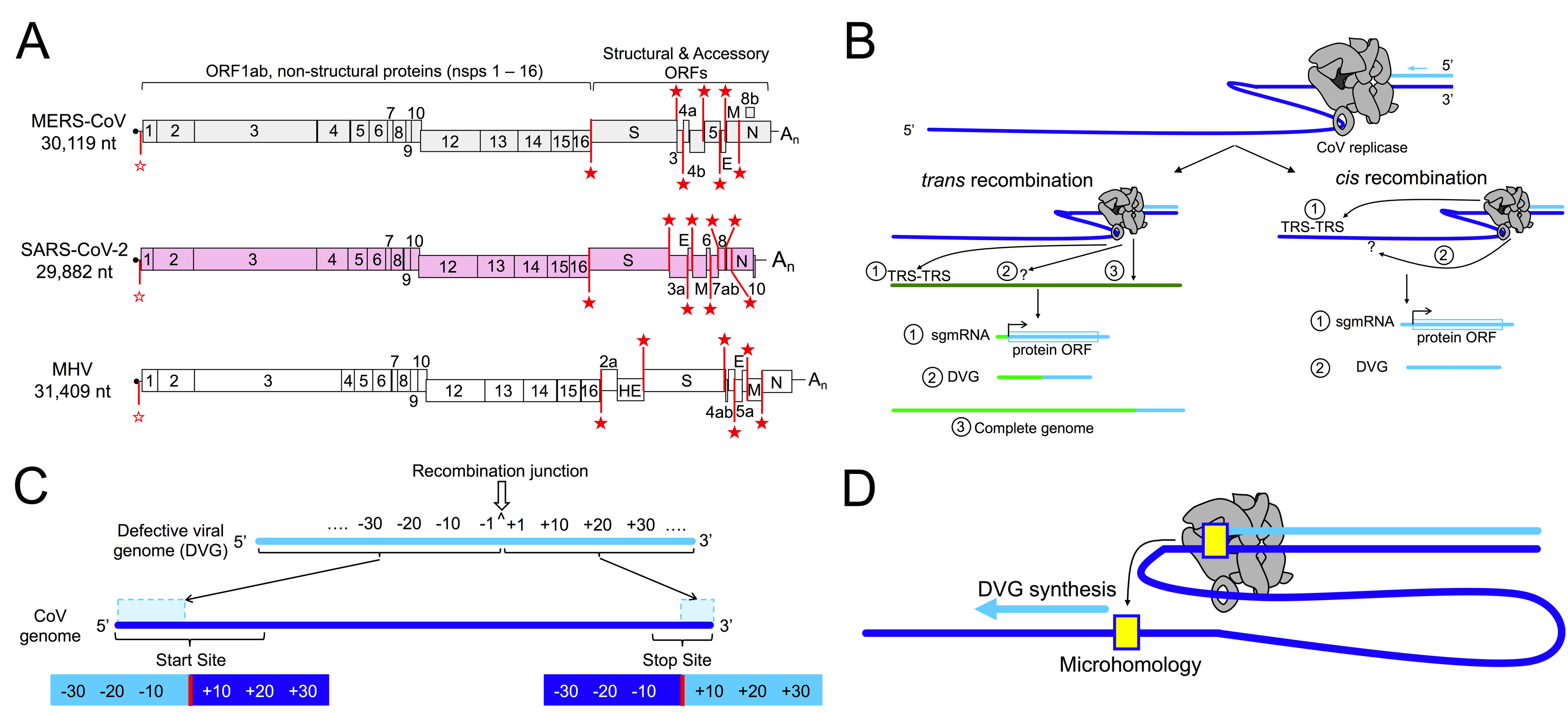

Supplement: S1 Fig — (A) Genome organization of MERS-CoV (gray), SARS-CoV-2 (violet), and MHV (white). Nonstructural (nsps 1–16) and structural (S, E, M, N) and accessory open reading frames (ORFs) are labelled. The common 5’ leader transcription leader sequence (TRS-L) is denoted with an unfilled red star. Body TRSs are labelled with filled red stars. (B) CoVs perform both trans (inter-molecular) recombination and cis (intra-molecular) recombination and produce 3 different types of molecules: subgenomic mRNAs (sgmRNAs) that are translated into structural and accessory proteins, defective viral genomes (DVGs) whose role in viral replication, innate immune antagonism, and viral evolution have not yet been defined, and infectious (complete) genome molecules. sgmRNAs are produced by recombination between transcription regulatory sequences (TRSs) across the genome. DVGs are produced by recombination between sites across the genome outside TRSs that result in sequence deletions. Complete genomes are generated by recombination at the same location between 2 co-infecting molecules. The CoV replication transcription complex (RTC) is shown in gray. (C) Internally deleted recombined RNAs (DVGs) are formed by a recombination junction (^, white arrow). In this report, a start site refers to the position where the 5’ segment ends (-1, left cyan dashed box) and a stop site refers to the position where the 3’ segment begins (+1, right cyan dashed box) in the viral genome (blue line). Nucleotides sequences in the genome at both the start and stop sites are numbered according to their position relative to the break formed by the recombination junction (red line). (D) Results in this report support the model in which microhomology (yellow box) between the CoV DVG start and stop sites facilitates formation of the complete RNA molecule through translocation of the CoV RTC (gray). (TIF) [file ppat.1009226.s001.tif]

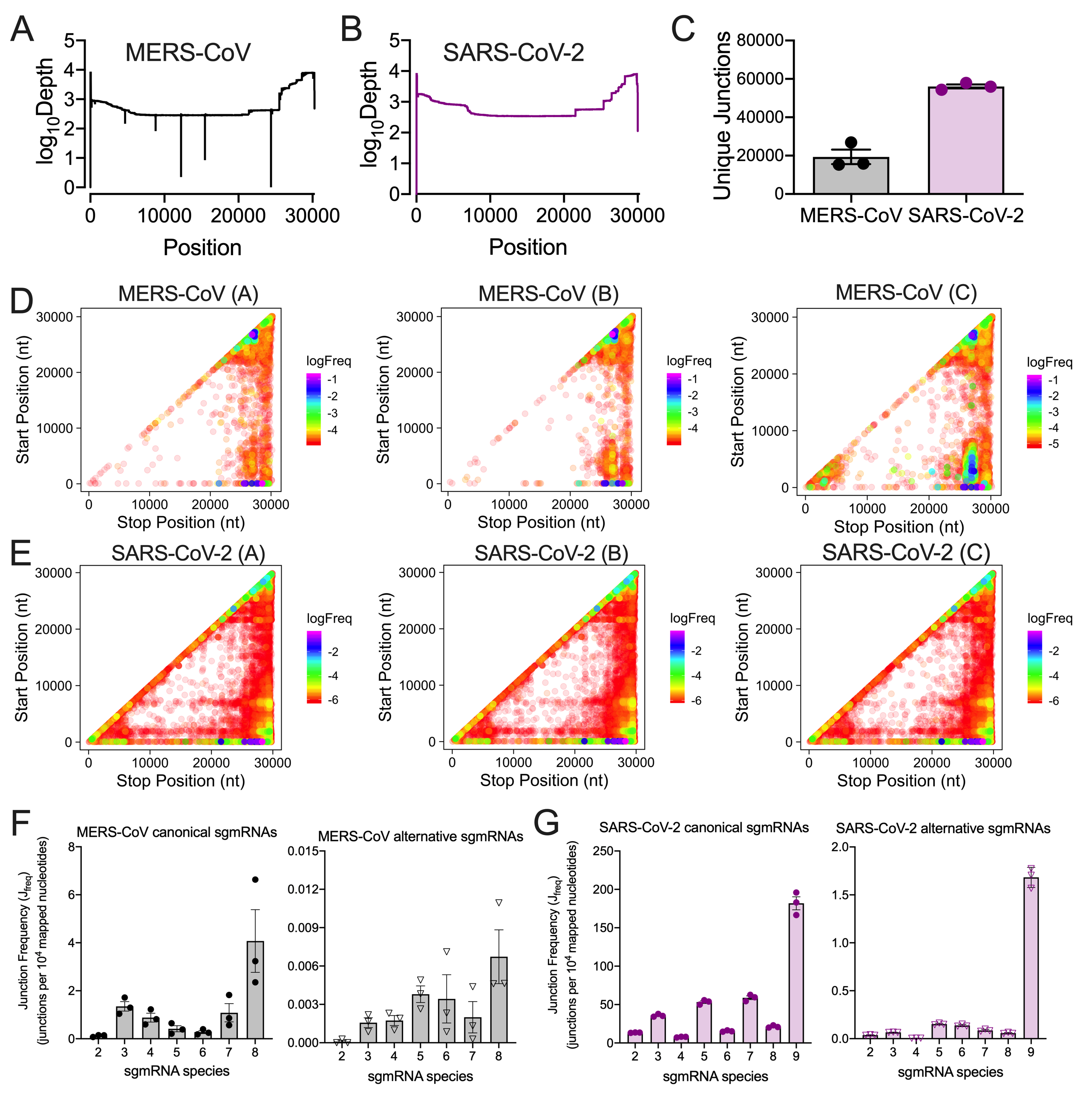

Supplement: S2 Fig — RNA-seq libraries of (A) MERS-CoV and (B) SARS-CoV-2 were aligned to the viral genomes with ViReMa. Nucleotide depth was calculated at each position and represented as mean nucleotide depth (N = 3). (C) The number of unique junctions detected was compared between MERS-CoV and SARS-CoV-2. N = 3, error bars represent standard error of the mean. Unpaired student’s t-test, *** p < 0.001. Individual recombination junction scatter plots of (D) MERS-CoV and (E) SARS-CoV-2. Recombination junctions were detected by ViReMa and forward (5’ ➔ 3’) junctions were identified by bioinformatic filtering. Junctions are plotted according to their 5’ (start) and 3’ (stop) positions and colored according to their frequency in the population of total junctions. Highly abundant junctions are magenta and opaque and low-frequency junctions are red and transparent. (F) Relative proportions of junctions forming DVGs, canonical sgmRNAs, and alternative sgmRNAs as a percentage of the total population of all recombined RNA in MERS-CoV (black) and SARS-CoV-2 (violet). N = 3, error bars represent SEM. 2-way ANOVA, *** p < 0.001, **** p < 0.0001. (G) Junction frequency (Jfreq) per 104 mapped nucleotides of MERS-CoV canonical (left, filled circles) and alternative (right, unfilled triangles) sgmRNA species normalized to total viral RNA. N = 3, error bars represent SEM. (H) Junction frequency (Jfreq) per 104 mapped nucleotides of SARS-CoV-2 canonical (left, filled circles) and alternative (right, unfilled triangles) sgmRNA species normalized to total viral RNA. N = 3, error bars represent SEM. (TIF) [file ppat.1009226.s002.tif]

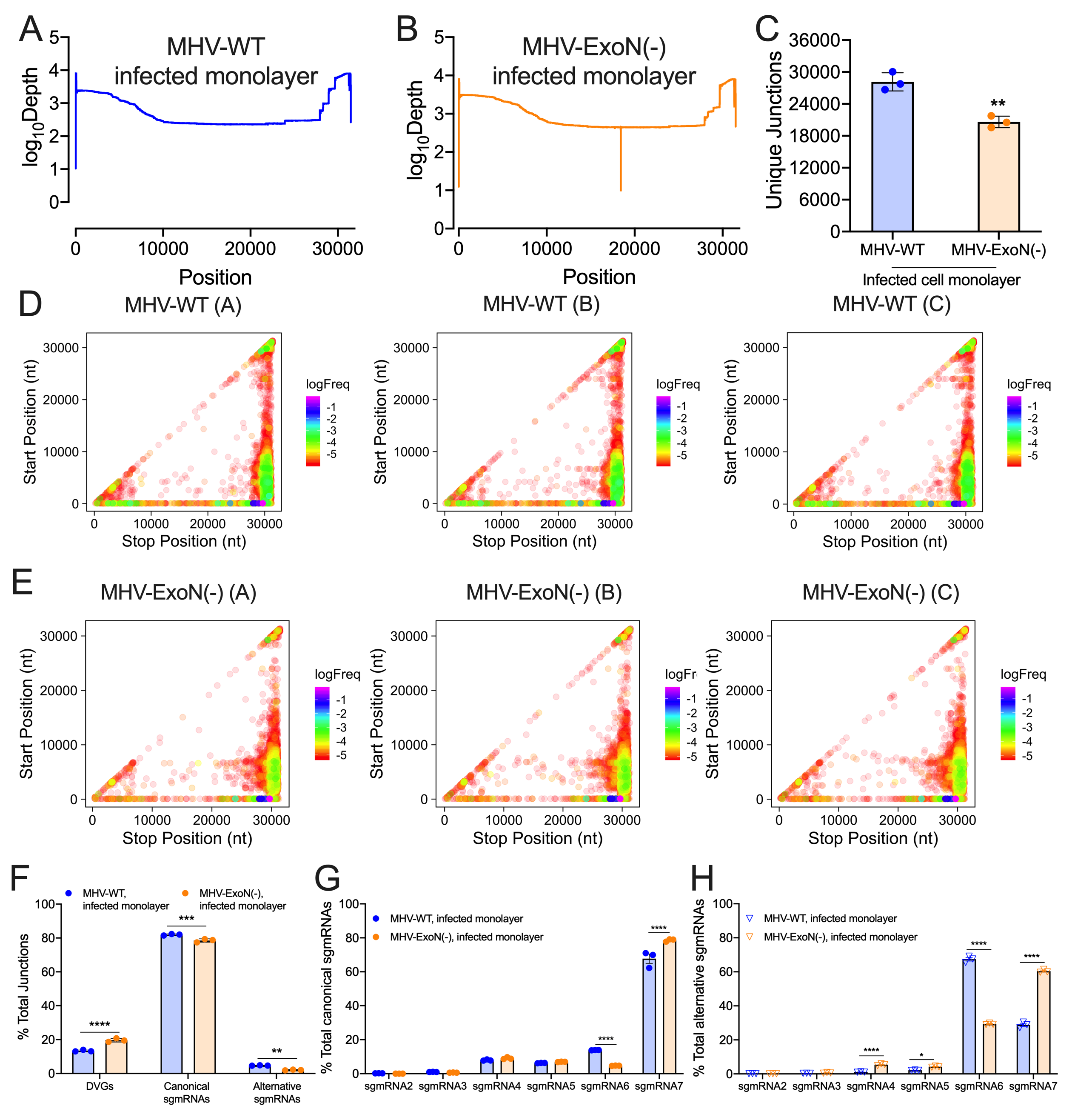

Supplement: S3 Fig — RNA-seq libraries of (A) MHV-WT and (B) MHV-ExoN(-) infected cell monolayer RNA were aligned to the viral genomes with ViReMa. Nucleotide depth was calculated at each position and represented as mean nucleotide depth (N = 3). (C) The number of unique junctions detected was compared between MHV-WT and MHV-ExoN(-). N = 3, error bars represent standard error of the mean. Unpaired student’s t-test, ** p < 0.01. Individual recombination junction scatter plots of (D) MHV-WT and (E) MHV-ExoN(-). Recombination junctions were detected by ViReMa and forward (5’ ➔ 3’) junctions were identified by bioinformatic filtering. Junctions are plotted according to their 5’ (start) and 3’ (stop) positions and colored according to their frequency in the population of total junctions. Highly abundant junctions are magenta and opaque and low-frequency junctions are red and transparent. (F) Relative proportions of junctions forming DVGs, canonical sgmRNAs, and alternative sgmRNAs in MHV-WT (blue) and MHV-ExoN(-) (orange) infected monolayer RNA. N = 3, error bars represent SEM. 2-way ANOVA, ** p < 0.01, *** p < 0.001, **** p < 0.0001. (G) Ratios of canonical sgmRNA species in MHV-WT (blue) and MHV-ExoN(-) (orange) infected monolayer RNA. Each sgmRNA species is reported as a percentage of the total sgmRNA population. N = 3, error bars represent SEM. 2-way ANOVA, **** p < 0.0001. (H) Ratios of alternative sgmRNA species in MHV-WT (blue) and MHV-ExoN(-) (orange) infected monolayer RNA. Each sgmRNA population is quantified as a percentage of the total number of minor sgmRNA species detected. N = 3, error bars represent SEM. 2-way ANOVA, * p < 0.05, **** p < 0.0001. (TIF) [file ppat.1009226.s003.tif]

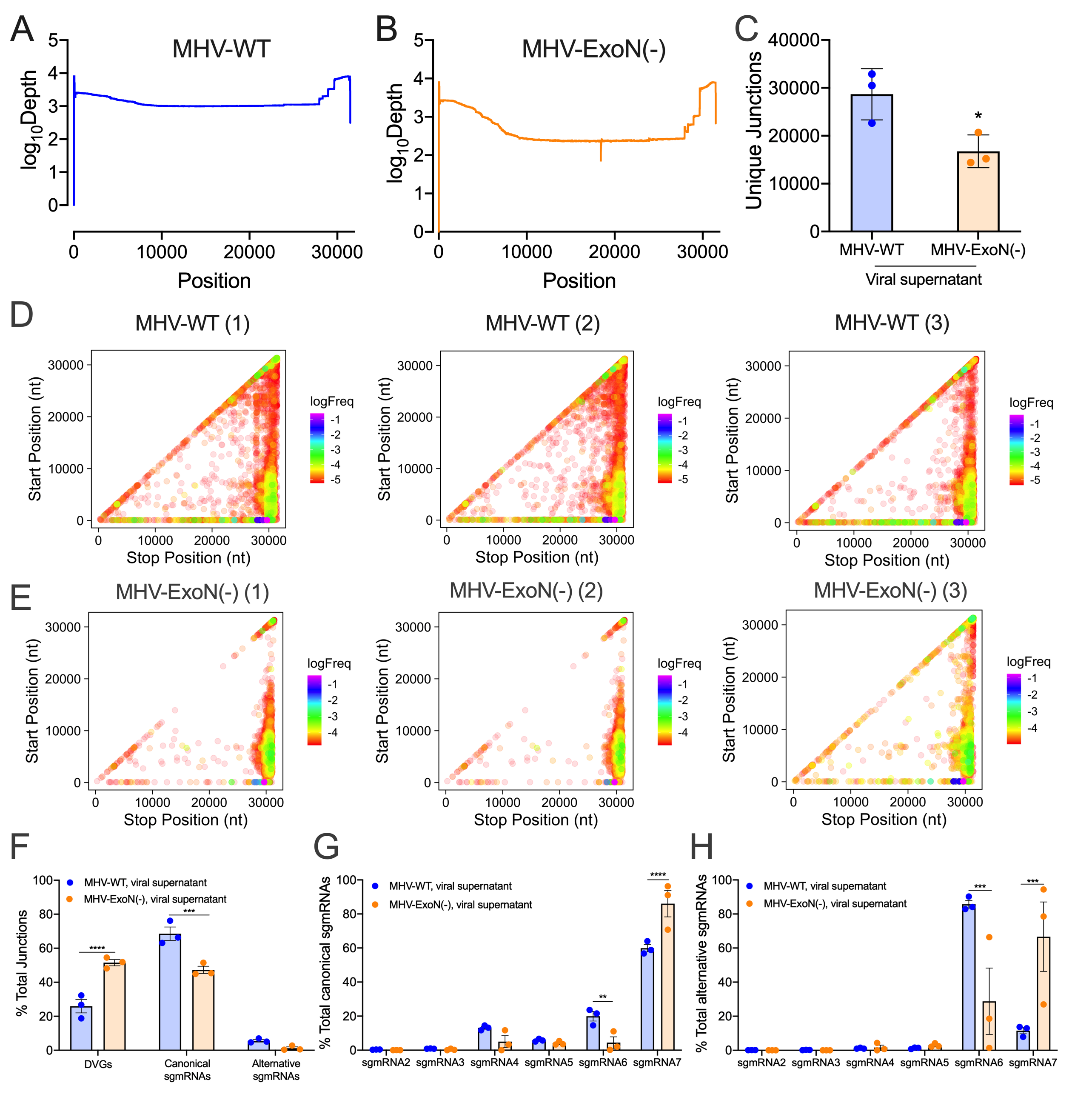

Supplement: S4 Fig — RNA-seq libraries of (A) MHV-WT and (B) MHV-ExoN(-) viral supernatant RNA were aligned to the viral genomes with ViReMa. (C) The number of unique junctions detected was compared between MHV-WT and MHV-ExoN(-). N = 3, error bars represent standard error of the mean. Unpaired student’s t-test, ** p < 0.05. Nucleotide depth was calculated at each position and represented as mean nucleotide depth (N = 3). Individual recombination junction scatter plots of (D) MHV-WT and (E) MHV-ExoN(-). Recombination junctions were detected by ViReMa and forward (5’ ➔ 3’) junctions were identified by bioinformatic filtering. Junctions are plotted according to their 5’ (start) and 3’ (stop) positions and colored according to their frequency in the population of total junctions. Highly abundant junctions are magenta and opaque and low-frequency junctions are red and transparent. (F) Relative proportions of junctions forming DVGs, canonical sgmRNAs, and alternative sgmRNAs in MHV-WT (blue) and MHV-ExoN(-) (orange) viral supernatant RNA. N = 3, error bars represent SEM. 2-way ANOVA, *** p < 0.001, **** p < 0.0001. (G) Ratios of canonical sgmRNA species in MHV-WT (blue) and MHV-ExoN(-) (orange) viral supernatant RNA. Each sgmRNA species is reported as a percentage of the total sgmRNA population. N = 3, error bars represent SEM. 2-way ANOVA, ** p < 0.01, **** p < 0.0001. (H) Ratios of canonical sgmRNA species in MHV-WT (blue) and MHV-ExoN(-) (orange) viral supernatant RNA. Each sgmRNA population is quantified as a percentage of the total number of minor sgmRNA species detected. N = 3, error bars represent SEM. 2-way ANOVA, *** p < 0.001. (TIF) [file ppat.1009226.s004.tif]

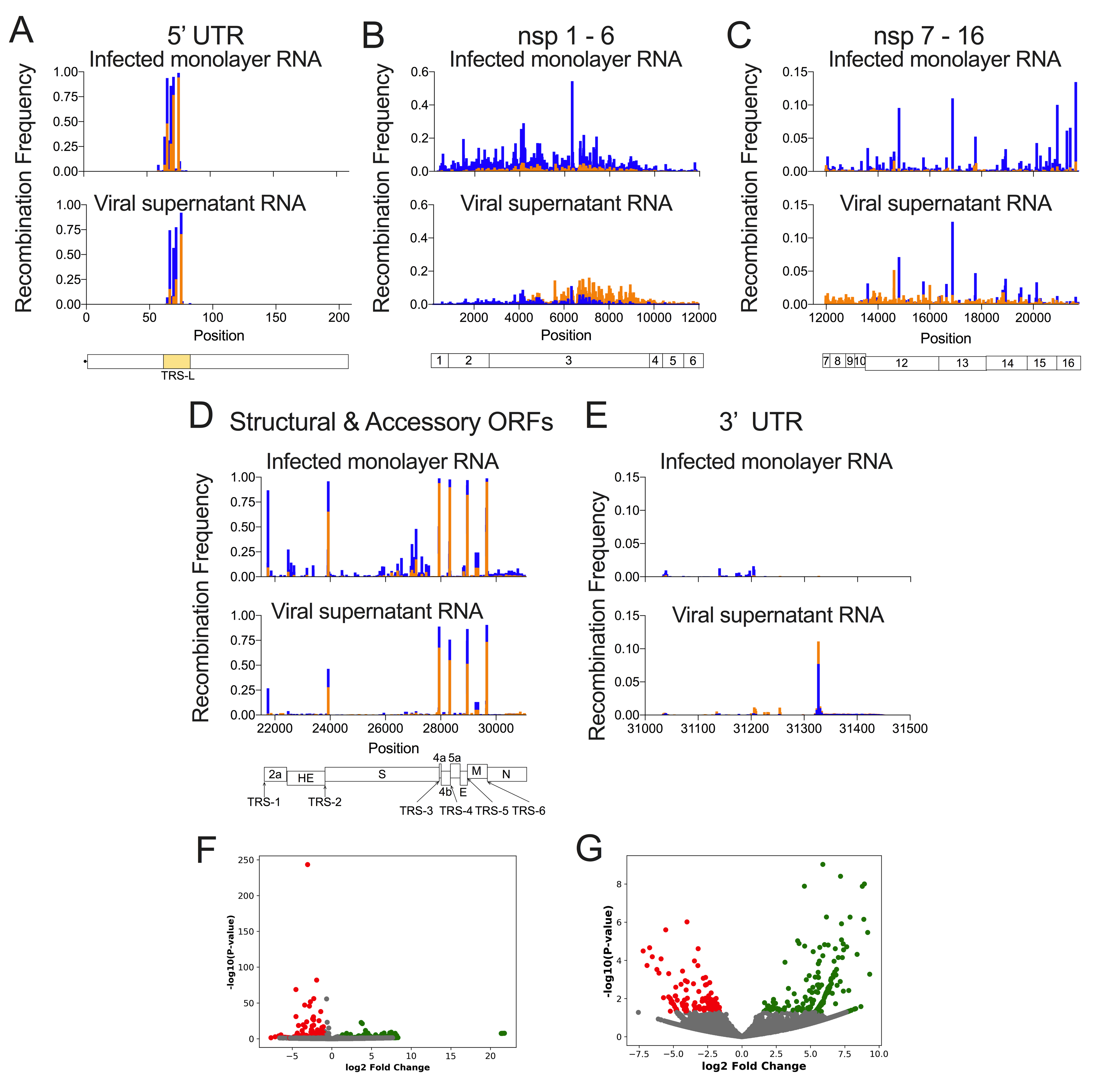

Supplement: S5 Fig — Mean recombination frequency at each genomic position is shown for MHV-WT (blue) and MHV-ExoN(-) (orange). (A) 5’ UTR, (B) the non-replicase nonstructural proteins (nsp1–6), (C) the replicase proteins (nsp7–16), (D) the structural and accessory proteins, (E) 3’ UTR. Key RNA elements including the TRS-leader (TRS-L) and body TRSs (TRS1–7) are labelled. Positions with statistically significant differences in MHV-ExoN(-) recombination frequency were identified by a 2-way ANOVA with multiple comparisons. Recombination junction abundance was compared in MHV-ExoN(-) to MHV-WT by DESeq2 in infected cell monolayer RNA (A) and viral supernatant RNA (B). Volcano plots of junctions colored by statistical significance (red or green, p < 0.05) and by the log2(Fold Change) of abundance (red = downregulated, green = upregulated). (TIF) [file ppat.1009226.s005.tif]

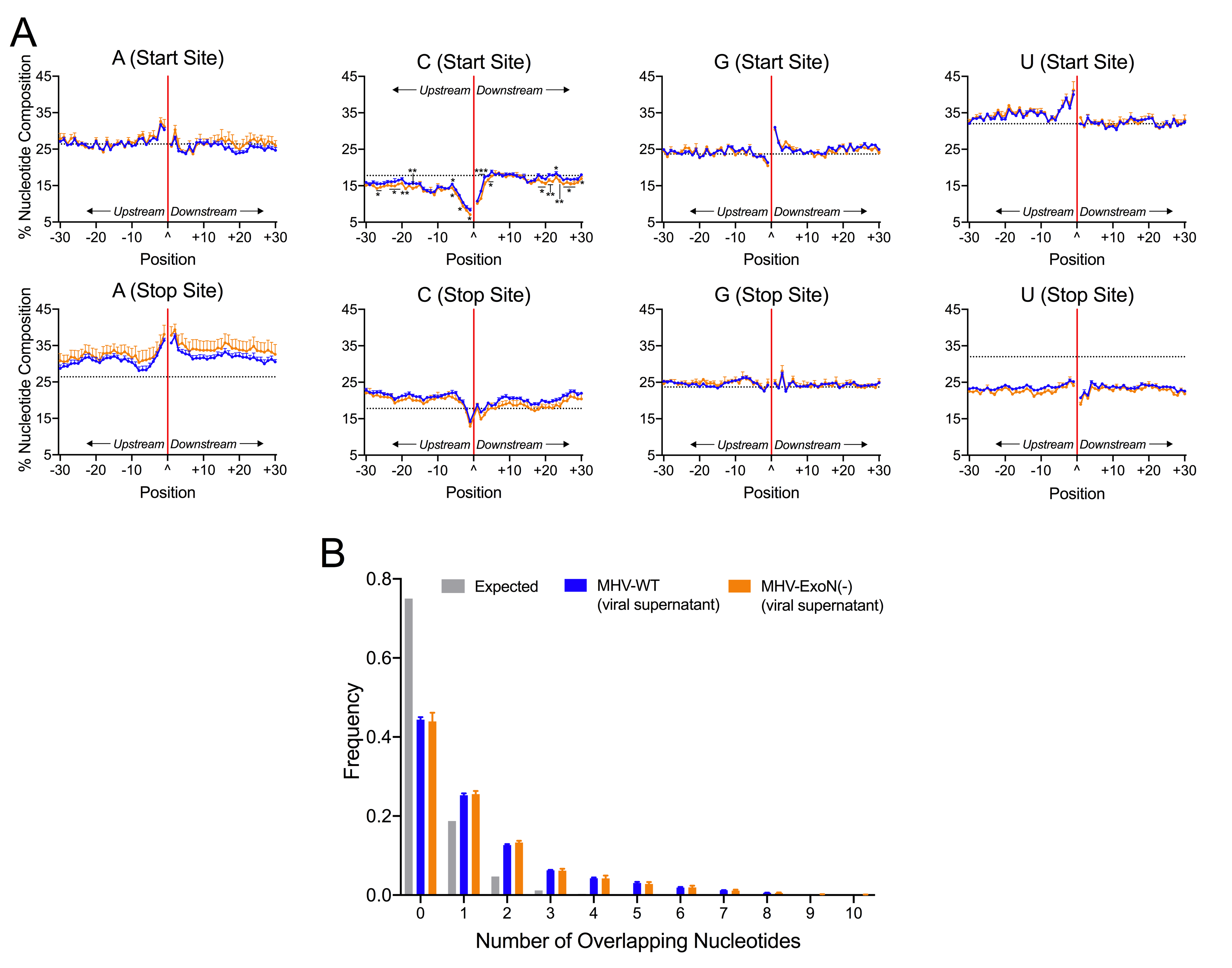

Supplement: S6 Fig — (A) Nucleotide composition was calculated and reported as the percent adenosine (A), cytosine (C), guanine (G), and uracil (U) at each position in a 30-base pair region flanking DVG junction start and stop sites in MHV-WT (blue) and MHV-ExoN(-) (orange) viral supernatant RNA. Each point represents a mean (N = 3) and error bars represent SEM. 2-way ANOVA with multiple comparisons corrected for false discovery rate (FDR) by the Benjamini-Hochberg method. * q < 0.05, ** q < 0.01, *** q < 0.001, **** q < 0.0001. (B) Distribution of microhomology overlaps in MHV-WT (blue) and MHV-ExoN(-) (orange) compared to an expected probability distribution (gray). The frequency of each overlap length is displayed as a mean (N = 3) and error bars represent SEM. (TIF) [file ppat.1009226.s006.tif]
