# Supplementary material for: The coronavirus proofreading exoribonuclease mediates extensive viral recombination
Source: PLoS Pathog. 2021 Jan 19;17(1):e1009226. doi: 10.1371/journal.ppat.1009226 (PMC7846108; doi:10.1371/journal.ppat.1009226)
Supplement: S1 Table — Number of reads in RNA-seq libraries and mapped to viral genome reported for MHV, MERS-CoV, and SARS-CoV-2. The percent mapping to the viral genome is reported as a mean of 3 libraries, ± standard error of the mean (SEM). (PDF) [file ppat.1009226.s007.pdf]

	Total Reads	Viral Reads	Mean % Mapping to Virus (± SEM)
MERS-CoV	41272492<br>43952576<br>60016392	35144605<br>36874175<br>47902083	82.95 ± 1.61
SARS-CoV-2	96269559<br>104753240<br>101519351	72415125<br>81433589<br>78799665	77.48 ± 0.20
MHV-WT<br>(infected cell monolayer)	76403024<br>72081685<br>72305104	45626943<br>42808724<br>42472537	59.28 ± 0.29
MHV-ExoN(-)<br>(infected cell monolayer)	74803163<br>80268491<br>81780390	12202990<br>12612143<br>14681501	16.66 ± 0.67
MHV-WT<br>(viral supernatant)	32200976<br>50152822<br>35221216	30679479<br>47151310<br>33941750	95.22 ± 0.68
MHV-ExoN(-)<br>(viral supernatant)	81636281<br>61064695<br>52274504	6170745<br>9606775<br>18688288	19.80 ± 8.37
