# Supplementary material for: The coronavirus proofreading exoribonuclease mediates extensive viral recombination
Source: PLoS Pathog. 2021 Jan 19;17(1):e1009226. doi: 10.1371/journal.ppat.1009226 (PMC7846108; doi:10.1371/journal.ppat.1009226)
Supplement: S2 Table — For direct RNA Nanopore sequencing of MHV, MERS-CoV, and SARS-CoV-2, the percent identity of aligned reads, the mean read length, mean read quality, the read length N50 (fiftieth percentile), number of total sequenced reads, number of mapped reads, and number of unique detected junctions are reported. The percentage of junctions detected in Nanopore reads also detected in RNA-seq datasets is also reported. (PDF) [file ppat.1009226.s008.pdf]

Virus	Mean % Identity	Mean Read Length	Mean Read Quality	Read Length N50	Total Sequenced Reads	Viral Mapping Reads
MERS-CoV	85.6	773.8	8.4	1014	626548	178658
SARS-CoV-2	82.2	1555.8	8.9	1952	2298107	1725862
MHV-WT	86.7	1175.7	9	1678	766900	102267
MHV-ExoN(-)	86.8	1062.3	9.1	1483	1340286	19445

Unique junctions in complete reads	% Junctions confirmed in RNA-seq
473	89.29
181,770	97.97
5,273	96
195	97.5
