# Supplementary material for: The coronavirus proofreading exoribonuclease mediates extensive viral recombination
Source: PLoS Pathog. 2021 Jan 19;17(1):e1009226. doi: 10.1371/journal.ppat.1009226 (PMC7846108; doi:10.1371/journal.ppat.1009226)
Supplement: S3 Table — Direct RNA Nanopore reads spanning the entire SARS-CoV-2 genome are listed. The mapping start site (Read Start), mapping end site (Read End), and unique read identifier (Read Name) are all listed. Each read represents a single detection (Count), and contains most of the SARS-CoV-2 genome (Read Length). (PDF) [file ppat.1009226.s009.pdf]

Genome	Read Start (nt)	Read End (nt)	Read Name	Read Length	Count
MT02088.1	10	29691	103efdf4-a528-46e3-b5bb-b360e2cae18b;0	29681	1
MT02088.1	11	29863	41da8a52-cb9e-4969-95eb-5fd13b65584b;0	29852	1
MT02088.1	14	29874	cb66c733-0ad3-493c-8a9f-310bbd96e6fe;0	29860	1
