# Supplementary material for: The coronavirus proofreading exoribonuclease mediates extensive viral recombination
Source: PLoS Pathog. 2021 Jan 19;17(1):e1009226. doi: 10.1371/journal.ppat.1009226 (PMC7846108; doi:10.1371/journal.ppat.1009226)
Supplement: S5 Table — Positions with significantly altered recombination frequency in MHV-ExoN(-) infected monolayer RNA compared to MHV-WT and in MHV-ExoN(-) viral supernatant RNA compared to MHV-WT as determined by a 2-way ANOVA with multiple comparisons are listed. Genomic regions are noted. (N = 3 for each infected cell and viral supernatant RNA samples) (PDF) [file ppat.1009226.s011.pdf]

Position	Mean Diff	95% CI of diff	Significant?	Summary	Adjusted p-value
59	0.05466	0.03121 to 0.07811	Yes	****	<0.0001
64	0.2822	0.2588 to 0.3057	Yes	****	<0.0001
65	0.04973	0.02628 to 0.07319	Yes	****	<0.0001
66	0.4556	0.4321 to 0.4790	Yes	****	<0.0001
67	0.2899	0.2664 to 0.3134	Yes	****	<0.0001
68	0.273	0.2496 to 0.2965	Yes	****	<0.0001
69	0.5804	0.5569 to 0.6038	Yes	****	<0.0001
70	0.07626	0.05281 to 0.09971	Yes	****	<0.0001
71	0.1814	0.1579 to 0.2048	Yes	****	<0.0001
73	0.02455	0.001092 to 0.04800	Yes	*	0.0163
75	0.04579	0.02234 to 0.06925	Yes	****	<0.0001
76	0.07531	0.05185 to 0.09876	Yes	****	<0.0001
475	0.0344	0.01095 to 0.05786	Yes	****	<0.0001
533	0.04343	0.01998 to 0.06689	Yes	****	<0.0001
587	0.1043	0.08082 to 0.1277	Yes	****	<0.0001
659	0.03491	0.01145 to 0.05836	Yes	****	<0.0001
665	0.03666	0.01320 to 0.06011	Yes	****	<0.0001
673	0.0301	0.006645 to 0.05355	Yes	****	<0.0001
701	0.0294	0.005947 to 0.05285	Yes	****	<0.0001
973	0.0279	0.004444 to 0.05135	Yes	***	0.0004
999	0.02884	0.005386 to 0.05229	Yes	***	0.0001
1010	0.03215	0.008699 to 0.05561	Yes	****	<0.0001
1041	0.06281	0.03936 to 0.08627	Yes	****	<0.0001
1054	0.03512	0.01166 to 0.05857	Yes	****	<0.0001
1110	0.05034	0.02689 to 0.07379	Yes	****	<0.0001
1250	0.02505	0.001594 to 0.04850	Yes	**	0.0095
1255	0.0558	0.03234 to 0.07925	Yes	****	<0.0001
1276	0.04119	0.01774 to 0.06465	Yes	****	<0.0001
1311	0.0288	0.005349 to 0.05226	Yes	***	0.0001
1382	0.02898	0.005525 to 0.05243	Yes	****	<0.0001
1422	0.0299	0.006450 to 0.05336	Yes	****	<0.0001
1495	0.1915	0.1681 to 0.2150	Yes	****	<0.0001
1499	0.04143	0.01798 to 0.06488	Yes	****	<0.0001
1501	0.09985	0.07639 to 0.1233	Yes	****	<0.0001
1522	0.05009	0.02663 to 0.07354	Yes	****	<0.0001
1524	0.05727	0.03382 to 0.08072	Yes	****	<0.0001
1559	0.04634	0.02289 to 0.06980	Yes	****	<0.0001
1582	0.036	0.01254 to 0.05945	Yes	****	<0.0001
1583	0.03453	0.01108 to 0.05799	Yes	****	<0.0001
1618	0.05413	0.03068 to 0.07758	Yes	****	<0.0001
1626	0.06802	0.04457 to 0.09148	Yes	****	<0.0001
1627	0.04333	0.01988 to 0.06678	Yes	****	<0.0001
1638	0.02453	0.001079 to 0.04799	Yes	*	0.0165
1696	0.02886	0.005409 to 0.05232	Yes	***	0.0001

1708	0.02575	0.002298 to 0.04920	Yes	**	0.0044
1748	0.02717	0.003713 to 0.05062	Yes	***	0.0009
1771	0.03418	0.01073 to 0.05764	Yes	****	<0.0001
1812	0.02863	0.005177 to 0.05208	Yes	***	0.0002
1864	0.04321	0.01975 to 0.06666	Yes	****	<0.0001
1865	0.0392	0.01575 to 0.06266	Yes	****	<0.0001
1868	0.02954	0.006085 to 0.05299	Yes	****	<0.0001
1872	0.05694	0.03349 to 0.08040	Yes	****	<0.0001
1875	0.02551	0.002052 to 0.04896	Yes	**	0.0058
1973	0.02953	0.006074 to 0.05298	Yes	****	<0.0001
1975	0.04786	0.02440 to 0.07131	Yes	****	<0.0001
1976	0.032	0.008551 to 0.05546	Yes	****	<0.0001
1985	0.05401	0.03055 to 0.07746	Yes	****	<0.0001
1990	0.03363	0.01018 to 0.05708	Yes	****	<0.0001
2050	0.05444	0.03098 to 0.07789	Yes	****	<0.0001
2087	0.07132	0.04787 to 0.09477	Yes	****	<0.0001
2088	0.05707	0.03361 to 0.08052	Yes	****	<0.0001
2089	0.03114	0.007685 to 0.05459	Yes	****	<0.0001
2090	0.06635	0.04290 to 0.08980	Yes	****	<0.0001
2091	0.02583	0.002377 to 0.04928	Yes	**	0.004
2092	0.02617	0.002719 to 0.04963	Yes	**	0.0028
2093	0.06055	0.03709 to 0.08400	Yes	****	<0.0001
2095	0.04793	0.02448 to 0.07139	Yes	****	<0.0001
2096	0.02718	0.003730 to 0.05064	Yes	***	0.0009
2097	0.02561	0.002156 to 0.04906	Yes	**	0.0052
2099	0.02632	0.002863 to 0.04977	Yes	**	0.0023
2100	0.03315	0.009697 to 0.05660	Yes	****	<0.0001
2110	0.02781	0.004358 to 0.05127	Yes	***	0.0004
2171	0.04524	0.02179 to 0.06870	Yes	****	<0.0001
2172	0.03655	0.01310 to 0.06001	Yes	****	<0.0001
2173	0.1282	0.1048 to 0.1517	Yes	****	<0.0001
2174	0.08203	0.05858 to 0.1055	Yes	****	<0.0001
2237	0.02592	0.002471 to 0.04938	Yes	**	0.0036
2268	0.03777	0.01432 to 0.06123	Yes	****	<0.0001
2270	0.04071	0.01726 to 0.06417	Yes	****	<0.0001
2271	0.02796	0.004506 to 0.05141	Yes	***	0.0003
2272	0.02996	0.006505 to 0.05341	Yes	****	<0.0001
2298	0.03735	0.01389 to 0.06080	Yes	****	<0.0001
2341	0.04959	0.02614 to 0.07305	Yes	****	<0.0001
2356	0.02951	0.006060 to 0.05297	Yes	****	<0.0001
2439	0.125	0.1016 to 0.1485	Yes	****	<0.0001
2520	0.02685	0.003393 to 0.05030	Yes	**	0.0013
2525	0.02599	0.002541 to 0.04945	Yes	**	0.0034
2533	0.04021	0.01675 to 0.06366	Yes	****	<0.0001
2536	0.02756	0.004104 to 0.05101	Yes	***	0.0006

2537	0.03948	0.01603 to 0.06294	Yes	****	<0.0001
2538	0.04078	0.01733 to 0.06424	Yes	****	<0.0001
2539	0.03392	0.01047 to 0.05738	Yes	****	<0.0001
2540	0.06333	0.03987 to 0.08678	Yes	****	<0.0001
2541	0.06241	0.03896 to 0.08586	Yes	****	<0.0001
2567	0.02416	0.0007031 to 0.04761	Yes	*	0.0245
2569	0.02974	0.006287 to 0.05319	Yes	****	<0.0001
2585	0.02378	0.0003239 to 0.04723	Yes	*	0.0361
2593	0.03362	0.01016 to 0.05707	Yes	****	<0.0001
2594	0.02489	0.001441 to 0.04835	Yes	*	0.0113
2599	0.06378	0.04033 to 0.08723	Yes	****	<0.0001
2600	0.05409	0.03064 to 0.07755	Yes	****	<0.0001
2608	0.06512	0.04167 to 0.08858	Yes	****	<0.0001
2609	0.03223	0.008782 to 0.05569	Yes	****	<0.0001
2611	0.03209	0.008640 to 0.05555	Yes	****	<0.0001
2617	0.0572	0.03374 to 0.08065	Yes	****	<0.0001
2668	0.04948	0.02603 to 0.07294	Yes	****	<0.0001
2669	0.02599	0.002535 to 0.04944	Yes	**	0.0034
2686	0.02615	0.002693 to 0.04960	Yes	**	0.0028
2704	0.05421	0.03076 to 0.07767	Yes	****	<0.0001
2723	0.03075	0.007301 to 0.05421	Yes	****	<0.0001
2763	0.03742	0.01397 to 0.06087	Yes	****	<0.0001
2764	0.03974	0.01629 to 0.06320	Yes	****	<0.0001
2766	0.026	0.002543 to 0.04945	Yes	**	0.0034
2768	0.02489	0.001439 to 0.04835	Yes	*	0.0113
2769	0.04089	0.01744 to 0.06435	Yes	****	<0.0001
2770	0.04233	0.01888 to 0.06579	Yes	****	<0.0001
2772	0.03667	0.01322 to 0.06013	Yes	****	<0.0001
2831	0.02354	9.117e-005 to 0.04700	Yes	*	0.0457
2854	0.04728	0.02383 to 0.07073	Yes	****	<0.0001
2881	0.06043	0.03698 to 0.08388	Yes	****	<0.0001
2921	0.02416	0.0007087 to 0.04762	Yes	*	0.0243
2938	0.1296	0.1062 to 0.1531	Yes	****	<0.0001
2968	0.05794	0.03449 to 0.08140	Yes	****	<0.0001
2978	0.02965	0.006194 to 0.05310	Yes	****	<0.0001
2988	0.04055	0.01710 to 0.06400	Yes	****	<0.0001
2990	0.04159	0.01814 to 0.06504	Yes	****	<0.0001
2993	0.02417	0.0007175 to 0.04762	Yes	*	0.0241
2994	0.02795	0.004500 to 0.05141	Yes	***	0.0003
3075	0.05485	0.03140 to 0.07831	Yes	****	<0.0001
3076	0.0372	0.01375 to 0.06066	Yes	****	<0.0001
3105	0.0325	0.009044 to 0.05595	Yes	****	<0.0001
3106	0.05109	0.02763 to 0.07454	Yes	****	<0.0001
3113	0.03293	0.009481 to 0.05639	Yes	****	<0.0001
3140	0.02565	0.002193 to 0.04910	Yes	**	0.005

3141	0.03662	0.01317 to 0.06008	Yes	****	<0.0001
3142	0.05796	0.03450 to 0.08141	Yes	****	<0.0001
3211	0.04075	0.01729 to 0.06420	Yes	****	<0.0001
3223	0.03271	0.009252 to 0.05616	Yes	****	<0.0001
3243	0.1471	0.1237 to 0.1706	Yes	****	<0.0001
3245	0.04475	0.02130 to 0.06821	Yes	****	<0.0001
3430	0.04194	0.01848 to 0.06539	Yes	****	<0.0001
3431	0.02501	0.001553 to 0.04846	Yes	**	0.01
3438	0.02751	0.004052 to 0.05096	Yes	***	0.0006
3514	0.03744	0.01399 to 0.06089	Yes	****	<0.0001
3595	0.03497	0.01152 to 0.05842	Yes	****	<0.0001
3617	0.03031	0.006861 to 0.05377	Yes	****	<0.0001
3631	0.03817	0.01472 to 0.06163	Yes	****	<0.0001
3632	0.0258	0.002347 to 0.04925	Yes	**	0.0042
3633	0.03149	0.008032 to 0.05494	Yes	****	<0.0001
3635	0.02988	0.006425 to 0.05333	Yes	****	<0.0001
3637	0.02389	0.0004350 to 0.04734	Yes	*	0.0323
3638	0.03211	0.008659 to 0.05557	Yes	****	<0.0001
3639	0.03832	0.01486 to 0.06177	Yes	****	<0.0001
3640	0.03325	0.009794 to 0.05670	Yes	****	<0.0001
3645	0.03157	0.008116 to 0.05502	Yes	****	<0.0001
3720	0.07776	0.05430 to 0.1012	Yes	****	<0.0001
3728	0.02383	0.0003766 to 0.04728	Yes	*	0.0342
3729	0.03285	0.009398 to 0.05630	Yes	****	<0.0001
3737	0.04329	0.01984 to 0.06675	Yes	****	<0.0001
3738	0.0246	0.001151 to 0.04806	Yes	*	0.0153
3739	0.1137	0.09025 to 0.1372	Yes	****	<0.0001
3747	0.03367	0.01022 to 0.05713	Yes	****	<0.0001
3748	0.04503	0.02158 to 0.06848	Yes	****	<0.0001
3749	0.02376	0.0003099 to 0.04722	Yes	*	0.0366
3750	0.02967	0.006215 to 0.05312	Yes	****	<0.0001
3754	0.05361	0.03016 to 0.07707	Yes	****	<0.0001
3755	0.05282	0.02937 to 0.07627	Yes	****	<0.0001
3756	0.07453	0.05108 to 0.09799	Yes	****	<0.0001
3757	0.06654	0.04309 to 0.09000	Yes	****	<0.0001
3758	0.0933	0.06985 to 0.1168	Yes	****	<0.0001
3759	0.063	0.03954 to 0.08645	Yes	****	<0.0001
3766	0.02619	0.002738 to 0.04964	Yes	**	0.0027
3771	0.0241	0.0006514 to 0.04756	Yes	*	0.0258
3772	0.04591	0.02245 to 0.06936	Yes	****	<0.0001
3773	0.03633	0.01288 to 0.05978	Yes	****	<0.0001
3778	0.02446	0.001011 to 0.04792	Yes	*	0.0178
3779	0.03672	0.01326 to 0.06017	Yes	****	<0.0001
3788	0.04063	0.01718 to 0.06409	Yes	****	<0.0001
3802	0.02847	0.005020 to 0.05193	Yes	***	0.0002

3805	0.02724	0.003786 to 0.05069	Yes	***	0.0008
3807	0.05781	0.03436 to 0.08126	Yes	****	<0.0001
3808	0.03188	0.008424 to 0.05533	Yes	****	<0.0001
3809	0.04309	0.01964 to 0.06654	Yes	****	<0.0001
3810	0.05602	0.03256 to 0.07947	Yes	****	<0.0001
3811	0.03158	0.008128 to 0.05504	Yes	****	<0.0001
3812	0.1371	0.1137 to 0.1606	Yes	****	<0.0001
3813	0.03323	0.009773 to 0.05668	Yes	****	<0.0001
3814	0.06348	0.04003 to 0.08694	Yes	****	<0.0001
3831	0.06339	0.03994 to 0.08684	Yes	****	<0.0001
3833	0.06562	0.04217 to 0.08908	Yes	****	<0.0001
3834	0.03166	0.008207 to 0.05511	Yes	****	<0.0001
3867	0.04286	0.01940 to 0.06631	Yes	****	<0.0001
3868	0.06355	0.04010 to 0.08700	Yes	****	<0.0001
3869	0.02991	0.006460 to 0.05337	Yes	****	<0.0001
3891	0.04526	0.02181 to 0.06872	Yes	****	<0.0001
3941	0.0257	0.002250 to 0.04916	Yes	**	0.0047
3948	0.02774	0.004284 to 0.05119	Yes	***	0.0004
3956	0.02686	0.003404 to 0.05031	Yes	**	0.0013
3982	0.0306	0.007150 to 0.05406	Yes	****	<0.0001
4007	0.04836	0.02490 to 0.07181	Yes	****	<0.0001
4015	0.03373	0.01028 to 0.05719	Yes	****	<0.0001
4024	0.03204	0.008582 to 0.05549	Yes	****	<0.0001
4028	0.02969	0.006234 to 0.05314	Yes	****	<0.0001
4047	0.08183	0.05838 to 0.1053	Yes	****	<0.0001
4062	0.04824	0.02479 to 0.07169	Yes	****	<0.0001
4063	0.05072	0.02727 to 0.07417	Yes	****	<0.0001
4064	0.05395	0.03050 to 0.07741	Yes	****	<0.0001
4065	0.06828	0.04483 to 0.09173	Yes	****	<0.0001
4066	0.1711	0.1477 to 0.1946	Yes	****	<0.0001
4067	0.2197	0.1962 to 0.2431	Yes	****	<0.0001
4068	0.09316	0.06970 to 0.1166	Yes	****	<0.0001
4069	0.03053	0.007073 to 0.05398	Yes	****	<0.0001
4070	0.05357	0.03012 to 0.07703	Yes	****	<0.0001
4071	0.02888	0.005422 to 0.05233	Yes	***	0.0001
4075	0.1065	0.08300 to 0.1299	Yes	****	<0.0001
4080	0.056	0.03255 to 0.07945	Yes	****	<0.0001
4081	0.03691	0.01345 to 0.06036	Yes	****	<0.0001
4082	0.0259	0.002445 to 0.04935	Yes	**	0.0038
4090	0.02846	0.005011 to 0.05192	Yes	***	0.0002
4091	0.02938	0.005929 to 0.05284	Yes	****	<0.0001
4092	0.07939	0.05593 to 0.1028	Yes	****	<0.0001
4093	0.08718	0.06373 to 0.1106	Yes	****	<0.0001
4094	0.06626	0.04281 to 0.08972	Yes	****	<0.0001
4095	0.07356	0.05010 to 0.09701	Yes	****	<0.0001

4096	0.1143	0.09087 to 0.1378	Yes	****	<0.0001
4097	0.04678	0.02333 to 0.07024	Yes	****	<0.0001
4099	0.04296	0.01951 to 0.06642	Yes	****	<0.0001
4101	0.04037	0.01692 to 0.06383	Yes	****	<0.0001
4102	0.06457	0.04112 to 0.08803	Yes	****	<0.0001
4103	0.05483	0.03138 to 0.07829	Yes	****	<0.0001
4112	0.05566	0.03221 to 0.07912	Yes	****	<0.0001
4116	0.07755	0.05409 to 0.1010	Yes	****	<0.0001
4117	0.09642	0.07297 to 0.1199	Yes	****	<0.0001
4125	0.1063	0.08288 to 0.1298	Yes	****	<0.0001
4126	0.05613	0.03267 to 0.07958	Yes	****	<0.0001
4127	0.06222	0.03877 to 0.08567	Yes	****	<0.0001
4128	0.07244	0.04899 to 0.09589	Yes	****	<0.0001
4129	0.02716	0.003703 to 0.05061	Yes	***	0.0009
4137	0.02877	0.005320 to 0.05223	Yes	***	0.0001
4138	0.06561	0.04216 to 0.08906	Yes	****	<0.0001
4139	0.07663	0.05318 to 0.1001	Yes	****	<0.0001
4140	0.05042	0.02696 to 0.07387	Yes	****	<0.0001
4150	0.03672	0.01326 to 0.06017	Yes	****	<0.0001
4154	0.0313	0.007843 to 0.05475	Yes	****	<0.0001
4156	0.03881	0.01535 to 0.06226	Yes	****	<0.0001
4157	0.03338	0.009927 to 0.05683	Yes	****	<0.0001
4158	0.0247	0.001246 to 0.04815	Yes	*	0.0139
4160	0.07611	0.05266 to 0.09956	Yes	****	<0.0001
4161	0.02665	0.003201 to 0.05011	Yes	**	0.0016
4162	0.06354	0.04008 to 0.08699	Yes	****	<0.0001
4163	0.07874	0.05529 to 0.1022	Yes	****	<0.0001
4164	0.1253	0.1018 to 0.1487	Yes	****	<0.0001
4165	0.119	0.09557 to 0.1425	Yes	****	<0.0001
4166	0.1745	0.1511 to 0.1980	Yes	****	<0.0001
4167	0.1608	0.1373 to 0.1843	Yes	****	<0.0001
4168	0.2401	0.2166 to 0.2635	Yes	****	<0.0001
4169	0.08921	0.06575 to 0.1127	Yes	****	<0.0001
4170	0.06467	0.04122 to 0.08812	Yes	****	<0.0001
4178	0.04646	0.02300 to 0.06991	Yes	****	<0.0001
4184	0.1181	0.09461 to 0.1415	Yes	****	<0.0001
4185	0.02701	0.003555 to 0.05046	Yes	**	0.0011
4186	0.05791	0.03445 to 0.08136	Yes	****	<0.0001
4190	0.02546	0.002009 to 0.04892	Yes	**	0.0061
4192	0.03876	0.01530 to 0.06221	Yes	****	<0.0001
4194	0.02824	0.004786 to 0.05169	Yes	***	0.0002
4200	0.0357	0.01225 to 0.05916	Yes	****	<0.0001
4203	0.06511	0.04166 to 0.08856	Yes	****	<0.0001
4204	0.07776	0.05431 to 0.1012	Yes	****	<0.0001
4205	0.1017	0.07820 to 0.1251	Yes	****	<0.0001

4206	0.06366	0.04021 to 0.08711	Yes	****	<0.0001
4207	0.06478	0.04132 to 0.08823	Yes	****	<0.0001
4208	0.02914	0.005683 to 0.05259	Yes	****	<0.0001
4210	0.05785	0.03440 to 0.08130	Yes	****	<0.0001
4219	0.04845	0.02500 to 0.07191	Yes	****	<0.0001
4220	0.04852	0.02507 to 0.07197	Yes	****	<0.0001
4221	0.1054	0.08191 to 0.1288	Yes	****	<0.0001
4222	0.1242	0.1008 to 0.1477	Yes	****	<0.0001
4223	0.05921	0.03575 to 0.08266	Yes	****	<0.0001
4224	0.06926	0.04580 to 0.09271	Yes	****	<0.0001
4225	0.04703	0.02358 to 0.07049	Yes	****	<0.0001
4226	0.0724	0.04895 to 0.09585	Yes	****	<0.0001
4227	0.02899	0.005541 to 0.05245	Yes	****	<0.0001
4236	0.05017	0.02671 to 0.07362	Yes	****	<0.0001
4237	0.1113	0.08784 to 0.1347	Yes	****	<0.0001
4240	0.0468	0.02335 to 0.07026	Yes	****	<0.0001
4241	0.03919	0.01574 to 0.06264	Yes	****	<0.0001
4242	0.03714	0.01369 to 0.06060	Yes	****	<0.0001
4315	0.03724	0.01379 to 0.06069	Yes	****	<0.0001
4324	0.02512	0.001670 to 0.04858	Yes	**	0.0088
4336	0.02851	0.005052 to 0.05196	Yes	***	0.0002
4339	0.04512	0.02166 to 0.06857	Yes	****	<0.0001
4348	0.02834	0.004885 to 0.05179	Yes	***	0.0002
4393	0.05546	0.03200 to 0.07891	Yes	****	<0.0001
4397	0.02593	0.002478 to 0.04938	Yes	**	0.0036
4409	0.04588	0.02243 to 0.06934	Yes	****	<0.0001
4423	0.02532	0.001865 to 0.04877	Yes	**	0.0071
4425	0.0497	0.02625 to 0.07316	Yes	****	<0.0001
4477	0.06216	0.03871 to 0.08562	Yes	****	<0.0001
4502	0.03204	0.008589 to 0.05550	Yes	****	<0.0001
4504	0.02788	0.004428 to 0.05134	Yes	***	0.0004
4509	0.03762	0.01417 to 0.06108	Yes	****	<0.0001
4510	0.03728	0.01383 to 0.06073	Yes	****	<0.0001
4518	0.06955	0.04610 to 0.09300	Yes	****	<0.0001
4567	0.02767	0.004214 to 0.05112	Yes	***	0.0005
4591	0.03338	0.009928 to 0.05683	Yes	****	<0.0001
4606	0.02706	0.003609 to 0.05052	Yes	***	0.001
4630	0.02382	0.0003640 to 0.04727	Yes	*	0.0347
4635	0.05354	0.03009 to 0.07699	Yes	****	<0.0001
4636	0.09976	0.07630 to 0.1232	Yes	****	<0.0001
4638	0.05291	0.02945 to 0.07636	Yes	****	<0.0001
4639	0.03476	0.01130 to 0.05821	Yes	****	<0.0001
4730	0.04598	0.02253 to 0.06943	Yes	****	<0.0001
4731	0.06067	0.03721 to 0.08412	Yes	****	<0.0001
4742	0.04645	0.02299 to 0.06990	Yes	****	<0.0001

4743	0.07084	0.04739 to 0.09429	Yes	****	<0.0001
4782	0.02509	0.001639 to 0.04855	Yes	**	0.0091
4783	0.06437	0.04092 to 0.08782	Yes	****	<0.0001
4785	0.0783	0.05485 to 0.1018	Yes	****	<0.0001
4786	0.07228	0.04882 to 0.09573	Yes	****	<0.0001
4787	0.07244	0.04898 to 0.09589	Yes	****	<0.0001
4788	0.1262	0.1027 to 0.1496	Yes	****	<0.0001
4789	0.05166	0.02820 to 0.07511	Yes	****	<0.0001
4790	0.07381	0.05036 to 0.09727	Yes	****	<0.0001
4791	0.07306	0.04961 to 0.09652	Yes	****	<0.0001
4792	0.02623	0.002775 to 0.04968	Yes	**	0.0026
4795	0.07338	0.04993 to 0.09684	Yes	****	<0.0001
4803	0.02402	0.0005672 to 0.04747	Yes	*	0.0282
4807	0.03042	0.006965 to 0.05387	Yes	****	<0.0001
4808	0.0456	0.02215 to 0.06905	Yes	****	<0.0001
4810	0.05993	0.03648 to 0.08339	Yes	****	<0.0001
4814	0.03367	0.01021 to 0.05712	Yes	****	<0.0001
4815	0.02746	0.004004 to 0.05091	Yes	***	0.0006
4819	0.07773	0.05428 to 0.1012	Yes	****	<0.0001
4820	0.05125	0.02779 to 0.07470	Yes	****	<0.0001
4822	0.0908	0.06734 to 0.1142	Yes	****	<0.0001
4823	0.06293	0.03947 to 0.08638	Yes	****	<0.0001
4824	0.02488	0.001423 to 0.04833	Yes	*	0.0115
4827	0.02807	0.004614 to 0.05152	Yes	***	0.0003
4829	0.08658	0.06313 to 0.1100	Yes	****	<0.0001
4845	0.04735	0.02390 to 0.07080	Yes	****	<0.0001
4848	0.05558	0.03212 to 0.07903	Yes	****	<0.0001
4849	0.09305	0.06960 to 0.1165	Yes	****	<0.0001
4850	0.04903	0.02558 to 0.07249	Yes	****	<0.0001
4853	0.06623	0.04278 to 0.08969	Yes	****	<0.0001
4854	0.03612	0.01267 to 0.05958	Yes	****	<0.0001
4891	0.03025	0.006798 to 0.05370	Yes	****	<0.0001
4892	0.08631	0.06286 to 0.1098	Yes	****	<0.0001
4893	0.1035	0.08004 to 0.1269	Yes	****	<0.0001
4895	0.09847	0.07502 to 0.1219	Yes	****	<0.0001
4896	0.05941	0.03596 to 0.08287	Yes	****	<0.0001
4897	0.04343	0.01998 to 0.06688	Yes	****	<0.0001
4899	0.0344	0.01095 to 0.05786	Yes	****	<0.0001
4900	0.1351	0.1116 to 0.1585	Yes	****	<0.0001
4901	0.05776	0.03431 to 0.08121	Yes	****	<0.0001
4902	0.02471	0.001252 to 0.04816	Yes	*	0.0138
4906	0.02558	0.002129 to 0.04904	Yes	**	0.0053
4908	0.02383	0.0003778 to 0.04728	Yes	*	0.0342
4910	0.028	0.004545 to 0.05145	Yes	***	0.0003
4923	0.03033	0.006878 to 0.05379	Yes	****	<0.0001

4924	0.03224	0.008790 to 0.05570	Yes	****	<0.0001
4967	0.03322	0.009770 to 0.05668	Yes	****	<0.0001
4968	0.02836	0.004905 to 0.05181	Yes	***	0.0002
4970	0.03532	0.01187 to 0.05878	Yes	****	<0.0001
4971	0.03204	0.008582 to 0.05549	Yes	****	<0.0001
4973	0.02685	0.003396 to 0.05030	Yes	**	0.0013
4978	0.02901	0.005559 to 0.05247	Yes	****	<0.0001
4984	0.05766	0.03421 to 0.08111	Yes	****	<0.0001
4985	0.09459	0.07113 to 0.1180	Yes	****	<0.0001
4999	0.07622	0.05277 to 0.09967	Yes	****	<0.0001
5004	0.0316	0.008144 to 0.05505	Yes	****	<0.0001
5021	0.02436	0.0009085 to 0.04782	Yes	*	0.0198
5063	0.05035	0.02690 to 0.07381	Yes	****	<0.0001
5069	0.02679	0.003332 to 0.05024	Yes	**	0.0014
5101	0.0696	0.04615 to 0.09306	Yes	****	<0.0001
5213	0.04983	0.02638 to 0.07328	Yes	****	<0.0001
5361	0.02749	0.004039 to 0.05095	Yes	***	0.0006
5521	0.1169	0.09345 to 0.1404	Yes	****	<0.0001
5524	0.04102	0.01756 to 0.06447	Yes	****	<0.0001
5590	0.04373	0.02027 to 0.06718	Yes	****	<0.0001
5596	0.04613	0.02268 to 0.06958	Yes	****	<0.0001
5601	0.06861	0.04516 to 0.09207	Yes	****	<0.0001
5603	0.02532	0.001871 to 0.04878	Yes	**	0.0071
5605	0.06508	0.04163 to 0.08854	Yes	****	<0.0001
5608	0.02558	0.002127 to 0.04903	Yes	**	0.0053
5625	0.03035	0.006898 to 0.05381	Yes	****	<0.0001
5645	0.04484	0.02139 to 0.06829	Yes	****	<0.0001
5659	0.03684	0.01338 to 0.06029	Yes	****	<0.0001
5666	0.08276	0.05931 to 0.1062	Yes	****	<0.0001
5668	0.0348	0.01135 to 0.05826	Yes	****	<0.0001
5669	0.04867	0.02521 to 0.07212	Yes	****	<0.0001
5681	0.02383	0.0003751 to 0.04728	Yes	*	0.0343
5695	0.03155	0.008092 to 0.05500	Yes	****	<0.0001
5788	0.03781	0.01435 to 0.06126	Yes	****	<0.0001
5819	0.03124	0.007783 to 0.05469	Yes	****	<0.0001
5876	0.04139	0.01793 to 0.06484	Yes	****	<0.0001
5879	0.03292	0.009469 to 0.05638	Yes	****	<0.0001
5882	0.03224	0.008788 to 0.05569	Yes	****	<0.0001
5883	0.03593	0.01248 to 0.05939	Yes	****	<0.0001
5890	0.03621	0.01276 to 0.05966	Yes	****	<0.0001
5891	0.06301	0.03955 to 0.08646	Yes	****	<0.0001
5893	0.0284	0.004947 to 0.05185	Yes	***	0.0002
5909	0.03212	0.008663 to 0.05557	Yes	****	<0.0001
5936	0.02979	0.006341 to 0.05325	Yes	****	<0.0001
5951	0.02721	0.003754 to 0.05066	Yes	***	0.0008

5988	0.04826	0.02480 to 0.07171	Yes	****	<0.0001
5990	0.0629	0.03945 to 0.08636	Yes	****	<0.0001
6012	0.02948	0.006025 to 0.05293	Yes	****	<0.0001
6024	0.02786	0.004404 to 0.05131	Yes	***	0.0004
6060	0.03258	0.009123 to 0.05603	Yes	****	<0.0001
6078	0.03794	0.01449 to 0.06139	Yes	****	<0.0001
6081	0.03937	0.01592 to 0.06282	Yes	****	<0.0001
6094	0.02414	0.0006868 to 0.04759	Yes	*	0.0249
6104	0.06547	0.04201 to 0.08892	Yes	****	<0.0001
6114	0.04269	0.01923 to 0.06614	Yes	****	<0.0001
6155	0.0926	0.06914 to 0.1161	Yes	****	<0.0001
6182	0.02512	0.001664 to 0.04857	Yes	**	0.0089
6184	0.05993	0.03648 to 0.08339	Yes	****	<0.0001
6185	0.04332	0.01987 to 0.06677	Yes	****	<0.0001
6213	0.02419	0.0007374 to 0.04764	Yes	*	0.0236
6253	0.02481	0.001361 to 0.04827	Yes	*	0.0123
6330	0.05356	0.03011 to 0.07701	Yes	****	<0.0001
6333	0.535	0.5115 to 0.5584	Yes	****	<0.0001
6356	0.1001	0.07666 to 0.1236	Yes	****	<0.0001
6365	0.02751	0.004061 to 0.05097	Yes	***	0.0006
6378	0.0266	0.003143 to 0.05005	Yes	**	0.0017
6380	0.0379	0.01445 to 0.06136	Yes	****	<0.0001
6382	0.0388	0.01535 to 0.06226	Yes	****	<0.0001
6411	0.02672	0.003269 to 0.05018	Yes	**	0.0015
6677	0.02514	0.001688 to 0.04859	Yes	**	0.0086
6683	0.02752	0.004068 to 0.05098	Yes	***	0.0006
6684	0.06604	0.04258 to 0.08949	Yes	****	<0.0001
6685	0.1077	0.08427 to 0.1312	Yes	****	<0.0001
6686	0.05298	0.02953 to 0.07644	Yes	****	<0.0001
6688	0.08152	0.05806 to 0.1050	Yes	****	<0.0001
6690	0.02802	0.004567 to 0.05147	Yes	***	0.0003
6693	0.04323	0.01978 to 0.06668	Yes	****	<0.0001
6694	0.06782	0.04436 to 0.09127	Yes	****	<0.0001
6695	0.1453	0.1218 to 0.1687	Yes	****	<0.0001
6696	0.03003	0.006579 to 0.05349	Yes	****	<0.0001
6697	0.02877	0.005315 to 0.05222	Yes	***	0.0001
6698	0.06032	0.03686 to 0.08377	Yes	****	<0.0001
6699	0.03297	0.009520 to 0.05643	Yes	****	<0.0001
6701	0.03221	0.008757 to 0.05566	Yes	****	<0.0001
6770	0.03105	0.007592 to 0.05450	Yes	****	<0.0001
6781	0.03015	0.006698 to 0.05360	Yes	****	<0.0001
6802	0.04321	0.01976 to 0.06666	Yes	****	<0.0001
6804	0.05557	0.03212 to 0.07903	Yes	****	<0.0001
6805	0.07692	0.05347 to 0.1004	Yes	****	<0.0001
6806	0.06388	0.04043 to 0.08734	Yes	****	<0.0001

6807	0.02599	0.002537 to 0.04944	Yes	**	0.0034
6808	0.03508	0.01163 to 0.05853	Yes	****	<0.0001
6812	0.05124	0.02779 to 0.07469	Yes	****	<0.0001
6815	0.02388	0.0004254 to 0.04733	Yes	*	0.0326
6816	0.0337	0.01025 to 0.05715	Yes	****	<0.0001
6824	0.0493	0.02585 to 0.07276	Yes	****	<0.0001
6825	0.09573	0.07227 to 0.1192	Yes	****	<0.0001
6828	0.05586	0.03241 to 0.07931	Yes	****	<0.0001
6830	0.02826	0.004805 to 0.05171	Yes	***	0.0002
6831	0.07094	0.04748 to 0.09439	Yes	****	<0.0001
6832	0.07947	0.05602 to 0.1029	Yes	****	<0.0001
6833	0.1154	0.09191 to 0.1388	Yes	****	<0.0001
6834	0.08159	0.05814 to 0.1050	Yes	****	<0.0001
6835	0.03972	0.01627 to 0.06317	Yes	****	<0.0001
6836	0.03971	0.01626 to 0.06317	Yes	****	<0.0001
6838	0.05801	0.03455 to 0.08146	Yes	****	<0.0001
6839	0.06163	0.03817 to 0.08508	Yes	****	<0.0001
6842	0.02909	0.005637 to 0.05254	Yes	****	<0.0001
6847	0.02909	0.005641 to 0.05255	Yes	****	<0.0001
6848	0.1737	0.1503 to 0.1972	Yes	****	<0.0001
6849	0.0999	0.07644 to 0.1234	Yes	****	<0.0001
6850	0.1041	0.08066 to 0.1276	Yes	****	<0.0001
6851	0.02448	0.001024 to 0.04793	Yes	*	0.0175
6864	0.04383	0.02038 to 0.06728	Yes	****	<0.0001
6865	0.07016	0.04671 to 0.09361	Yes	****	<0.0001
6901	0.02666	0.003210 to 0.05012	Yes	**	0.0016
6914	0.03811	0.01465 to 0.06156	Yes	****	<0.0001
6930	0.0301	0.006642 to 0.05355	Yes	****	<0.0001
6931	0.05461	0.03116 to 0.07807	Yes	****	<0.0001
6933	0.03208	0.008628 to 0.05553	Yes	****	<0.0001
6936	0.08639	0.06294 to 0.1098	Yes	****	<0.0001
6938	0.03332	0.009863 to 0.05677	Yes	****	<0.0001
6949	0.02755	0.004094 to 0.05100	Yes	***	0.0006
6952	0.04093	0.01748 to 0.06438	Yes	****	<0.0001
6953	0.0414	0.01794 to 0.06485	Yes	****	<0.0001
6955	0.04608	0.02263 to 0.06953	Yes	****	<0.0001
6957	0.0265	0.003043 to 0.04995	Yes	**	0.0019
6959	0.04859	0.02513 to 0.07204	Yes	****	<0.0001
6963	0.02857	0.005116 to 0.05202	Yes	***	0.0002
6965	0.02717	0.003718 to 0.05063	Yes	***	0.0009
6966	0.04216	0.01870 to 0.06561	Yes	****	<0.0001
6985	0.04391	0.02046 to 0.06736	Yes	****	<0.0001
6986	0.07655	0.05310 to 0.1000	Yes	****	<0.0001
6987	0.059	0.03555 to 0.08246	Yes	****	<0.0001
6988	0.04744	0.02398 to 0.07089	Yes	****	<0.0001

6989	0.04277	0.01931 to 0.06622	Yes	****	<0.0001
6992	0.0235	4.969e-005 to 0.04696	Yes	*	0.0476
7001	0.02759	0.004135 to 0.05104	Yes	***	0.0005
7005	0.02653	0.003077 to 0.04998	Yes	**	0.0018
7019	0.033	0.009546 to 0.05645	Yes	****	<0.0001
7021	0.04319	0.01974 to 0.06664	Yes	****	<0.0001
7022	0.03282	0.009366 to 0.05627	Yes	****	<0.0001
7023	0.03288	0.009423 to 0.05633	Yes	****	<0.0001
7025	0.04991	0.02646 to 0.07336	Yes	****	<0.0001
7044	0.05545	0.03200 to 0.07891	Yes	****	<0.0001
7100	0.03504	0.01159 to 0.05850	Yes	****	<0.0001
7102	0.03249	0.009035 to 0.05594	Yes	****	<0.0001
7107	0.05424	0.03079 to 0.07769	Yes	****	<0.0001
7109	0.04367	0.02021 to 0.06712	Yes	****	<0.0001
7110	0.03454	0.01109 to 0.05799	Yes	****	<0.0001
7112	0.02541	0.001960 to 0.04887	Yes	**	0.0064
7113	0.02451	0.001060 to 0.04797	Yes	*	0.0169
7121	0.0494	0.02594 to 0.07285	Yes	****	<0.0001
7123	0.031	0.007549 to 0.05446	Yes	****	<0.0001
7125	0.04459	0.02113 to 0.06804	Yes	****	<0.0001
7126	0.03105	0.007594 to 0.05450	Yes	****	<0.0001
7128	0.03656	0.01310 to 0.06001	Yes	****	<0.0001
7130	0.07456	0.05111 to 0.09802	Yes	****	<0.0001
7149	0.04405	0.02060 to 0.06750	Yes	****	<0.0001
7151	0.03495	0.01150 to 0.05840	Yes	****	<0.0001
7166	0.03706	0.01361 to 0.06052	Yes	****	<0.0001
7284	0.06104	0.03759 to 0.08449	Yes	****	<0.0001
7286	0.1249	0.1014 to 0.1484	Yes	****	<0.0001
7288	0.03279	0.009342 to 0.05625	Yes	****	<0.0001
7289	0.03455	0.01110 to 0.05801	Yes	****	<0.0001
7306	0.0476	0.02415 to 0.07106	Yes	****	<0.0001
7307	0.03041	0.006960 to 0.05387	Yes	****	<0.0001
7315	0.02788	0.004428 to 0.05133	Yes	***	0.0004
7324	0.02632	0.002871 to 0.04978	Yes	**	0.0023
7325	0.03938	0.01593 to 0.06283	Yes	****	<0.0001
7327	0.04414	0.02069 to 0.06759	Yes	****	<0.0001
7328	0.09489	0.07143 to 0.1183	Yes	****	<0.0001
7331	0.1115	0.08806 to 0.1350	Yes	****	<0.0001
7332	0.07341	0.04996 to 0.09686	Yes	****	<0.0001
7333	0.08758	0.06413 to 0.1110	Yes	****	<0.0001
7334	0.06808	0.04463 to 0.09154	Yes	****	<0.0001
7335	0.03395	0.01049 to 0.05740	Yes	****	<0.0001
7337	0.03239	0.008941 to 0.05585	Yes	****	<0.0001
7353	0.02924	0.005789 to 0.05270	Yes	****	<0.0001
7379	0.03736	0.01391 to 0.06081	Yes	****	<0.0001

7381	0.02799	0.004535 to 0.05144	Yes	***	0.0003
7383	0.02532	0.001871 to 0.04878	Yes	**	0.0071
7410	0.04355	0.02010 to 0.06700	Yes	****	<0.0001
7411	0.02835	0.004895 to 0.05180	Yes	***	0.0002
7414	0.05431	0.03086 to 0.07777	Yes	****	<0.0001
7416	0.2024	0.1789 to 0.2258	Yes	****	<0.0001
7445	0.03873	0.01528 to 0.06219	Yes	****	<0.0001
7553	0.03285	0.009401 to 0.05631	Yes	****	<0.0001
7584	0.04233	0.01887 to 0.06578	Yes	****	<0.0001
7585	0.05694	0.03349 to 0.08039	Yes	****	<0.0001
7586	0.06735	0.04390 to 0.09080	Yes	****	<0.0001
7587	0.0509	0.02744 to 0.07435	Yes	****	<0.0001
7607	0.0387	0.01525 to 0.06216	Yes	****	<0.0001
7622	0.04167	0.01821 to 0.06512	Yes	****	<0.0001
7625	0.05915	0.03570 to 0.08261	Yes	****	<0.0001
7645	0.04048	0.01702 to 0.06393	Yes	****	<0.0001
7651	0.02659	0.003136 to 0.05004	Yes	**	0.0017
7674	0.02935	0.005894 to 0.05280	Yes	****	<0.0001
7675	0.04195	0.01850 to 0.06541	Yes	****	<0.0001
7677	0.03818	0.01473 to 0.06164	Yes	****	<0.0001
7680	0.02665	0.003196 to 0.05010	Yes	**	0.0016
7699	0.05523	0.03177 to 0.07868	Yes	****	<0.0001
7712	0.06825	0.04479 to 0.09170	Yes	****	<0.0001
7723	0.03704	0.01358 to 0.06049	Yes	****	<0.0001
7868	0.04624	0.02279 to 0.06970	Yes	****	<0.0001
7889	0.0348	0.01135 to 0.05826	Yes	****	<0.0001
7896	0.02797	0.004513 to 0.05142	Yes	***	0.0003
7907	0.03768	0.01422 to 0.06113	Yes	****	<0.0001
7908	0.03373	0.01028 to 0.05718	Yes	****	<0.0001
7928	0.02893	0.005480 to 0.05239	Yes	***	0.0001
7950	0.0418	0.01835 to 0.06525	Yes	****	<0.0001
7958	0.06015	0.03670 to 0.08361	Yes	****	<0.0001
7965	0.02691	0.003461 to 0.05037	Yes	**	0.0012
7981	0.03899	0.01553 to 0.06244	Yes	****	<0.0001
7985	0.03664	0.01318 to 0.06009	Yes	****	<0.0001
7987	0.08859	0.06514 to 0.1120	Yes	****	<0.0001
8007	0.04946	0.02601 to 0.07292	Yes	****	<0.0001
8009	0.06668	0.04323 to 0.09014	Yes	****	<0.0001
8010	0.08085	0.05739 to 0.1043	Yes	****	<0.0001
8011	0.02973	0.006273 to 0.05318	Yes	****	<0.0001
8028	0.07866	0.05520 to 0.1021	Yes	****	<0.0001
8049	0.02663	0.003177 to 0.05008	Yes	**	0.0016
8070	0.02925	0.005799 to 0.05271	Yes	****	<0.0001
8131	0.03386	0.01041 to 0.05732	Yes	****	<0.0001
8155	0.03193	0.008479 to 0.05539	Yes	****	<0.0001

8159	0.05256	0.02911 to 0.07601	Yes	****	<0.0001
8197	0.06686	0.04340 to 0.09031	Yes	****	<0.0001
8310	0.02652	0.003071 to 0.04998	Yes	**	0.0018
8369	0.02422	0.0007695 to 0.04768	Yes	*	0.0229
8468	0.02671	0.003261 to 0.05017	Yes	**	0.0015
8478	0.0322	0.008747 to 0.05565	Yes	****	<0.0001
8519	0.03153	0.008073 to 0.05498	Yes	****	<0.0001
8543	0.03331	0.009859 to 0.05677	Yes	****	<0.0001
8594	0.04156	0.01810 to 0.06501	Yes	****	<0.0001
8625	0.02427	0.0008146 to 0.04772	Yes	*	0.0218
8628	0.04102	0.01756 to 0.06447	Yes	****	<0.0001
8792	0.02641	0.002953 to 0.04986	Yes	**	0.0021
8864	0.0507	0.02725 to 0.07416	Yes	****	<0.0001
8890	0.0268	0.003343 to 0.05025	Yes	**	0.0014
8924	0.06402	0.04057 to 0.08748	Yes	****	<0.0001
8925	0.03287	0.009420 to 0.05633	Yes	****	<0.0001
8929	0.03216	0.008702 to 0.05561	Yes	****	<0.0001
8938	0.02878	0.005329 to 0.05224	Yes	***	0.0001
8942	0.02429	0.0008391 to 0.04775	Yes	*	0.0213
8945	0.08586	0.06240 to 0.1093	Yes	****	<0.0001
8948	0.05931	0.03586 to 0.08276	Yes	****	<0.0001
8973	0.03042	0.006966 to 0.05387	Yes	****	<0.0001
8975	0.02521	0.001753 to 0.04866	Yes	**	0.008
8982	0.0252	0.001746 to 0.04865	Yes	**	0.0081
9049	0.04862	0.02517 to 0.07207	Yes	****	<0.0001
9053	0.02507	0.001614 to 0.04852	Yes	**	0.0093
9074	0.03263	0.009177 to 0.05608	Yes	****	<0.0001
9076	0.04939	0.02594 to 0.07285	Yes	****	<0.0001
9077	0.05805	0.03460 to 0.08151	Yes	****	<0.0001
9183	0.05425	0.03080 to 0.07770	Yes	****	<0.0001
9302	0.03487	0.01141 to 0.05832	Yes	****	<0.0001
9309	0.02652	0.003062 to 0.04997	Yes	**	0.0019
9614	0.02434	0.0008889 to 0.04780	Yes	*	0.0202
9622	0.0386	0.01514 to 0.06205	Yes	****	<0.0001
9992	0.02638	0.002925 to 0.04983	Yes	**	0.0022
10060	0.03436	0.01091 to 0.05782	Yes	****	<0.0001
10495	0.03957	0.01611 to 0.06302	Yes	****	<0.0001
11791	0.04858	0.02513 to 0.07204	Yes	****	<0.0001
13604	0.034	0.01055 to 0.05745	Yes	****	<0.0001
14814	0.09126	0.06780 to 0.1147	Yes	****	<0.0001
16880	0.106	0.08259 to 0.1295	Yes	****	<0.0001
17764	0.0524	0.02894 to 0.07585	Yes	****	<0.0001
18927	0.03222	0.008764 to 0.05567	Yes	****	<0.0001
20131	0.04124	0.01779 to 0.06470	Yes	****	<0.0001
20628	0.03592	0.01247 to 0.05938	Yes	****	<0.0001

20924	0.09278	0.06933 to 0.1162	Yes	****	<0.0001
21294	0.058	0.03454 to 0.08145	Yes	****	<0.0001
21423	0.0596	0.03615 to 0.08305	Yes	****	<0.0001
21636	0.1197	0.09621 to 0.1431	Yes	****	<0.0001
21747	0.137	0.1136 to 0.1605	Yes	****	<0.0001
21751	0.7746	0.7511 to 0.7980	Yes	****	<0.0001
21867	0.05621	0.03276 to 0.07967	Yes	****	<0.0001
22480	0.2231	0.1996 to 0.2466	Yes	****	<0.0001
22517	0.07141	0.04796 to 0.09486	Yes	****	<0.0001
22584	0.1394	0.1160 to 0.1629	Yes	****	<0.0001
22683	0.04393	0.02048 to 0.06738	Yes	****	<0.0001
22688	0.09667	0.07322 to 0.1201	Yes	****	<0.0001
22695	0.04551	0.02206 to 0.06896	Yes	****	<0.0001
23156	0.0709	0.04745 to 0.09436	Yes	****	<0.0001
23381	0.1103	0.08680 to 0.1337	Yes	****	<0.0001
23387	0.07047	0.04702 to 0.09392	Yes	****	<0.0001
23918	0.1031	0.07960 to 0.1265	Yes	****	<0.0001
23922	0.2451	0.2216 to 0.2685	Yes	****	<0.0001
23929	0.3039	0.2805 to 0.3274	Yes	****	<0.0001
23959	0.04584	0.02239 to 0.06929	Yes	****	<0.0001
24426	0.05788	0.03443 to 0.08133	Yes	****	<0.0001
24903	0.04166	0.01821 to 0.06512	Yes	****	<0.0001
25794	0.05553	0.03208 to 0.07898	Yes	****	<0.0001
25803	0.03895	0.01549 to 0.06240	Yes	****	<0.0001
25887	0.09814	0.07468 to 0.1216	Yes	****	<0.0001
25941	0.08654	0.06308 to 0.1100	Yes	****	<0.0001
26355	0.02953	0.006080 to 0.05299	Yes	****	<0.0001
26360	0.04576	0.02230 to 0.06921	Yes	****	<0.0001
26438	0.06898	0.04553 to 0.09243	Yes	****	<0.0001
26461	0.04209	0.01864 to 0.06554	Yes	****	<0.0001
26578	0.1759	0.1524 to 0.1993	Yes	****	<0.0001
26630	0.02602	0.002563 to 0.04947	Yes	**	0.0033
26719	0.02864	0.005183 to 0.05209	Yes	***	0.0002
26765	0.02843	0.004980 to 0.05189	Yes	***	0.0002
26923	0.06478	0.04133 to 0.08823	Yes	****	<0.0001
26956	0.2591	0.2356 to 0.2825	Yes	****	<0.0001
26969	0.02623	0.002781 to 0.04969	Yes	**	0.0026
26970	0.1283	0.1049 to 0.1518	Yes	****	<0.0001
26972	0.03626	0.01280 to 0.05971	Yes	****	<0.0001
27023	0.1626	0.1392 to 0.1861	Yes	****	<0.0001
27062	0.06318	0.03973 to 0.08663	Yes	****	<0.0001
27105	0.3056	0.2822 to 0.3291	Yes	****	<0.0001
27221	0.04227	0.01882 to 0.06572	Yes	****	<0.0001
27291	0.02724	0.003787 to 0.05069	Yes	***	0.0008
27314	0.1754	0.1519 to 0.1988	Yes	****	<0.0001

27355	0.03551	0.01206 to 0.05896	Yes	****	<0.0001
27414	0.02671	0.003257 to 0.05016	Yes	**	0.0015
27470	0.07867	0.05522 to 0.1021	Yes	****	<0.0001
27494	0.04061	0.01716 to 0.06407	Yes	****	<0.0001
27566	0.1104	0.08691 to 0.1338	Yes	****	<0.0001
27932	0.05603	0.03257 to 0.07948	Yes	****	<0.0001
27935	0.2755	0.2520 to 0.2989	Yes	****	<0.0001
27942	0.04769	0.02424 to 0.07114	Yes	****	<0.0001
27943	0.0354	0.01194 to 0.05885	Yes	****	<0.0001
28255	0.03524	0.01179 to 0.05870	Yes	****	<0.0001
28313	0.2027	0.1792 to 0.2261	Yes	****	<0.0001
28318	0.2737	0.2502 to 0.2971	Yes	****	<0.0001
28324	0.04423	0.02078 to 0.06768	Yes	****	<0.0001
28325	0.07814	0.05469 to 0.1016	Yes	****	<0.0001
28327	0.03028	0.006828 to 0.05373	Yes	****	<0.0001
28332	0.05654	0.03309 to 0.07999	Yes	****	<0.0001
28815	0.09059	0.06714 to 0.1140	Yes	****	<0.0001
28845	0.107	0.08355 to 0.1305	Yes	****	<0.0001
28953	0.08758	0.06413 to 0.1110	Yes	****	<0.0001
28958	0.4892	0.4657 to 0.5126	Yes	****	<0.0001
28960	0.1477	0.1242 to 0.1711	Yes	****	<0.0001
29268	0.1524	0.1290 to 0.1759	Yes	****	<0.0001
29331	-0.02781	-0.05127 to -0.004358	Yes	***	0.0004
29347	0.1529	0.1294 to 0.1763	Yes	****	<0.0001
29650	0.3139	0.2905 to 0.3374	Yes	****	<0.0001
29655	0.2434	0.2199 to 0.2668	Yes	****	<0.0001
29665	0.02369	0.0002318 to 0.04714	Yes	*	0.0396
29666	0.03415	0.01069 to 0.05760	Yes	****	<0.0001
29667	0.02832	0.004868 to 0.05177	Yes	***	0.0002
29692	0.04903	0.02557 to 0.07248	Yes	****	<0.0001
29717	0.02516	0.001709 to 0.04862	Yes	**	0.0084
29802	0.02347	1.653e-005 to 0.04692	Yes	*	0.0492
29813	0.02592	0.002469 to 0.04938	Yes	**	0.0037
29825	0.02391	0.0004591 to 0.04737	Yes	*	0.0315
29969	0.02803	0.004577 to 0.05148	Yes	***	0.0003
29987	0.02668	0.003225 to 0.05013	Yes	**	0.0016
30029	0.02734	0.003887 to 0.05079	Yes	***	0.0007
30088	0.04734	0.02389 to 0.07080	Yes	****	<0.0001
30089	0.06937	0.04592 to 0.09282	Yes	****	<0.0001
30090	0.02409	0.0006374 to 0.04754	Yes	*	0.0262
30091	0.0268	0.003343 to 0.05025	Yes	**	0.0014
30104	0.02817	0.004714 to 0.05162	Yes	***	0.0003
30192	0.027	0.003543 to 0.05045	Yes	**	0.0011
30217	0.02362	0.0001711 to 0.04708	Yes	*	0.0421
30237	0.02467	0.001217 to 0.04812	Yes	*	0.0143

30308	0.02773	0.004278 to 0.05118	Yes	***	0.0005
30316	0.02589	0.002441 to 0.04935	Yes	**	0.0038
30323	0.02991	0.006453 to 0.05336	Yes	****	<0.0001
30330	0.02711	0.003658 to 0.05057	Yes	***	0.0009
30336	0.02577	0.002318 to 0.04922	Yes	**	0.0043
30337	0.02651	0.003054 to 0.04996	Yes	**	0.0019
30366	0.02476	0.001311 to 0.04822	Yes	*	0.0129
30367	0.02726	0.003805 to 0.05071	Yes	***	0.0008
30374	0.02427	0.0008137 to 0.04772	Yes	*	0.0218
30433	0.02452	0.001070 to 0.04798	Yes	*	0.0167
30435	0.02936	0.005906 to 0.05281	Yes	****	<0.0001
30452	0.0282	0.004747 to 0.05165	Yes	***	0.0003
30454	0.02507	0.001612 to 0.04852	Yes	**	0.0094
30461	0.03073	0.007275 to 0.05418	Yes	****	<0.0001
30463	0.0516	0.02815 to 0.07505	Yes	****	<0.0001
30464	0.03296	0.009507 to 0.05641	Yes	****	<0.0001
30465	0.03202	0.008566 to 0.05547	Yes	****	<0.0001
30466	0.02673	0.003277 to 0.05018	Yes	**	0.0015
30469	0.029	0.005545 to 0.05245	Yes	****	<0.0001
30489	0.02606	0.002606 to 0.04951	Yes	**	0.0031
30498	0.04026	0.01680 to 0.06371	Yes	****	<0.0001
30523	0.04045	0.01700 to 0.06390	Yes	****	<0.0001
30529	0.03567	0.01222 to 0.05912	Yes	****	<0.0001
30530	0.04178	0.01833 to 0.06524	Yes	****	<0.0001
30550	0.02721	0.003759 to 0.05067	Yes	***	0.0008
30551	0.03386	0.01040 to 0.05731	Yes	****	<0.0001
30554	0.0421	0.01864 to 0.06555	Yes	****	<0.0001
30567	0.05014	0.02668 to 0.07359	Yes	****	<0.0001
30569	0.02404	0.0005851 to 0.04749	Yes	*	0.0277
30609	0.0243	0.0008498 to 0.04776	Yes	*	0.021
30656	0.03874	0.01529 to 0.06220	Yes	****	<0.0001
30663	0.02875	0.005298 to 0.05220	Yes	***	0.0001
30672	0.02497	0.001512 to 0.04842	Yes	*	0.0104
30681	0.02737	0.003915 to 0.05082	Yes	***	0.0007
30687	0.0247	0.001248 to 0.04815	Yes	*	0.0138
30693	0.02413	0.0006797 to 0.04759	Yes	*	0.0251
30759	0.02942	0.005963 to 0.05287	Yes	****	<0.0001
30760	0.02507	0.001614 to 0.04852	Yes	**	0.0093
30776	0.02407	0.0006199 to 0.04753	Yes	*	0.0267
30784	0.07697	0.05351 to 0.1004	Yes	****	<0.0001
30786	0.03295	0.009497 to 0.05640	Yes	****	<0.0001
30787	0.0341	0.01064 to 0.05755	Yes	****	<0.0001
30822	0.02388	0.0004300 to 0.04734	Yes	*	0.0324
30823	0.02981	0.006356 to 0.05326	Yes	****	<0.0001
30878	0.02348	2.264e-005 to 0.04693	Yes	*	0.0489

30924	0.02977	0.006313 to 0.05322	Yes	****	<0.0001

Genetic Region
5' UTR
TRS-L
5' UTR
nsp1

nsp2

nsp3

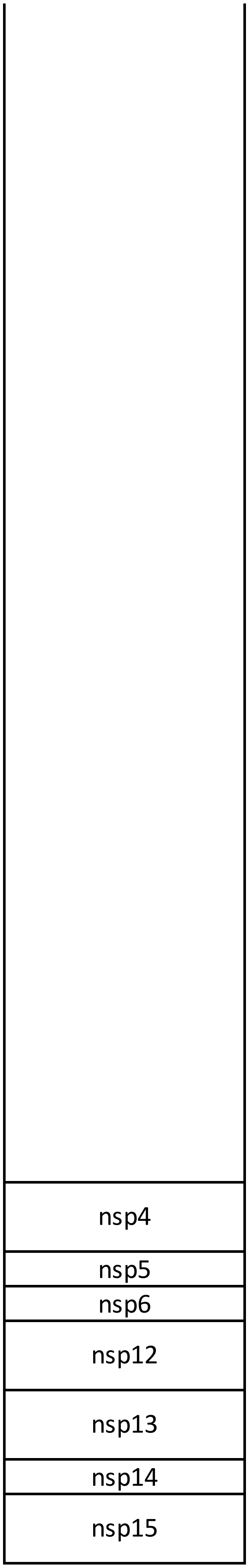
nsp4
nsp5
nsp6
nsp12
nsp13
nsp14
nsp15

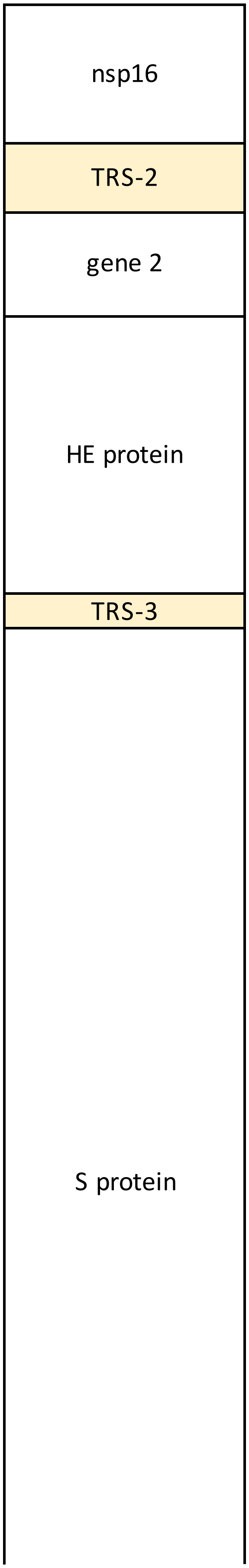
nsp16
TRS-2
gene 2
HE protein
TRS-3
S protein

TRS-4
untranslated region
Gene 4
TRS-5
E protein
TRS-6
M protein
TRS-7

N protein

Position	Mean Diff.	95.00% CI of diff.	Significant?	Summary	Adjusted P Value	Gene
64	0.06062	0.03947 to 0.08177	Yes	****	<0.0001	TRS-L
66	0.5902	0.5690 to 0.6113	Yes	****	<0.0001	
67	0.06445	0.04330 to 0.08561	Yes	****	<0.0001	
68	0.08157	0.06042 to 0.1027	Yes	****	<0.0001	
69	0.4869	0.4658 to 0.5081	Yes	****	<0.0001	
70	0.02203	0.0008775 to 0.04318	Yes	*	0.0185	
71	0.5224	0.5013 to 0.5436	Yes	****	<0.0001	
75	0.2152	0.1940 to 0.2363	Yes	****	<0.0001	
1583	0.02121	6.071e-005 to 0.04236	Yes	*	0.0468	nsp2
1626	0.03041	0.009262 to 0.05156	Yes	****	<0.0001	
2173	0.03052	0.009367 to 0.05167	Yes	****	<0.0001	
2540	0.02439	0.003236 to 0.04554	Yes	**	0.001	
2881	0.02337	0.002223 to 0.04452	Yes	**	0.0037	
3812	0.02374	0.002587 to 0.04489	Yes	**	0.0023	
4066	0.02461	0.003461 to 0.04576	Yes	***	0.0008	
4094	0.02986	0.008713 to 0.05101	Yes	****	<0.0001	
4111	-0.02955	-0.05070 to -0.008399	Yes	****	<0.0001	
4164	0.02225	0.001094 to 0.04340	Yes	*	0.0143	
4166	0.03324	0.01209 to 0.05440	Yes	****	<0.0001	
4167	0.02459	0.003437 to 0.04574	Yes	***	0.0008	
4168	0.02929	0.008140 to 0.05044	Yes	****	<0.0001	
4226	0.02518	0.004030 to 0.04633	Yes	***	0.0004	
4234	-0.02829	-0.04944 to -0.007139	Yes	****	<0.0001	
4422	-0.02244	-0.04359 to -0.001289	Yes	*	0.0114	
4618	-0.05567	-0.07682 to -0.03452	Yes	****	<0.0001	
4731	-0.03008	-0.05123 to -0.008925	Yes	****	<0.0001	
4810	-0.04062	-0.06177 to -0.01947	Yes	****	<0.0001	
4813	-0.04759	-0.06874 to -0.02644	Yes	****	<0.0001	
4817	-0.02264	-0.04379 to -0.001484	Yes	**	0.009	
4820	-0.04967	-0.07082 to -0.02852	Yes	****	<0.0001	
4880	-0.02415	-0.04531 to -0.003004	Yes	**	0.0014	
4887	-0.03327	-0.05442 to -0.01212	Yes	****	<0.0001	
4895	-0.02815	-0.04930 to -0.007001	Yes	****	<0.0001	
4900	-0.02458	-0.04573 to -0.003426	Yes	***	0.0008	
5000	-0.02146	-0.04261 to -0.0003041	Yes	*	0.0356	
5586	-0.02391	-0.04506 to -0.002755	Yes	**	0.0019	
5590	-0.03013	-0.05128 to -0.008978	Yes	****	<0.0001	
5594	-0.03561	-0.05676 to -0.01446	Yes	****	<0.0001	
5595	-0.0977	-0.1189 to -0.07655	Yes	****	<0.0001	
5598	-0.1313	-0.1525 to -0.1102	Yes	****	<0.0001	
5601	-0.06997	-0.09112 to -0.04882	Yes	****	<0.0001	
5666	-0.02321	-0.04436 to -0.002063	Yes	**	0.0045	
5883	-0.02617	-0.04732 to -0.005020	Yes	****	<0.0001	
5892	-0.04057	-0.06172 to -0.01941	Yes	****	<0.0001	

5893	-0.02312	-0.04427 to -0.001969	Yes	**	0.005
5978	-0.02288	-0.04403 to -0.001729	Yes	**	0.0067
5983	-0.02867	-0.04982 to -0.007521	Yes	****	<0.0001
5985	-0.02594	-0.04709 to -0.004787	Yes	***	0.0001
5987	-0.0303	-0.05145 to -0.009151	Yes	****	<0.0001
5988	-0.06449	-0.08564 to -0.04334	Yes	****	<0.0001
5995	-0.03403	-0.05518 to -0.01288	Yes	****	<0.0001
6012	-0.02916	-0.05031 to -0.008007	Yes	****	<0.0001
6013	-0.04347	-0.06462 to -0.02232	Yes	****	<0.0001
6078	-0.02552	-0.04667 to -0.004364	Yes	***	0.0002
6083	-0.02632	-0.04747 to -0.005164	Yes	****	<0.0001
6110	-0.02262	-0.04377 to -0.001470	Yes	**	0.0092
6113	-0.04095	-0.06210 to -0.01979	Yes	****	<0.0001
6114	-0.02438	-0.04553 to -0.003232	Yes	**	0.001
6115	-0.02752	-0.04867 to -0.006366	Yes	****	<0.0001
6140	-0.02443	-0.04558 to -0.003281	Yes	***	0.001
6184	-0.0658	-0.08695 to -0.04465	Yes	****	<0.0001
6298	-0.02505	-0.04620 to -0.003901	Yes	***	0.0004
6333	0.09839	0.07724 to 0.1195	Yes	****	<0.0001
6341	-0.02705	-0.04820 to -0.005897	Yes	****	<0.0001
6348	-0.03842	-0.05957 to -0.01727	Yes	****	<0.0001
6351	-0.02328	-0.04443 to -0.002131	Yes	**	0.0041
6356	-0.06155	-0.08270 to -0.04040	Yes	****	<0.0001
6357	-0.02469	-0.04585 to -0.003543	Yes	***	0.0007
6368	-0.02409	-0.04524 to -0.002935	Yes	**	0.0015
6371	-0.02607	-0.04722 to -0.004922	Yes	***	0.0001
6372	-0.02883	-0.04998 to -0.007680	Yes	****	<0.0001
6373	-0.02195	-0.04310 to -0.0007947	Yes	*	0.0204
6374	-0.05083	-0.07198 to -0.02968	Yes	****	<0.0001
6375	-0.02568	-0.04683 to -0.004532	Yes	***	0.0002
6376	-0.07353	-0.09468 to -0.05238	Yes	****	<0.0001
6377	-0.05124	-0.07239 to -0.03009	Yes	****	<0.0001
6378	-0.04719	-0.06834 to -0.02604	Yes	****	<0.0001
6379	-0.02129	-0.04245 to -0.0001432	Yes	*	0.0427
6380	-0.03036	-0.05151 to -0.009212	Yes	****	<0.0001
6382	-0.05049	-0.07165 to -0.02934	Yes	****	<0.0001
6384	-0.05441	-0.07556 to -0.03326	Yes	****	<0.0001
6412	-0.03684	-0.05799 to -0.01569	Yes	****	<0.0001
6596	-0.03063	-0.05178 to -0.009475	Yes	****	<0.0001
6597	-0.05713	-0.07828 to -0.03598	Yes	****	<0.0001
6598	-0.05257	-0.07372 to -0.03142	Yes	****	<0.0001
6603	-0.0411	-0.06225 to -0.01995	Yes	****	<0.0001
6605	-0.0498	-0.07095 to -0.02865	Yes	****	<0.0001
6606	-0.02202	-0.04317 to -0.0008641	Yes	*	0.0188
6607	-0.0415	-0.06266 to -0.02035	Yes	****	<0.0001

6609	-0.06446	-0.08561 to -0.04331	Yes	****	<0.0001
6626	-0.04197	-0.06312 to -0.02081	Yes	****	<0.0001
6637	-0.03217	-0.05332 to -0.01102	Yes	****	<0.0001
6638	-0.04158	-0.06273 to -0.02043	Yes	****	<0.0001
6640	-0.02442	-0.04557 to -0.003271	Yes	***	0.001
6641	-0.09451	-0.1157 to -0.07336	Yes	****	<0.0001
6671	-0.02747	-0.04863 to -0.006324	Yes	****	<0.0001
6673	-0.04796	-0.06912 to -0.02681	Yes	****	<0.0001
6674	-0.02449	-0.04564 to -0.003342	Yes	***	0.0009
6675	-0.0411	-0.06225 to -0.01995	Yes	****	<0.0001
6676	-0.07208	-0.09323 to -0.05093	Yes	****	<0.0001
6677	-0.0325	-0.05366 to -0.01135	Yes	****	<0.0001
6681	-0.04895	-0.07010 to -0.02780	Yes	****	<0.0001
6682	-0.03584	-0.05699 to -0.01469	Yes	****	<0.0001
6683	-0.02765	-0.04881 to -0.006503	Yes	****	<0.0001
6684	-0.08963	-0.1108 to -0.06848	Yes	****	<0.0001
6685	-0.04853	-0.06968 to -0.02738	Yes	****	<0.0001
6686	-0.08602	-0.1072 to -0.06487	Yes	****	<0.0001
6687	-0.05497	-0.07612 to -0.03382	Yes	****	<0.0001
6688	-0.06679	-0.08794 to -0.04564	Yes	****	<0.0001
6689	-0.05714	-0.07830 to -0.03599	Yes	****	<0.0001
6690	-0.06342	-0.08457 to -0.04227	Yes	****	<0.0001
6691	-0.07129	-0.09244 to -0.05014	Yes	****	<0.0001
6692	-0.06357	-0.08472 to -0.04242	Yes	****	<0.0001
6693	-0.02728	-0.04843 to -0.006127	Yes	****	<0.0001
6694	-0.05183	-0.07298 to -0.03068	Yes	****	<0.0001
6695	-0.08552	-0.1067 to -0.06437	Yes	****	<0.0001
6696	-0.05094	-0.07209 to -0.02979	Yes	****	<0.0001
6697	-0.02408	-0.04523 to -0.002924	Yes	**	0.0015
6698	-0.0451	-0.06625 to -0.02395	Yes	****	<0.0001
6699	-0.06129	-0.08244 to -0.04013	Yes	****	<0.0001
6700	-0.05222	-0.07337 to -0.03107	Yes	****	<0.0001
6701	-0.05902	-0.08017 to -0.03786	Yes	****	<0.0001
6702	-0.0226	-0.04375 to -0.001452	Yes	**	0.0094
6724	-0.03499	-0.05614 to -0.01384	Yes	****	<0.0001
6725	-0.02669	-0.04784 to -0.005538	Yes	****	<0.0001
6739	-0.02309	-0.04424 to -0.001936	Yes	**	0.0052
6740	-0.03749	-0.05864 to -0.01634	Yes	****	<0.0001
6741	-0.0246	-0.04575 to -0.003444	Yes	***	0.0008
6742	-0.0258	-0.04695 to -0.004653	Yes	***	0.0002
6760	-0.02411	-0.04526 to -0.002955	Yes	**	0.0015
6767	-0.04006	-0.06121 to -0.01891	Yes	****	<0.0001
6768	-0.0357	-0.05685 to -0.01455	Yes	****	<0.0001
6784	-0.04234	-0.06349 to -0.02119	Yes	****	<0.0001
6785	-0.0323	-0.05345 to -0.01114	Yes	****	<0.0001

6786	-0.02547	-0.04662 to -0.004318	Yes	***	0.0002
6787	-0.02947	-0.05062 to -0.008315	Yes	****	<0.0001
6788	-0.05459	-0.07574 to -0.03344	Yes	****	<0.0001
6796	-0.03202	-0.05317 to -0.01086	Yes	****	<0.0001
6797	-0.02336	-0.04451 to -0.002213	Yes	**	0.0037
6798	-0.02334	-0.04449 to -0.002184	Yes	**	0.0038
6799	-0.0395	-0.06065 to -0.01835	Yes	****	<0.0001
6800	-0.03312	-0.05428 to -0.01197	Yes	****	<0.0001
6801	-0.02859	-0.04974 to -0.007440	Yes	****	<0.0001
6802	-0.03291	-0.05406 to -0.01176	Yes	****	<0.0001
6803	-0.02833	-0.04948 to -0.007174	Yes	****	<0.0001
6804	-0.0598	-0.08095 to -0.03865	Yes	****	<0.0001
6805	-0.05188	-0.07303 to -0.03073	Yes	****	<0.0001
6806	-0.04961	-0.07076 to -0.02846	Yes	****	<0.0001
6807	-0.06742	-0.08858 to -0.04627	Yes	****	<0.0001
6808	-0.04493	-0.06608 to -0.02378	Yes	****	<0.0001
6809	-0.02729	-0.04844 to -0.006136	Yes	****	<0.0001
6810	-0.04106	-0.06221 to -0.01991	Yes	****	<0.0001
6824	-0.04907	-0.07022 to -0.02792	Yes	****	<0.0001
6825	-0.05233	-0.07348 to -0.03117	Yes	****	<0.0001
6826	-0.0613	-0.08245 to -0.04015	Yes	****	<0.0001
6827	-0.05667	-0.07782 to -0.03552	Yes	****	<0.0001
6828	-0.08214	-0.1033 to -0.06099	Yes	****	<0.0001
6829	-0.05204	-0.07319 to -0.03089	Yes	****	<0.0001
6830	-0.07963	-0.1008 to -0.05848	Yes	****	<0.0001
6831	-0.09369	-0.1148 to -0.07254	Yes	****	<0.0001
6832	-0.1054	-0.1265 to -0.08423	Yes	****	<0.0001
6833	-0.06875	-0.08990 to -0.04760	Yes	****	<0.0001
6834	-0.02613	-0.04728 to -0.004977	Yes	****	<0.0001
6835	-0.06345	-0.08460 to -0.04230	Yes	****	<0.0001
6838	-0.04493	-0.06608 to -0.02378	Yes	****	<0.0001
6839	-0.07738	-0.09854 to -0.05623	Yes	****	<0.0001
6841	-0.02967	-0.05082 to -0.008522	Yes	****	<0.0001
6847	-0.0297	-0.05085 to -0.008550	Yes	****	<0.0001
6848	-0.08976	-0.1109 to -0.06861	Yes	****	<0.0001
6849	-0.07937	-0.1005 to -0.05822	Yes	****	<0.0001
6850	-0.0624	-0.08355 to -0.04124	Yes	****	<0.0001
6851	-0.02758	-0.04873 to -0.006433	Yes	****	<0.0001
6852	-0.02489	-0.04604 to -0.003742	Yes	***	0.0005
6862	-0.0256	-0.04675 to -0.004450	Yes	***	0.0002
6922	-0.03206	-0.05321 to -0.01091	Yes	****	<0.0001
6928	-0.02139	-0.04254 to -0.0002427	Yes	*	0.0382
6930	-0.05013	-0.07128 to -0.02898	Yes	****	<0.0001
6931	-0.04386	-0.06502 to -0.02271	Yes	****	<0.0001
6932	-0.06572	-0.08687 to -0.04457	Yes	****	<0.0001

6933	-0.03751	-0.05866 to -0.01636	Yes	****	<0.0001
6934	-0.02242	-0.04357 to -0.001271	Yes	*	0.0116
6936	-0.0269	-0.04805 to -0.005745	Yes	****	<0.0001
6941	-0.02749	-0.04864 to -0.006338	Yes	****	<0.0001
6951	-0.03112	-0.05227 to -0.009966	Yes	****	<0.0001
6952	-0.06974	-0.09089 to -0.04858	Yes	****	<0.0001
6953	-0.03647	-0.05762 to -0.01532	Yes	****	<0.0001
6954	-0.03952	-0.06067 to -0.01837	Yes	****	<0.0001
6955	-0.07575	-0.09691 to -0.05460	Yes	****	<0.0001
6956	-0.08744	-0.1086 to -0.06629	Yes	****	<0.0001
6958	-0.02503	-0.04618 to -0.003881	Yes	***	0.0004
6959	-0.0341	-0.05525 to -0.01295	Yes	****	<0.0001
6964	-0.05381	-0.07496 to -0.03266	Yes	****	<0.0001
6965	-0.04446	-0.06561 to -0.02331	Yes	****	<0.0001
6966	-0.03532	-0.05647 to -0.01417	Yes	****	<0.0001
6983	-0.02942	-0.05057 to -0.008271	Yes	****	<0.0001
6984	-0.02402	-0.04517 to -0.002873	Yes	**	0.0016
6986	-0.04639	-0.06755 to -0.02524	Yes	****	<0.0001
6987	-0.02781	-0.04896 to -0.006662	Yes	****	<0.0001
6988	-0.02955	-0.05070 to -0.008400	Yes	****	<0.0001
7005	-0.02453	-0.04568 to -0.003379	Yes	***	0.0008
7106	-0.03128	-0.05244 to -0.01013	Yes	****	<0.0001
7107	-0.05062	-0.07177 to -0.02947	Yes	****	<0.0001
7108	-0.02168	-0.04284 to -0.0005332	Yes	*	0.0275
7109	-0.04051	-0.06166 to -0.01936	Yes	****	<0.0001
7110	-0.04984	-0.07099 to -0.02869	Yes	****	<0.0001
7111	-0.03083	-0.05198 to -0.009677	Yes	****	<0.0001
7117	-0.03765	-0.05880 to -0.01650	Yes	****	<0.0001
7118	-0.03993	-0.06108 to -0.01878	Yes	****	<0.0001
7120	-0.142	-0.1631 to -0.1208	Yes	****	<0.0001
7121	-0.04954	-0.07069 to -0.02839	Yes	****	<0.0001
7123	-0.0493	-0.07046 to -0.02815	Yes	****	<0.0001
7124	-0.04942	-0.07057 to -0.02826	Yes	****	<0.0001
7125	-0.04597	-0.06712 to -0.02482	Yes	****	<0.0001
7126	-0.0631	-0.08425 to -0.04195	Yes	****	<0.0001
7127	-0.03336	-0.05451 to -0.01220	Yes	****	<0.0001
7128	-0.05692	-0.07807 to -0.03577	Yes	****	<0.0001
7129	-0.03116	-0.05231 to -0.01000	Yes	****	<0.0001
7130	-0.09596	-0.1171 to -0.07481	Yes	****	<0.0001
7140	-0.02329	-0.04444 to -0.002136	Yes	**	0.0041
7147	-0.03068	-0.05183 to -0.009530	Yes	****	<0.0001
7148	-0.05121	-0.07236 to -0.03006	Yes	****	<0.0001
7149	-0.03606	-0.05722 to -0.01491	Yes	****	<0.0001
7150	-0.04233	-0.06348 to -0.02118	Yes	****	<0.0001
7151	-0.0539	-0.07505 to -0.03275	Yes	****	<0.0001

7152	-0.05752	-0.07867 to -0.03637	Yes	****	<0.0001	
7153	-0.02337	-0.04452 to -0.002214	Yes	**	0.0037	
7154	-0.02968	-0.05083 to -0.008530	Yes	****	<0.0001	
7168	-0.03262	-0.05377 to -0.01147	Yes	****	<0.0001	
7169	-0.03401	-0.05516 to -0.01286	Yes	****	<0.0001	
7170	-0.02538	-0.04653 to -0.004228	Yes	***	0.0003	
7270	-0.02676	-0.04791 to -0.005609	Yes	****	<0.0001	
7271	-0.057	-0.07815 to -0.03585	Yes	****	<0.0001	
7284	-0.04345	-0.06460 to -0.02229	Yes	****	<0.0001	
7285	-0.03221	-0.05336 to -0.01106	Yes	****	<0.0001	
7286	-0.1055	-0.1267 to -0.08436	Yes	****	<0.0001	
7287	-0.03837	-0.05952 to -0.01722	Yes	****	<0.0001	nsp3
7288	-0.05666	-0.07781 to -0.03551	Yes	****	<0.0001	
7289	-0.055	-0.07615 to -0.03385	Yes	****	<0.0001	
7304	-0.05896	-0.08011 to -0.03781	Yes	****	<0.0001	
7308	-0.02647	-0.04762 to -0.005316	Yes	****	<0.0001	
7309	-0.02189	-0.04304 to -0.0007396	Yes	*	0.0217	
7310	-0.05254	-0.07369 to -0.03139	Yes	****	<0.0001	
7311	-0.04958	-0.07073 to -0.02843	Yes	****	<0.0001	
7324	-0.02779	-0.04894 to -0.006640	Yes	****	<0.0001	
7325	-0.03488	-0.05604 to -0.01373	Yes	****	<0.0001	
7326	-0.04331	-0.06447 to -0.02216	Yes	****	<0.0001	
7327	-0.04009	-0.06124 to -0.01894	Yes	****	<0.0001	
7328	-0.065	-0.08615 to -0.04384	Yes	****	<0.0001	
7329	-0.04628	-0.06743 to -0.02513	Yes	****	<0.0001	
7331	-0.05339	-0.07454 to -0.03224	Yes	****	<0.0001	
7332	-0.0527	-0.07385 to -0.03154	Yes	****	<0.0001	
7333	-0.1032	-0.1243 to -0.08201	Yes	****	<0.0001	
7334	-0.07244	-0.09359 to -0.05129	Yes	****	<0.0001	
7335	-0.0587	-0.07985 to -0.03754	Yes	****	<0.0001	
7336	-0.06567	-0.08682 to -0.04452	Yes	****	<0.0001	
7337	-0.05518	-0.07633 to -0.03403	Yes	****	<0.0001	
7354	-0.025	-0.04615 to -0.003848	Yes	***	0.0005	
7366	-0.02989	-0.05104 to -0.008741	Yes	****	<0.0001	
7379	-0.02728	-0.04843 to -0.006125	Yes	****	<0.0001	
7380	-0.04216	-0.06331 to -0.02101	Yes	****	<0.0001	
7381	-0.0405	-0.06165 to -0.01935	Yes	****	<0.0001	
7382	-0.02691	-0.04806 to -0.005757	Yes	****	<0.0001	
7383	-0.02501	-0.04616 to -0.003860	Yes	***	0.0005	
7395	-0.0223	-0.04345 to -0.001146	Yes	*	0.0135	
7407	-0.02998	-0.05113 to -0.008833	Yes	****	<0.0001	
7409	-0.04789	-0.06904 to -0.02674	Yes	****	<0.0001	
7410	-0.02908	-0.05024 to -0.007933	Yes	****	<0.0001	
7411	-0.05542	-0.07657 to -0.03426	Yes	****	<0.0001	
7412	-0.03673	-0.05788 to -0.01558	Yes	****	<0.0001	

7413	-0.03534	-0.05649 to -0.01419	Yes	****	<0.0001
7445	-0.02687	-0.04802 to -0.005718	Yes	****	<0.0001
7446	-0.03879	-0.05994 to -0.01764	Yes	****	<0.0001
7462	-0.03432	-0.05547 to -0.01317	Yes	****	<0.0001
7516	-0.03249	-0.05364 to -0.01134	Yes	****	<0.0001
7542	-0.02986	-0.05101 to -0.008710	Yes	****	<0.0001
7544	-0.02688	-0.04803 to -0.005725	Yes	****	<0.0001
7545	-0.02277	-0.04392 to -0.001617	Yes	**	0.0077
7550	-0.02713	-0.04828 to -0.005978	Yes	****	<0.0001
7551	-0.0325	-0.05365 to -0.01135	Yes	****	<0.0001
7552	-0.09178	-0.1129 to -0.07063	Yes	****	<0.0001
7553	-0.04327	-0.06442 to -0.02212	Yes	****	<0.0001
7555	-0.02629	-0.04744 to -0.005139	Yes	****	<0.0001
7582	-0.03067	-0.05182 to -0.009520	Yes	****	<0.0001
7583	-0.02466	-0.04581 to -0.003507	Yes	***	0.0007
7584	-0.03789	-0.05904 to -0.01674	Yes	****	<0.0001
7586	-0.05424	-0.07539 to -0.03309	Yes	****	<0.0001
7587	-0.02994	-0.05109 to -0.008788	Yes	****	<0.0001
7609	-0.02194	-0.04309 to -0.0007877	Yes	*	0.0205
7613	-0.02639	-0.04754 to -0.005237	Yes	****	<0.0001
7621	-0.0262	-0.04735 to -0.005050	Yes	****	<0.0001
7622	-0.05952	-0.08067 to -0.03837	Yes	****	<0.0001
7623	-0.02537	-0.04652 to -0.004220	Yes	***	0.0003
7624	-0.04444	-0.06559 to -0.02329	Yes	****	<0.0001
7625	-0.02905	-0.05020 to -0.007896	Yes	****	<0.0001
7637	-0.03645	-0.05760 to -0.01530	Yes	****	<0.0001
7648	-0.06015	-0.08130 to -0.03900	Yes	****	<0.0001
7649	-0.02187	-0.04302 to -0.0007198	Yes	*	0.0222
7650	-0.03144	-0.05259 to -0.01028	Yes	****	<0.0001
7651	-0.07104	-0.09219 to -0.04989	Yes	****	<0.0001
7652	-0.02893	-0.05008 to -0.007778	Yes	****	<0.0001
7673	-0.07428	-0.09544 to -0.05313	Yes	****	<0.0001
7674	-0.0554	-0.07655 to -0.03425	Yes	****	<0.0001
7675	-0.07874	-0.09989 to -0.05759	Yes	****	<0.0001
7677	-0.02474	-0.04589 to -0.003592	Yes	***	0.0006
7680	-0.02224	-0.04339 to -0.001090	Yes	*	0.0144
7685	-0.05041	-0.07156 to -0.02926	Yes	****	<0.0001
7686	-0.03298	-0.05413 to -0.01183	Yes	****	<0.0001
7687	-0.02151	-0.04266 to -0.0003594	Yes	*	0.0335
7690	-0.02631	-0.04746 to -0.005154	Yes	****	<0.0001
7698	-0.03587	-0.05703 to -0.01472	Yes	****	<0.0001
7712	-0.04845	-0.06960 to -0.02730	Yes	****	<0.0001
7715	-0.03703	-0.05818 to -0.01588	Yes	****	<0.0001
7843	-0.02619	-0.04734 to -0.005040	Yes	****	<0.0001
7889	-0.04056	-0.06171 to -0.01941	Yes	****	<0.0001

7890	-0.06151	-0.08266 to -0.04036	Yes	****	<0.0001
7907	-0.02375	-0.04490 to -0.002600	Yes	**	0.0023
7908	-0.03864	-0.05979 to -0.01749	Yes	****	<0.0001
7978	-0.02654	-0.04769 to -0.005385	Yes	****	<0.0001
7980	-0.02339	-0.04454 to -0.002241	Yes	**	0.0036
7981	-0.03188	-0.05303 to -0.01073	Yes	****	<0.0001
7982	-0.04584	-0.06699 to -0.02469	Yes	****	<0.0001
7988	-0.02528	-0.04643 to -0.004125	Yes	***	0.0003
7996	-0.0231	-0.04425 to -0.001948	Yes	**	0.0051
7997	-0.02229	-0.04344 to -0.001135	Yes	*	0.0137
7998	-0.02568	-0.04683 to -0.004529	Yes	***	0.0002
8005	-0.0333	-0.05445 to -0.01215	Yes	****	<0.0001
8006	-0.05019	-0.07134 to -0.02904	Yes	****	<0.0001
8007	-0.06633	-0.08748 to -0.04518	Yes	****	<0.0001
8008	-0.05616	-0.07731 to -0.03501	Yes	****	<0.0001
8009	-0.09182	-0.1130 to -0.07067	Yes	****	<0.0001
8010	-0.07905	-0.1002 to -0.05790	Yes	****	<0.0001
8033	-0.02268	-0.04384 to -0.001533	Yes	**	0.0085
8034	-0.02464	-0.04579 to -0.003486	Yes	***	0.0007
8051	-0.04299	-0.06414 to -0.02184	Yes	****	<0.0001
8053	-0.04014	-0.06129 to -0.01899	Yes	****	<0.0001
8071	-0.09458	-0.1157 to -0.07343	Yes	****	<0.0001
8086	-0.02584	-0.04700 to -0.004694	Yes	***	0.0001
8089	-0.02151	-0.04266 to -0.0003608	Yes	*	0.0334
8151	-0.03261	-0.05376 to -0.01145	Yes	****	<0.0001
8153	-0.04015	-0.06130 to -0.01900	Yes	****	<0.0001
8154	-0.02621	-0.04736 to -0.005057	Yes	****	<0.0001
8155	-0.06462	-0.08577 to -0.04347	Yes	****	<0.0001
8156	-0.03587	-0.05702 to -0.01472	Yes	****	<0.0001
8157	-0.03002	-0.05117 to -0.008865	Yes	****	<0.0001
8159	-0.03818	-0.05933 to -0.01703	Yes	****	<0.0001
8160	-0.02532	-0.04647 to -0.004164	Yes	***	0.0003
8170	-0.02817	-0.04932 to -0.007018	Yes	****	<0.0001
8195	-0.02646	-0.04761 to -0.005307	Yes	****	<0.0001
8197	-0.04037	-0.06152 to -0.01922	Yes	****	<0.0001
8355	-0.02722	-0.04837 to -0.006071	Yes	****	<0.0001
8361	-0.02769	-0.04884 to -0.006535	Yes	****	<0.0001
8381	-0.0224	-0.04356 to -0.001253	Yes	*	0.0119
8462	-0.04518	-0.06633 to -0.02403	Yes	****	<0.0001
8464	-0.0634	-0.08456 to -0.04225	Yes	****	<0.0001
8465	-0.07402	-0.09517 to -0.05287	Yes	****	<0.0001
8466	-0.05101	-0.07216 to -0.02986	Yes	****	<0.0001
8467	-0.03244	-0.05359 to -0.01128	Yes	****	<0.0001
8469	-0.0358	-0.05695 to -0.01465	Yes	****	<0.0001
8471	-0.04344	-0.06459 to -0.02229	Yes	****	<0.0001

8484	0.02196	0.0008090 to 0.04311	Yes	*	0.02
8511	-0.03519	-0.05634 to -0.01404	Yes	****	<0.0001
8513	-0.02229	-0.04344 to -0.001138	Yes	*	0.0136
8515	-0.02117	-0.04232 to -2.131e-005	Yes	*	0.0488
8518	-0.02329	-0.04444 to -0.002141	Yes	**	0.0041
8520	-0.04071	-0.06186 to -0.01956	Yes	****	<0.0001
8537	-0.07866	-0.09981 to -0.05751	Yes	****	<0.0001
8538	-0.04247	-0.06362 to -0.02132	Yes	****	<0.0001
8539	-0.05927	-0.08042 to -0.03812	Yes	****	<0.0001
8543	-0.09668	-0.1178 to -0.07553	Yes	****	<0.0001
8544	-0.03837	-0.05952 to -0.01721	Yes	****	<0.0001
8545	-0.0307	-0.05186 to -0.009554	Yes	****	<0.0001
8546	-0.03644	-0.05759 to -0.01529	Yes	****	<0.0001
8547	-0.02131	-0.04246 to -0.0001601	Yes	*	0.0419
8555	-0.04811	-0.06927 to -0.02696	Yes	****	<0.0001
8595	-0.02139	-0.04254 to -0.0002387	Yes	*	0.0384
8606	-0.02817	-0.04932 to -0.007020	Yes	****	<0.0001
8629	-0.0271	-0.04825 to -0.005948	Yes	****	<0.0001
8640	-0.02382	-0.04497 to -0.002671	Yes	**	0.0021
8733	-0.03537	-0.05652 to -0.01422	Yes	****	<0.0001
8746	-0.03471	-0.05586 to -0.01356	Yes	****	<0.0001
8749	-0.03566	-0.05681 to -0.01450	Yes	****	<0.0001
8750	-0.0685	-0.08965 to -0.04735	Yes	****	<0.0001
8751	-0.0579	-0.07906 to -0.03675	Yes	****	<0.0001
8752	-0.03688	-0.05803 to -0.01573	Yes	****	<0.0001
8753	-0.03972	-0.06087 to -0.01857	Yes	****	<0.0001
8754	-0.04239	-0.06354 to -0.02124	Yes	****	<0.0001
8755	-0.03448	-0.05563 to -0.01333	Yes	****	<0.0001
8772	-0.03138	-0.05253 to -0.01023	Yes	****	<0.0001
8773	-0.02408	-0.04523 to -0.002928	Yes	**	0.0015
8860	-0.02598	-0.04714 to -0.004833	Yes	***	0.0001
8863	-0.04012	-0.06127 to -0.01897	Yes	****	<0.0001
8864	-0.02596	-0.04711 to -0.004809	Yes	***	0.0001
8887	-0.02241	-0.04357 to -0.001264	Yes	*	0.0117
8888	-0.03406	-0.05521 to -0.01291	Yes	****	<0.0001
8889	-0.03889	-0.06004 to -0.01774	Yes	****	<0.0001
8890	-0.03373	-0.05488 to -0.01258	Yes	****	<0.0001
8891	-0.03948	-0.06063 to -0.01833	Yes	****	<0.0001
8916	-0.0374	-0.05855 to -0.01625	Yes	****	<0.0001
8923	-0.03446	-0.05561 to -0.01331	Yes	****	<0.0001
8924	-0.05982	-0.08097 to -0.03867	Yes	****	<0.0001
8925	-0.04301	-0.06416 to -0.02186	Yes	****	<0.0001
8926	-0.05194	-0.07310 to -0.03079	Yes	****	<0.0001
8927	-0.05873	-0.07988 to -0.03758	Yes	****	<0.0001
8928	-0.02793	-0.04908 to -0.006778	Yes	****	<0.0001

8929	-0.02625	-0.04740 to -0.005099	Yes	****	<0.0001
8930	-0.0594	-0.08055 to -0.03825	Yes	****	<0.0001
8931	-0.04812	-0.06927 to -0.02697	Yes	****	<0.0001
8932	-0.0608	-0.08195 to -0.03965	Yes	****	<0.0001
8938	-0.05169	-0.07284 to -0.03054	Yes	****	<0.0001
8942	-0.02598	-0.04713 to -0.004825	Yes	***	0.0001
8943	-0.04028	-0.06143 to -0.01913	Yes	****	<0.0001
8944	-0.02979	-0.05094 to -0.008641	Yes	****	<0.0001
8945	-0.06303	-0.08418 to -0.04188	Yes	****	<0.0001
8946	-0.06804	-0.08920 to -0.04689	Yes	****	<0.0001
8947	-0.0479	-0.06905 to -0.02675	Yes	****	<0.0001
8948	-0.102	-0.1232 to -0.08085	Yes	****	<0.0001
8949	-0.09519	-0.1163 to -0.07404	Yes	****	<0.0001
8950	-0.02447	-0.04562 to -0.003321	Yes	***	0.0009
8972	-0.02443	-0.04558 to -0.003275	Yes	***	0.001
8980	-0.0297	-0.05085 to -0.008547	Yes	****	<0.0001
8981	-0.06352	-0.08467 to -0.04237	Yes	****	<0.0001
8989	-0.05412	-0.07527 to -0.03297	Yes	****	<0.0001
9046	-0.04673	-0.06788 to -0.02558	Yes	****	<0.0001
9047	-0.04391	-0.06506 to -0.02276	Yes	****	<0.0001
9048	-0.0308	-0.05196 to -0.009653	Yes	****	<0.0001
9049	-0.03378	-0.05493 to -0.01263	Yes	****	<0.0001
9050	-0.03882	-0.05997 to -0.01767	Yes	****	<0.0001
9051	-0.02864	-0.04979 to -0.007484	Yes	****	<0.0001
9052	-0.03297	-0.05412 to -0.01182	Yes	****	<0.0001
9055	-0.0313	-0.05246 to -0.01015	Yes	****	<0.0001
9056	-0.04194	-0.06309 to -0.02078	Yes	****	<0.0001
9076	-0.04333	-0.06448 to -0.02218	Yes	****	<0.0001
9077	-0.02411	-0.04526 to -0.002958	Yes	**	0.0015
9083	-0.02895	-0.05010 to -0.007803	Yes	****	<0.0001
9091	-0.02908	-0.05024 to -0.007934	Yes	****	<0.0001
9092	-0.03644	-0.05760 to -0.01529	Yes	****	<0.0001
9098	-0.03633	-0.05748 to -0.01517	Yes	****	<0.0001
9100	-0.0212	-0.04235 to -4.712e-005	Yes	*	0.0475
9101	-0.0668	-0.08795 to -0.04565	Yes	****	<0.0001
9146	-0.02631	-0.04746 to -0.005155	Yes	****	<0.0001
9150	-0.03413	-0.05528 to -0.01297	Yes	****	<0.0001
9152	-0.03293	-0.05408 to -0.01178	Yes	****	<0.0001
9155	-0.02628	-0.04743 to -0.005128	Yes	****	<0.0001
9157	-0.02582	-0.04697 to -0.004669	Yes	***	0.0002
9168	-0.02359	-0.04474 to -0.002439	Yes	**	0.0028
9170	-0.02504	-0.04619 to -0.003885	Yes	***	0.0004
9173	-0.02116	-0.04231 to -1.271e-005	Yes	*	0.0493
9183	-0.03816	-0.05931 to -0.01701	Yes	****	<0.0001
9185	-0.02745	-0.04860 to -0.006299	Yes	****	<0.0001

9222	-0.02647	-0.04762 to -0.005318	Yes	****	<0.0001	
9299	-0.03896	-0.06011 to -0.01781	Yes	****	<0.0001	
9481	-0.02651	-0.04766 to -0.005358	Yes	****	<0.0001	
9482	-0.03008	-0.05123 to -0.008929	Yes	****	<0.0001	
9512	-0.03115	-0.05230 to -0.009999	Yes	****	<0.0001	
9527	-0.02349	-0.04465 to -0.002343	Yes	**	0.0032	
9528	-0.02255	-0.04370 to -0.001399	Yes	**	0.01	
9529	-0.02627	-0.04742 to -0.005120	Yes	****	<0.0001	
9544	-0.02579	-0.04694 to -0.004642	Yes	***	0.0002	
9558	-0.02399	-0.04514 to -0.002839	Yes	**	0.0017	
9754	-0.02574	-0.04689 to -0.004589	Yes	***	0.0002	nsp4
9843	-0.03282	-0.05397 to -0.01166	Yes	****	<0.0001	
9887	-0.02275	-0.04390 to -0.001599	Yes	**	0.0079	
9995	-0.02269	-0.04384 to -0.001539	Yes	**	0.0085	
10012	-0.02165	-0.04280 to -0.0005022	Yes	*	0.0285	
13604	0.03014	0.008984 to 0.05129	Yes	****	<0.0001	nsp9
14621	-0.04515	-0.06630 to -0.02400	Yes	****	<0.0001	nsp12
14814	0.06111	0.03996 to 0.08226	Yes	****	<0.0001	
16005	-0.02928	-0.05043 to -0.008128	Yes	****	<0.0001	
16880	0.1156	0.09441 to 0.1367	Yes	****	<0.0001	nsp13
17764	0.0471	0.02595 to 0.06825	Yes	****	<0.0001	
18914	0.02452	0.003370 to 0.04567	Yes	***	0.0009	nsp14
18927	0.03855	0.01740 to 0.05970	Yes	****	<0.0001	
20131	0.03177	0.01062 to 0.05292	Yes	****	<0.0001	nsp15
21751	0.2539	0.2327 to 0.2750	Yes	****	<0.0001	TRS-2
22480	0.03223	0.01108 to 0.05338	Yes	****	<0.0001	ORF2a
23929	0.1846	0.1634 to 0.2057	Yes	****	<0.0001	TRS-3
25941	0.02265	0.001503 to 0.04380	Yes	**	0.0088	S protein
26719	0.02223	0.001075 to 0.04338	Yes	*	0.0147	
26765	0.02238	0.001228 to 0.04353	Yes	*	0.0122	
27865	-0.02556	-0.04671 to -0.004412	Yes	***	0.0002	
27923	-0.0398	-0.06095 to -0.01865	Yes	****	<0.0001	
27925	0.04484	0.02369 to 0.06600	Yes	****	<0.0001	
27934	0.04547	0.02432 to 0.06662	Yes	****	<0.0001	TRS-4
27935	0.02684	0.005693 to 0.04799	Yes	****	<0.0001	
27942	0.2121	0.1909 to 0.2332	Yes	****	<0.0001	
28102	0.02876	0.007610 to 0.04991	Yes	****	<0.0001	ORF4b
28120	0.0282	0.007049 to 0.04935	Yes	****	<0.0001	TRS-5
28325	0.2053	0.1842 to 0.2265	Yes	****	<0.0001	
28511	0.02163	0.0004813 to 0.04278	Yes	*	0.0292	ORF5a
28704	0.02141	0.0002565 to 0.04256	Yes	*	0.0376	
28953	0.03448	0.01333 to 0.05563	Yes	****	<0.0001	
28958	0.5344	0.5133 to 0.5556	Yes	****	<0.0001	TRS-6
28960	0.3489	0.3278 to 0.3701	Yes	****	<0.0001	
29268	0.07755	0.05640 to 0.09870	Yes	****	<0.0001	

29347	0.07747	0.05632 to 0.09862	Yes	****	<0.0001	M protein
29650	0.02724	0.006087 to 0.04839	Yes	****	<0.0001	
29655	0.03687	0.01572 to 0.05802	Yes	****	<0.0001	TRS-7
29666	0.1709	0.1498 to 0.1921	Yes	****	<0.0001	N protein
31327	-0.03401	-0.05516 to -0.01286	Yes	****	<0.0001	3' UTR
